# Quantifying the impact of inter-site heterogeneity on the distribution of ChIP-seq data

**DOI:** 10.3389/fgene.2014.00399

**Published:** 2014-11-14

**Authors:** Jonathan Cairns, Andy G. Lynch, Simon Tavaré

**Affiliations:** ^1^Nuclear Dynamics Group, The Babraham InstituteCambridge, UK; ^2^Cancer Research UK Cambridge Institute, University of CambridgeCambridge, UK

**Keywords:** ChIP-seq, Negative Binomial, mixture model, zero-inflation, high-throughput sequencing

## Abstract

Chromatin Immunoprecipitation followed by sequencing (ChIP-seq) is a valuable tool for epigenetic studies. Analysis of the data arising from ChIP-seq experiments often requires implicit or explicit statistical modeling of the read counts. The simple Poisson model is attractive, but does not provide a good fit to observed ChIP-seq data. Researchers therefore often either extend to a more general model (e.g., the Negative Binomial), and/or exclude regions of the genome that do not conform to the model. Since many modeling strategies employed for ChIP-seq data reduce to fitting a mixture of Poisson distributions, we explore the problem of inferring the optimal mixing distribution. We apply the Constrained Newton Method (CNM), which suggests the Negative Binomial - Negative Binomial (NB-NB) mixture model as a candidate for modeling ChIP-seq data. We illustrate fitting the NB-NB model with an accelerated EM algorithm on four data sets from three species. Zero-inflated models have been suggested as an approach to improve model fit for ChIP-seq data. We show that the NB-NB mixture model requires no zero-inflation and suggest that in some cases the need for zero inflation is driven by the model's inability to cope with both artifactual large read counts and the frequently observed very low read counts. We see that the CNM-based approach is a useful diagnostic for the assessment of model fit and inference in ChIP-seq data and beyond. Use of the suggested NB-NB mixture model will be of value not only when calling peaks or otherwise modeling ChIP-seq data, but also when simulating data or constructing blacklists *de novo*.

## 1. Introduction

Chromatin Immunoprecipitation followed by sequencing (ChIP-seq) is an experiment for the genome-wide location of events such as transcription factor binding sites and histone modifications (Park, 2009; Cairns et al., 2013). These events provide information on chromatin status, a topic of great interest in epigenetics. As with all sequencing assays, ChIP-seq can be represented as count data across the genome. Commonly, researchers need algorithms that find sites where the counts are larger than one would expect under a null noise model, a procedure known as “peak-calling.”

Consider read counts *x_i_*, where *i* indexes over genomic bins, for a single ChIP-seq sample. When modeling these counts as independent samples from some Poisson random variable *X*, a common problem is overdispersion—that is, the property that the observations have greater variance than allowed for by the model. In our case, the Poisson distribution requires that mean and variance are equal, an assumption that (even when ignoring between-sample variability) is violated by ChIP-seq data, impairing model fit (Spyrou et al., 2009).

The choice of distribution for *X* is important when peak-calling; in particular, underestimating the variance should decrease specificity. Indeed, many have commented on the presence of false positives in peak-calling and how this quantity varies depending on the choice of peak-caller (Landt et al., 2012).

A number of strategies exist to account for the extra variance. For example, we can allow the Poisson distribution to have site-specific means—that is, *X_i_* ~ *Pois*(λ_*i*_). This strategy requires some sort of smoothing criterion to make parameter estimation robust (Zhang et al., 2008a). It is also difficult to expand this model to account for between-sample heterogeneity.

Many researchers use more general distributions—for example, the Negative Binomial (NB) model is used by Spyrou et al. (2009); Wu et al. (2010); Cairns et al. (2011); Song and Smith (2011) and others. The log-normal Poisson model has been used to model data from other high-throughput sequencing experiments, such as Serial Analysis of Gene Expression (SAGE) (Thygesen and Zwinderman, 2006).

Other strategies involve regression. ZINBA (Rashid et al., 2011) uses an NB model whose mean is regressed against known covariates, though the dispersion is fixed. Such a strategy requires knowledge of all appropriate dependent variables to capture the full variability.

An increasingly common approach is to use blacklists (Myers et al., 2011)—regions that have unusual mappability and previously have been seen to accumulate artifacts across many ChIP-seq experiments. Reads that fall in blacklisted regions are removed.

Use of blacklists appears to improve peak-calling (Carroll et al., 2014), but has a number of downsides. Firstly, this strategy requires an organism-specific blacklist, and it is possible that the blacklist is non-exhaustive. Secondly, there is no reason to assume that enrichment cannot occur in these loci—a better alternative would be to model these regions appropriately, or perhaps separately. Thirdly, there may be sample-specific or copy-number specific events. For example, a transfected vector may artificially inflate copy number at a particular locus.

We consider the general form of the above models, taking a completely unsupervised random-effects approach. It is reasonable to retain the Poisson element in our model, since it has been shown that counts from a single site in a single library, sequenced repeatedly, follow a Poisson distribution (Marioni et al., 2008). However, by expanding to a mixture model setting, where *x_i_* is a sample from *X_i_* ~ *Pois*(Λ) and the latent mixing variable Λ satisfies Λ ~ *f*(λ), we can use *f*(λ) to capture the genome-wide variation in our sample. Indeed, if Λ has gamma distribution, then *X_i_* has NB distribution.

However, there is no biological or statistical reason, other than convenience, to believe that Λ is best modeled with the gamma distribution. We assess the fit of the NB model to the data; that is, investigate the appropriateness of the gamma distribution as a mixing distribution. Next, we use CNM to make inferences about the distribution of Λ, and see that it suggests a mixture of two distributions. Thus, we consider the NB-NB distribution and show that it provides a better fit to the data. We also consider zero inflation, and show that the poor fit of the NB distribution can be mistaken for the presence of zero-inflation in the data.

### 1.1. Data

We use four different data sets, all of which are input samples (that is, untreated data). We focus on input samples because, in order to detect sites where counts differ from noise, it is important to model the noise appropriately. However, the methods used can also be applied to treated ChIP-seq data—see Supplementary Material. Samples were chosen to represent a variety of species. In each case, we considered only the first chromosome to avoid inter-chromosome normalization issues (Schmidt et al., 2010; Ross-Innes et al., 2012).

**Table:** 

**Sample**	**Species**	**Description**	**Read lengths**	**# Reads**	**Aligner**
A	*C. Familiaris*	Normal liver tissue	45	480,843	MAQ
B	*M. Musculus*	Normal liver tissue	36	542,021	MAQ
C	*H. Sapiens*	Cancer cell line MCF7	44	633,931	BWA 0.5.5
D	*H. Sapiens*	Tumor sample BT82277	44	1,398,520	BWA 0.5.5

We partition the first chromosome into 100 bp bins, avoiding conservatively-chosen regions that span the telomeres and centromeres, as obtained from the UCSC Genome Browser at http://genome.ucsc.edu/. We did not remove duplicates—however, we found that deleting duplicates did not substantially affect our results (see Supplementary Material).

Code and data to reproduce the analysis are linked to in the Supplementary Material section.

## 2. Initial mixture models

### 2.1. Poisson

The Poisson model's Maximum Likelihood Estimate (MLE) occurs when its expected mean is equated to the observed mean: $\hat{\mu }$ = *x*.

### 2.2. Negative binomial

The MLE for the Negative Binomial (NB) distribution does not have closed form. However, we can fit the model using standard techniques, using the BFGS method implemented as *fitdistr()* in the *MASS* R package (Venables and Ripley, 2002).

Though the MLE for the dispersion of the NB distribution is biased, the bias is of the order 1/*n* (Saha and Paul, 2005) and we have enough bins in our data sets for the bias to be negligible.

### 2.3. Log-normal poisson

Here, Λ is a log-normal random variable: log(Λ) ~ *N*(μ, σ^2^). The full log-likelihood involves a complicated integral:

$$\ell (\mu ,\sigma^{2})=\sum_{i\;=\;1}^{n}\log\int_{{\mathbb{R}}^{+}}\frac{1}{\lambda \sqrt{2\pi \sigma^{2}}}\frac{\exp\left(-\frac{{\left(\log\lambda -\mu \right)}^{2}}{2\sigma^{2}}-\lambda \right)\lambda^{x_{i}}}{x_{i}!}d\lambda$$

and the MLEs have no known closed form. Therefore, to estimate the parameters of this distribution, we take the following Bayesian approach:

1. Assume a weak prior distribution for each of μ (the mean) and σ^−2^ (the precision). We use conjugate prior distributions:
  $$\begin{array}{l} \;\mu \sim N(0,10)\\ \sigma^{-2}\sim \Gamma (shape=1,rate=0.1) \end{array}$$
  The prior distributions should have very little effect on the posterior, given that we have so many data.
2. Use the Metropolis-Hastings algorithm to sample from each parameter's posterior distribution.
3. Excluding a suitably-chosen burn-in period, take the mean of the posterior samples as an approximation to the maximum a posteriori (MAP) estimate.

We perform the analysis in WinBUGS (Lunn et al., 2000).

### 2.4. Initial results

**Figure 1:**
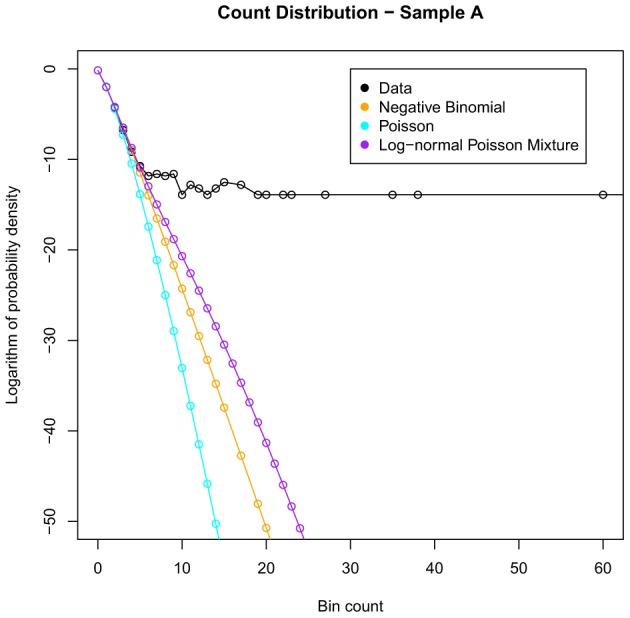
**Illustrating the best fits of the commonly used distributions to the count data for sample A**. Note that none of these distributions can model adequately the heavy tail of large bin counts.

Figure 1 shows an example of the fit of the above distributions to sample A. We see that the empirical distribution has a large tail that the fitted distributions cannot account for, which in turn affects their ability to model bins with low counts.

To explore this effect further, we investigate the properties of the underlying mixing distribution.

## 3. Mixture inference

As described in the introduction, suppose that we have multiple samples {*x_i_*; *i* = 1, …, *n*} from a variable *X* distributed according to a Poisson mixture—that is,

$$\begin{array}{l} X_{i}|\Lambda_{i}\sim Pois(\Lambda_{i})\\ \;\Lambda_{i}\sim f(\lambda ) \end{array}$$

Our aim is to estimate the density *f*(λ) from our observations {*x_i_*}.

A formulation of the general problem, where *X_i_*|Λ_*i*_ has arbitrary distribution, is given in Roueff and Rydén (2005). In the case of the Poisson distribution, it is known that $\hat{f}$(λ), the maximum likelihood estimator (MLE) of *f*(λ), consists of a finite number of point masses: that is, if we adopt the MLE $\hat{f}$(λ), then Λ is a discrete random variable, taking values from some vector ***θ*** with associated probabilities **π**, where ***θ*** and **π** both have length *L*. It is also known that *L*, the number of support points used in the MLE distribution, can never exceed the number of distinct values in our *X* samples (Laird, 1978).

A number of methods have been proposed to infer the parameters *L*, ***θ***, and **π**, including those of Tucker (1963); Boes (1966); Simar (1976); Laird (1978). Here, we use the Constrained Newton Method (CNM), described in Wang (2007).

The CNM algorithm takes an initial value for (*L*, ***θ***, **π**), then repeats the following steps until convergence:

1. **Update** (*L*, ***θ***), as follows: Suppose that *G*(λ) is our current estimate for the mixing distribution, corresponding to the current value of (*L*, ***θ***, **π**). Now, consider some new candidate support point θ^*^, and let *H*_θ^*^_(λ) be the distribution that has a point mass at θ^*^—in other words, *H*_θ^*^_ is the Dirac delta function *H*_θ^*^_(λ) = δ(λ − θ^*^). Consider the “gradient function,” the directional derivative
  $$d(\theta^{⋆};G)=\frac{\partial \ell }{\partial \epsilon }\{(1-\epsilon )G+\epsilon H_{\theta^{⋆}}\}$$
  which we can think of as the rate at which the likelihood increases, as we shift probability mass across to the new support point θ^*^.
  The value of θ^*^ that maximizes *d*(θ^*^; *G*) is added to ***θ***, and *L* is increased by one. If multiple values of θ^*^ maximize *d*(θ^*^; *G*), then we add each of them to ***θ***, increasing *L* by one for each value.
2. **Update **π**** by calculating the MLE for **π** given (***θ***, *L*). Wang (2007) show that this problem is equivalent to
  $$\pi =\underset{\pi^{\prime }}{\min}\|S\pi^{\prime }-2\times 1{\|}^{2}$$
  subject to $\sum_{i\;=\;1}^{L}\pi_{i}=1,\pi^{\prime }\geq 0$, and where $S_{ij}=\frac{\partial }{\partial \pi_{i}}\ell_{j}$ (here, ℓ_*j*_ refers to the likelihood for a single data point, *x_j_*). This minimization problem is solved using the Constrained Newton (CN) method.
3. If π_*i*_ = 0 for some i^*^, then delete π_i^*^_ and *θ*_i^*^_ and reduce *L* by one.

Wang (2007) prove theoretical convergence and demonstrate that, in practice, convergence is dramatically faster than previous algorithms designed to find *L*, ***θ***, and **π**.

CNM reported that it did not converge for data sets C or D, and we check the output in the next section. In practice, we found that the value of *L* ranged from around 4 to 11 in our input samples.

### 3.1. Distribution recovery

First, we show that the CNM estimate $\hat{f}$(λ) is appropriate for our data, by demonstrating that the empirical distribution of *X* can be recovered from the CNM estimate according to the following procedure:

1. Calculate the empirical probability density $\tilde{f}$(*x*) directly, from the data.
2. Calculate the mixing distribution MLE $\hat{f}$(λ) from the data according to the CNM algorithm.
3. Estimate the density *f*(*x*) from the mixing distribution according to
  $$\hat{f}(x)=\sum_{i\;=\;1}^{L}\pi_{i}f_{P}(x;\theta_{i})$$
  where *f_P_*(*x*; *θ*_*i*_) is the density of the Poisson distribution with mean *θ*_*i*_.

**Figure 2:**
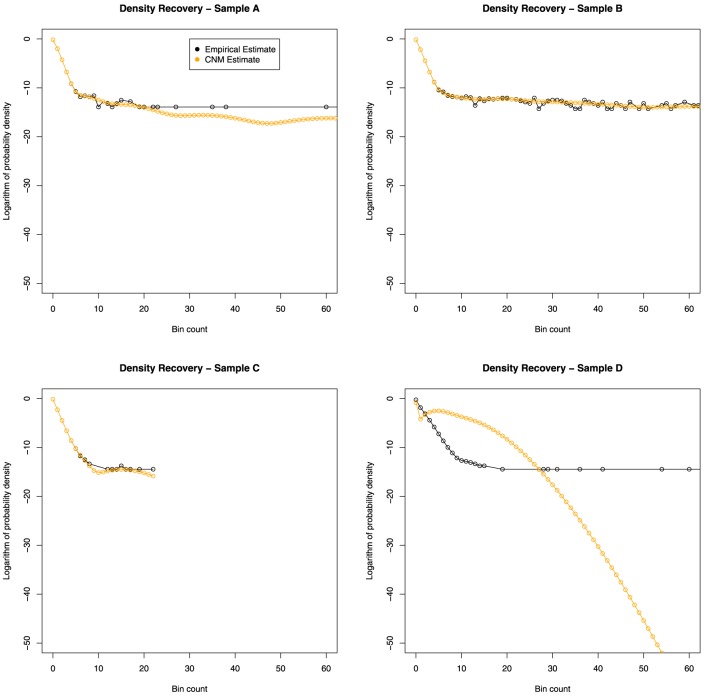
**Distribution recovery in each data set**. The empirical distribution obtained from the actual data (black line) appears to show a dramatic change in behavior around *x* = 9. While CNM reported failure to converge for samples C and D, it can be seen that sample C's distribution was adequately recovered.

If the CNM algorithm retains the key features of the true distribution *f*(*x*), then we expect $\hat{f}$(*x*) to be “similar” to the empirical distribution $\tilde{f}$(*x*). The results of this comparison are shown in Figure 2.

### 3.2. Smoothing

The MLE $\hat{f}$(λ) is desirable in the sense that it is the “best fit” to our data. However, it does not make physical sense to use $\hat{f}$(λ) when modeling, which would make Λ a discrete variable. In reality, drawing from Λ represents a complicated chain of library-preparation procedures, leading to wide variation across hundreds of millions of sites across the genome. Therefore, it is much more logical to model Λ as a continuous random variable.

As such, we adopt the following strategy to assess the fit of various distributions:

1. Fit a continuous mixing distribution $\hat{f}$_Θ_(λ) to the data.
2. Compare $\hat{f}$_Θ_(λ) with the discrete MLE $\hat{f}$(λ), obtained from CNM.

If $\hat{f}$_Θ_(λ) is flexible enough to fit our data, then it should be “similar” to the MLE $\hat{f}$(λ). Thus, we need to compare a discrete distribution, $\hat{f}$(λ), with a continuous distribution, $\hat{f}$_Θ_(λ). Methodology for making such a comparison tends to be *ad hoc*, as the general question is ill-defined.

A common strategy is to smooth out the data through “kernel smoothing”—that is, replacing each point mass in the discrete distribution with an appropriately-scaled kernel distribution, often the normal distribution. The problem with this approach is that the support of the mixing distribution's MLE contains only a handful of points, and the points become dramatically less dense at higher values. Thus, by using this approach, we end up with a distribution that is highly sensitive to the choice of length scale for the kernel.

Instead, we take the following non-parametric approach. Assume that CNM's output [the discrete distribution with support ***θ*** = (*θ*_1_, …, *θ*_*L*_) and associated probabilities **π** = (π_1_, …, π_*L*_)] has been generated from a “true” distribution *f*(λ) through the following process:

1. Take some partition ***P*** = (*P*_1_ = 0, *P*_2_, *P*_3_, …, *P_L_*, ∞) of the interval [0, ∞].
2. For each *i* ∈ {1, …, *L*}, replace the probability mass of *f*(λ) that lies in the interval [*P_i_*, *P*_*i* + 1_) with an equivalent point mass of size π_*i*_ = ∫^*P*_*i*+1_^_*P*_*i*__*f*(*u*)*du* placed at any point *θ*_*i*_ within that interval.

Now, consider the true distribution's CDF, *F*(λ) = ∫^λ^_0_*f*(*u*)*du*. For *i* > 1, we have:

(1) $$F(P_{i})=\int_{0}^{P_{i}}f(u)du=\sum_{j\;=\;1}^{i\;-\;1}\int_{P_{j}}^{P_{j+1}}f(u)du=\sum_{j\;=\;1}^{i-1}\pi_{j}$$

For the case *i* = 1, we set

(2) $$F(P_{1})\;=\;F(0)\;=\;0$$

Note that Equation 2 assumes that Λ is not zero-inflated.

We can now place bounds on the value of *F*(*θ*_*i*_). *θ*_*i*_ must lie in [*P_i_*, *P*_*i* + 1_), by assumption. Therefore, *F*(*θ*_*i*_) must lie in [*F*(*P_i_*), *F*(*P*_*i* + 1_)), since *F*(λ) is a CDF and is therefore an increasing function of λ. Thus, for *i* > 1, by Equation (1) we have

(3) $$\sum_{j\;=\;1}^{i-1}\pi_{j}\leq F(\theta_{i})<\sum_{j\;=\;1}^{i}\pi_{j}$$

and for *i* = 1, we have

(4) $$0\leq F(\theta_{1})<\pi_{1}$$

We can use these upper and lower bounds to assess the fit of various candidate mixing distributions to the observed mixing distribution.

**Figure 3:**
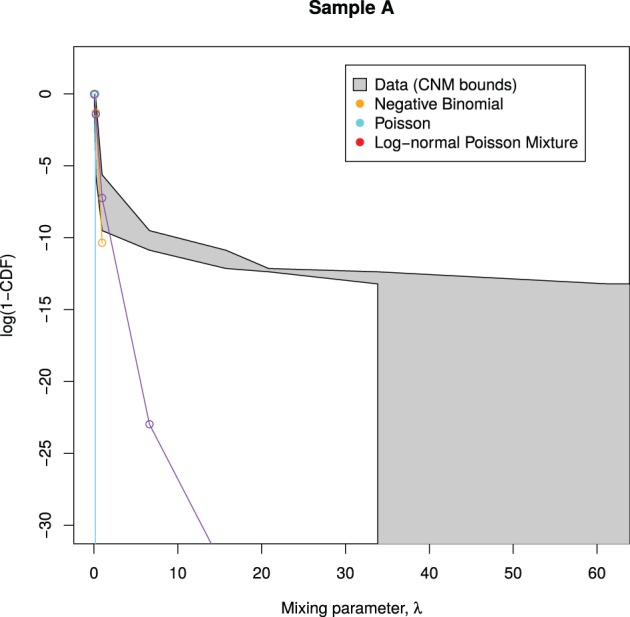
**Candidate mixing distributions, and their consistency with the CNM-derived mixing distribution**. For clarity, we plot log(1 − *CDF*) where *CDF* is the Cumulative Density Function of Λ, and values are calculated only at CNM's λ support points. A mixing distribution that is consistent with CNM's predicted mixing distribution, as derived in Section 3.2, would have a line contained within the shaded region, with the black lines representing upper and lower bounds. Here, the lines do not stay within the bounds, indicating that all of the models deviate from CNM's prediction.

Figure 3 shows an example of this procedure, as applied to sample A. We see that all of the candidate mixing distributions considered thus far violate the CNM bounds early on, indicating that these distributions cannot cope with large counts. This motivates the selection of a mixing distribution that can stay within the bounds.

### 3.3. NB-NB mixture

The curves plotted in Figure 3 have insufficient curvature to accommodate the sharp turn present in the shaded region. This suggests that a mixture of two distributions is required—consistent with the abrupt change in behavior seen in Figures 1, 2.

We consider the case where *X* is a mixture of two NB distributions, equivalent to modeling Λ as a mixture of two gamma distributions. In this case, we have a mixing parameter τ, and two separate NB parametrizations (μ_1_, *r*_1_) and (μ_2_, *r*_2_). Thus,

$$\begin{array}{l} Z\sim Bernoulli(\tau )\\ X\sim \{\begin{array}{ll} NB(\mu_{1},r_{1}) & \left(Z=0\right)\\ NB(\mu_{2},r_{2}) & \left(Z=1\right) \end{array} \end{array}$$

We could find the MLE of the parameters with the EM algorithm (Dempster et al., 1977). However, since the EM algorithm can converge very slowly, we accelerated the process using SQUAREM (Varadhan and Roland, 2008). SQUAREM assumes that each step of the EM algorithm can be approximated using a particular quadratic form, allowing us to estimate the cumulation of a large number of EM updates in one go.

Note that we did not consider mixtures of Poisson distributions, since these cases are attended to by the CNM algorithm, and we did not consider mixtures of log-normal Poisson distributions due to the computational complexity.

## 4. Results

### 4.1. Model fits

We considered the following distributions:

**Table:** 

**Count distribution, *f*(*x*)**	**Parameters**	**Mixing distribution, *f*(λ)**
Poisson	1	Single point
Negative Binomial (NB)	2	Gamma
Log-normal Poisson	2	Log-normal
NB-NB mixture	5	Gamma-gamma mixture

**Figure 4:**
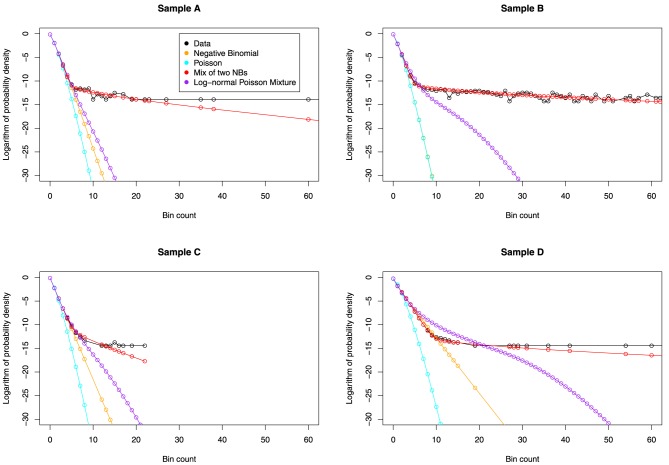
**The fit of various distributions to the count data**. We again see a striking change of behavior, occurring at around *x* = 9. The only distribution that can model this change is the NB-NB mixture.

**Figure 5:**
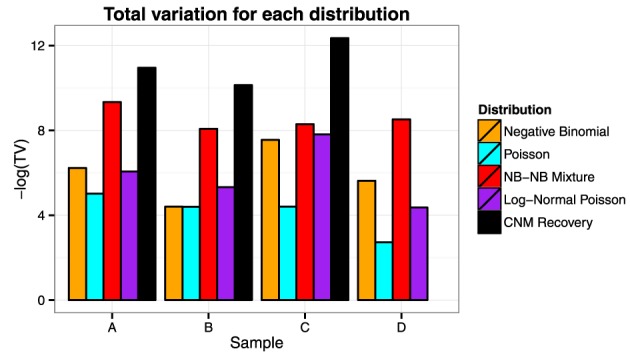
**Total variation**. We see from the plot that the NB-NB mixture outperforms all of the other distributions, achieving the closest fit to the CNM estimate in the cases where it exists.

We visually inspect the fits of the various distributions to the full data in Figure 4. To quantify the fit, we used Total Variation distance: $d_{TV}(f,g)=\frac{1}{2}\sum_{x}|f(x)-g(x)|$. The results for this are given in Figure 5.

**Figure 6:**
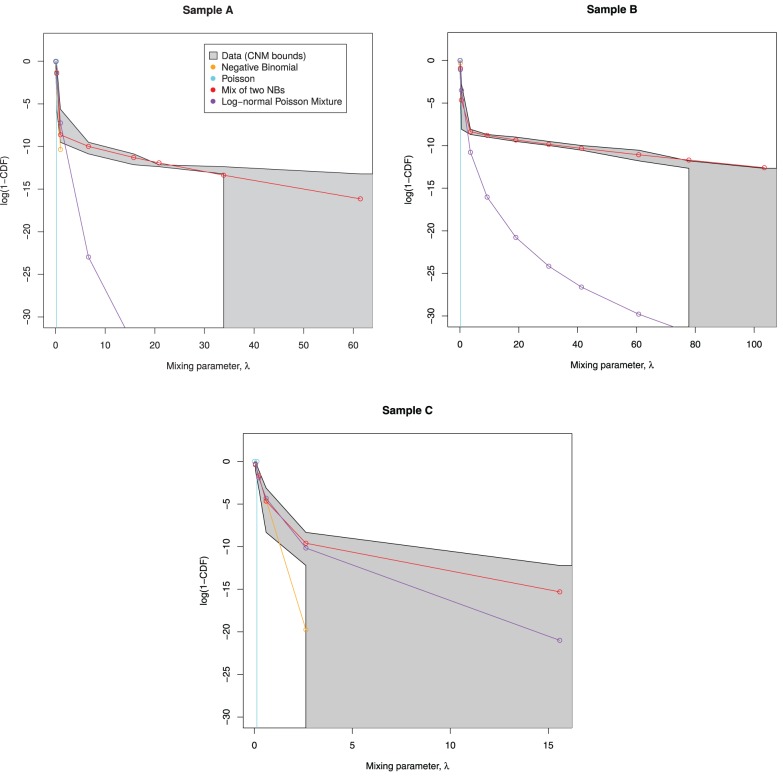
**Predicted mixing distributions, as per Figure 3, for each sample with the NB-NB mixture added**. (Sample D is omitted since CNM did not recover its distribution.) The NB-NB mixture is the only model whose mixing distribution consistently fits between the CNM bounds.

Then we examine the similarity between the mixing distributions used and the observed mixing distribution as calculated by CNM, in Figure 6.

The NB-NB mixture is the only choice of distribution that can consistently model the higher counts.

### 4.2. Zero inflation

Zero-inflated models have been proposed to improve model fit in ChIP-seq data (Rashid et al., 2011; Diaz et al., 2012). A zero-inflated model is one such that, with some probability ν, we set *X* = 0. Otherwise, we draw *X* from a candidate distribution as before.

Having accounted for some of the variation in our data with the NB-NB mixture model, we investigated whether or not zero-inflation can improve model fit further. To quantify this, we delete some proportion of zeros from the data, fit each model, then assess the fit with Total Variation as defined in Section 4.1. We also considered the case where we increase the number of zeros. Note that the log-normal Poisson fit was excluded due to computational complexity.

**Figure 7:**
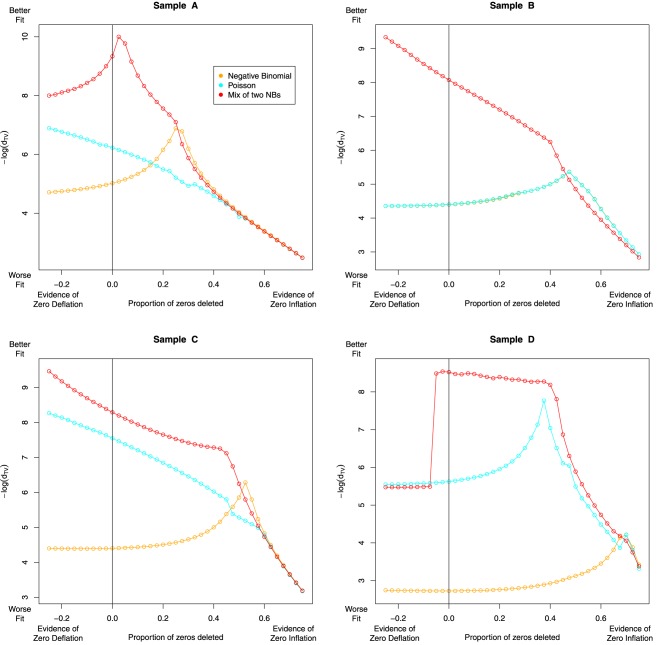
**The effect of zero removal on the model fit**. Each Subfigure represents an individual sample. A proportion of zeros are removed (or added), and after refitting each model, we reassess model fit with the Total Variation distance *d*_*TV*_, plotted as −*log*(*d_TV_*) on the *Y*-axis against zero proportion on the *X*-axis. For severely zero-inflated data, deleting zeros should improve model fit, so we expect to see a “peak” somewhere to the right of the black vertical line. The NB and Poisson models recommend the removal of most of the zero-valued data, a conclusion that seems biologically implausible. In contrast, the NB-NB mixture consistently recommends either no zero-removal, or a modest level of zero-inflation in the case of sample A. Note the dramatic change in the NB-NB mixture's fit in sample D as additional counts are added—this could be due to the accelerated EM algorithm encountering alternative local maxima.

The results are given in Figure 7. For sample A (dog) we see evidence of zero inflation, perhaps due to a less-established genome assembly. In samples B-D, the NB-NB models showed no evidence of zero-inflation. However, when using distributions that cannot account for the heavy tail in the high bin-counts, there is an erroneous indication that a zero-inflation component is necessary.

The general problem of inferring the mixture distribution whilst accounting for zero-inflation is difficult, though similar approaches exist (Lim et al., 2014; Morgan et al., 2014). We envisage an altered version of CNM that accounts for zero-inflation by adding the parameter ν defined above, with its own update step. Additional constraints may be required to make this model identifiable.

## 5. Discussion

We found that, of the distributions tested, a mixture of two NB distributions best modeled counts in input data, even after removing problematic centromeric and telomeric regions. This result, and the observation that the empirical distribution changes behavior at high counts, reflected two apparent sources of counts in the data—one large population of counts, and another smaller population of higher counts. The regions with higher counts have higher variance than lower counts, and could be mistaken for peaks if using a naïve peak-calling method.

Importantly, we saw that the heavy tail of large counts could cause inadequate models to suggest the need for a zero-inflation component. Our results suggest that researchers should check that large counts are not distorting model fit before assuming that a zero-inflation component is necessary.

Several aspects of experimental design could influence the abundance of large counts. Any biases in mapping that cause reads to align preferentially to the same locus could cause high-count artifacts. Thus, we might be able to reduce the abundance of large counts by improving mappability (that is, reducing the number of ambiguously-mapped reads). For example, better genome assembly, or use of a better aligner, or using longer reads could all accomplish this task.

Appropriate mixture modeling could apply when simulating ChIP-seq data—a simulation paradigm that fails to account for these different populations of counts cannot test peak-callers properly and overestimates their performance (Zhang et al., 2008b). A better understanding of the properties of the underlying mixture model allows us to simulate noise in ChIP-seq data sets more accurately.

Mixture modeling is also of importance when peak-calling in ChIP-seq data, allowing us to model large counts without removing them. Models that regress on genomic covariates, such as copy number or GC content, can help us explain some of the variation in the data (Rashid et al., 2011; Robinson et al., 2012). However, our unsupervised approach can model the noise, even in the absence of appropriate covariates to regress over—for example, if we have samples that are from less well-elucidated species or that have abnormal copy number events (such as after chromothrypsis). We may also be able to extend the approach to make inferences about the variation that remains after regressing out known covariates. Additionally, we note that Rashid et al. (2011) assume constant dispersion due to general linear modeling restrictions—in contrast, a mixture model permits multiple dispersions.

Alternatively, should we wish to adopt a blacklist strategy, we can construct a blacklist *de novo* by classifying bins into artifact and non-artifact regions (for example, by finding the probability that a bin belongs to each of the NB components in our mixture model). Again, this could be particularly useful for abnormal samples or species.

An alternative approach to smoothing the MLE $\hat{f}$(λ) is to enforce continuity of $\hat{f}$(λ) during maximum likelihood estimation. For example, Liu et al. (2009) minimize a penalized log-likelihood:

$$\ell_{p}(f)=\ell (f)-\alpha \int_{\lambda_{0}}^{\lambda_{1}}{\left[f^{\prime \prime }(\lambda )\right]}^{2}d\lambda$$

where α, the smoothing parameter, controls how smooth the output function is required to be.

The methods we described have general applicability to other sequencing experiments based on genomic DNA, and not just ChIP-seq. As we increasingly see the emergence of large-scale epigenetic studies based on gDNA assays like ChIP-seq, it is important to choose models whose false discovery rates are robust. Properly accounting for the null variability in ChIP-seq data is vital to avoid false positives.

## Funding

We acknowledge the support of the University of Cambridge, the Medical Research Council, Cancer Research UK, and Hutchison-Whampoa.

### Conflict of interest statement

The authors declare that the research was conducted in the absence of any commercial or financial relationships that could be construed as a potential conflict of interest.

## References

[B1] Boes D. C. (1966). On the estimation of mixing distributions. Ann. Math. Stat. 37, 177–188 10.1214/aoms/1177699607

[B2] Cairns J. Lynch A. G. Tavaré S. (2013). Statistical aspects of ChIP-seq analysis, in Advances in Statistical Bioinformatics, Chapter 7, eds Do K.-A. Qin Z. S. Vannucci M. (New York, NY: Cambridge University Press), 138–169 10.1017/CBO9781139226448.008

[B3] Cairns J. Spyrou C. Stark R. Smith M. L. Lynch A. G. Tavaré S. (2011). BayesPeak - an R package for analysing ChIP-seq data. Bioinformatics 27, 713–714. 10.1093/bioinformatics/btq685 21245054 PMC3042177

[B4] Carroll T. S. Liang Z. Salama R. Stark R. de Santiago I. (2014). Impact of artifact removal on ChIP quality metrics in ChIP-seq and ChIP-exo data. Front. Genet. 5:75. 10.3389/fgene.2014.00075 24782889 PMC3989762

[B5] Dempster A. Laird N. Rubin D. (1977). Maximum likelihood from incomplete data via the EM algorithm. J. Roy. Stat. Soc. B 39, 1–38. 24782889

[B6] Diaz A. Park K. Lim D. A. Song J. S. (2012). Normalization, bias correction, and peak calling for ChIP-seq. Stat. Appl. Genet. Molec. Biol. 11:9. 10.1515/1544-6115.1750 22499706 PMC3342857

[B7] Laird N. (1978). Nonparametric maximum likelihood estimation of a mixing distribution. J. Am. Stat. Assoc. 73, 805 10.1080/01621459.1978.10480103

[B8] Landt S. G. Marinov G. K. Kundaje A. Kheradpour P. Pauli F. Batzoglou S.. (2012). ChIP-seq guidelines and practices of the ENCODE and modENCODE consortia. Genome Res. 22, 1813–1831. 10.1101/gr.136184.111 22955991 PMC3431496

[B9] Lim H. K. Li W. K. Yu P. L. (2014). Zero-inflated Poisson regression mixture model. Comput. Stat. Data Anal. 71, 151–158 10.1016/j.csda.2013.06.021

[B10] Liu L. Levine M. Zhu Y. (2009). A functional EM algorithm for mixing density estimation via nonparametric penalized likelihood maximization. J. Comp. Graph. Stat. 18, 481–504 10.1198/jcgs.2009.07111

[B11] Lunn D. J. Thomas A. Best N. Spiegelhalter D. (2000). WinBUGS - A Bayesian modelling framework: concepts, structure, and extensibility. Stat. Comput. 10, 325–337 10.1023/A:1008929526011

[B12] Marioni J. C. Mason C. E. Mane S. M. Stephens M. Gilad Y. (2008). RNA-seq: an assessment of technical reproducibility and comparison with gene expression arrays. Genome Res. 18, 1509–1517. 10.1101/gr.079558.108 18550803 PMC2527709

[B13] Morgan C. J. Lenzenweger M. F. Rubin D. B. Levy D. L. (2014). A hierarchical finite mixture model that accommodates zero-inflated counts, non-independence, and heterogeneity. Stat. Med. 33, 2238–2250. 10.1002/sim.6091 24443287 PMC4057921

[B14] Myers R. M. Stamatoyannopoulos J. Snyder M. Dunham I. Hardison R. C. Bernstein B. E.. (2011). A user's guide to the Encyclopedia Of DNA Elements (ENCODE). PLoS Biol. 9:e1001046. 10.1371/journal.pbio.1001046 21526222 PMC3079585

[B15] Park P. J. (2009). ChIP-seq: advantages and challenges of a maturing technology. Nat. Rev. Genet. 10, 669–680. 10.1038/nrg2641 19736561 PMC3191340

[B16] Rashid N. U. Giresi P. G. Ibrahim J. G. Sun W. Lieb J. D. (2011). ZINBA integrates local covariates with DNA-seq data to identify broad and narrow regions of enrichment, even within amplified genomic regions. Genome Biol. 12:R67. 10.1186/gb-2011-12-7-r67 21787385 PMC3218829

[B17] Robinson M. D. Strbenac D. Stirzaker C. Statham A. L. Song J. Speed T. P.. (2012). Copy-number-aware differential analysis of quantitative DNA sequencing data. Genome Res. 22, 2489–2496. 10.1101/gr.139055.112 22879430 PMC3514678

[B18] Ross-Innes C. S. Stark R. Teschendorff A. E. Holmes K. A. Ali H. R. Dunning M. J.. (2012). Differential oestrogen receptor binding is associated with clinical outcome in breast cancer. Nature 481, 389–393. 10.1038/nature10730 22217937 PMC3272464

[B19] Roueff F. Rydén T. (2005). Nonparametric estimation of mixing densities for discrete distributions. Ann. Stat. 33, 2066–2108 10.1214/009053605000000381

[B20] Saha K. Paul S. (2005). Bias-corrected maximum likelihood estimator of the negative binomial dispersion parameter. Biometrics 61, 179–185. 10.1111/j.0006-341X.2005.030833.x 15737091

[B21] Schmidt D. Wilson M. D. Ballester B. Schwalie P. C. Brown G. D. Marshall A.. (2010). Five-vertebrate ChIP-seq reveals the evolutionary dynamics of transcription factor binding. Science 328, 1036–1040. 10.1126/science.1186176 20378774 PMC3008766

[B22] Simar L. (1976). Maximum likelihood estimation of a compound Poisson process. Ann. Stat. 4, 1200–1209. 10.1214/aos/117634365116024052

[B23] Song Q. Smith A. D. (2011). Identifying dispersed epigenomic domains from ChIP-Seq data. Bioinformatics 27, 870–871. 10.1093/bioinformatics/btr030 21325299 PMC3051331

[B24] Spyrou C. Stark R. Lynch A. G. Tavaré S. (2009). BayesPeak: bayesian analysis of ChIP-seq data. BMC Bioinform. 10:299. 10.1186/1471-2105-10-299 19772557 PMC2760534

[B25] Thygesen H. H. Zwinderman A. H. (2006). Modeling SAGE data with a truncated gamma-Poisson model. BMC Bioinform. 7:157. 10.1186/1471-2105-7-157 16549008 PMC1479844

[B26] Tucker H. (1963). An estimate of the compounding distribution of a compound Poisson distribution. Theor. Probab. Appl. 8:195 10.1137/1108021

[B27] Varadhan R. Roland C. (2008). Simple and globally convergent methods for accelerating the convergence of any EM algorithm. Scand. J. Stat. 35, 335–353 10.1111/j.1467-9469.2007.00585.x

[B28] Venables W. N. Ripley B. D. (2002). Modern Applied Statistics with S, 4th Edn. New York, NY: Springer 10.1007/978-0-387-21706-2

[B29] Wang Y. (2007). On fast computation of the non-parametric maximum likelihood estimate of a mixing distribution. J. Roy. Stat. Soc. B 69, 185–198 10.1111/j.1467-9868.2007.00583.x

[B30] Wu S. Wang J. Zhao W. Pounds S. Cheng C. (2010). ChIP-PaM: an algorithm to identify protein-DNA interaction using ChIP-Seq data. Theor. Biol. Med. Model. 7:18. 10.1186/1742-4682-7-18 20525272 PMC2893127

[B31] Zhang Y. Liu T. Meyer C. A. Eeckhoute J. Johnson D. S. Bernstein B. E.. (2008a). Model-based analysis of ChIP-Seq (MACS). Genome Biol. 9, R137. 10.1186/gb-2008-9-9-r137 18798982 PMC2592715

[B31a] Zhang Z. D. Rozowsky J. Snyder M. Chang J. Gerstein M. (2008b). Modeling ChIP sequencing *in silico* with applications. PLoS Comp. Biol. 4:e1000158. 10.1371/journal.pcbi.1000158 18725927 PMC2507756

