# Supplementary material for: Quantifying the impact of inter-site heterogeneity on the distribution of ChIP-seq data
Source: Front Genet. 2014 Nov 14;5:399. doi: 10.3389/fgene.2014.00399 (PMC4231950; doi:10.3389/fgene.2014.00399)
Supplement: Supplementary file 1 [file DataSheet1.ZIP › README.pdf]

Quantifying the impact of inter-site heterogeneity on the distribution of ChIP-seq data - Supplemental Data

Jonathan Cairns, Andy Lynch, Simon Tavaré

(For details on how to compile this R Markdown document, see http://rmarkdown.rstudio.com.)

IMPORTANT: To re-run the CNM analysis, you will need some libraries:

- Ensure that you have installed all of the required libraries, named at the beginning of the following file (you can also enable the option to use multicore parallel processing):

```
source("01LibrariesFunctions.R")
```

- Ensure that you have installed the LSEI package, available from: https://www.stat.auckland.ac.nz/~yongwang/.
- We have included a copy of *cnm.R*, but a newer version may be available from the same location.

(Note the custom parameters used for CNM, namely $check = 1000$ and $tol = 10^{-3}$)

Workflow

The *DataCountFreq/* directory contains count tables, such as the following:

```
read.csv("DataCountFreq/1Cfam.csv")
```

```
##     X  x   freq
## 1   0  0 933301
## 2   1  1 149737
## 3   2  2  15484
## 4   3  3   1275
## 5   4  4    115
## 6   5  5     24
## 7   6  6      8
## 8   7  7     10
## 9   8  8      8
## 10  9  9     10
## 11 10 10      1
## 12 11 11      3
## 13 12 12      2
## 14 13 13      1
## 15 14 14      2
## 16 15 15      4
## 17 17 17      3
## 18 19 19      1
## 19 20 20      1
## 20 22 22      1
## 21 23 23      1
## 22 27 27      1
```

```
## 23 35 35       1
## 24 38 38       1
## 25 60 60       1
## 26 63 63       1
```

Optional: If you wish to regenerate these count tables from the original data:

- Download the .bed files – provided in SupplementalDataBedFiles.tar.gz.
- Extract the *Data/* and *DataChIP/* directories.
- Uncomment the following lines in README.Rmd. The tables will be regenerated next time this file is knitted:

```
#source("02ProduceCountFreqs.R")
#source("03ProduceCountFreqsChIP.R")
#source("04ProduceCountFreqsNoDups.R")
```

Information on which samples are present is found in the *Targets.csv* file:

```
read.csv("Targets.csv")
```

```
##     sample                  name             file     mu precision
## 1        A                  Cfam         1Cfam.csv -1.963    2.7180
## 2        B                  Mmus          2Mmu.csv -2.331    1.2960
## 3        C                  MCF7         3MCF7.csv -2.399    1.3790
## 4        D        Primary Tumour      4BT82277.csv -1.708    0.9708
## 5   A-ChIP             Cfam ChIP     1CfamChIP.csv     NA        NA
## 6   B-ChIP             Mmus ChIP      2MmuChIP.csv     NA        NA
## 7   C-ChIP             MCF7 ChIP     3MCF7ChIP.csv     NA        NA
## 8   D-ChIP   Primary Tumour ChIP  4BT82277ChIP.csv     NA        NA
## 9  A-NoDup            Cfam Nodup    1CfamNodup.csv     NA        NA
## 10 B-NoDup            Mmus Nodup     2MmuNodup.csv     NA        NA
## 11 C-NoDup            MCF7 Nodup    3MCF7Nodup.csv     NA        NA
## 12 D-NoDup Primary Tumour Nodup 4BT82277Nodup.csv     NA        NA
```

Note that, if you are using your own data, the entries in the "file" column must match .csv file names in the *DataCountFreq/* directory. However, there is no need to supply the raw .bed files.

To run the full analysis, use the following:

```
#source("analysis.R")
```

This script generates plots in the folder *plots/*.

Example plots - Sample A

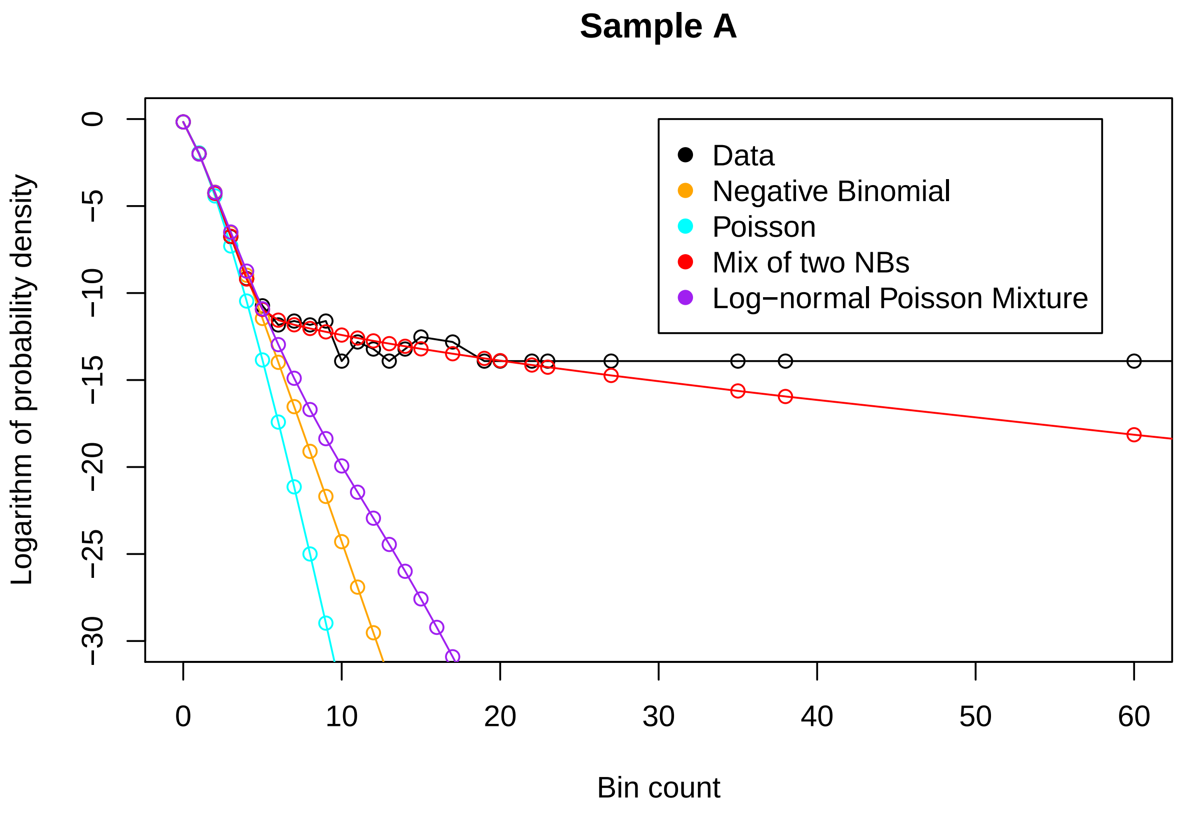

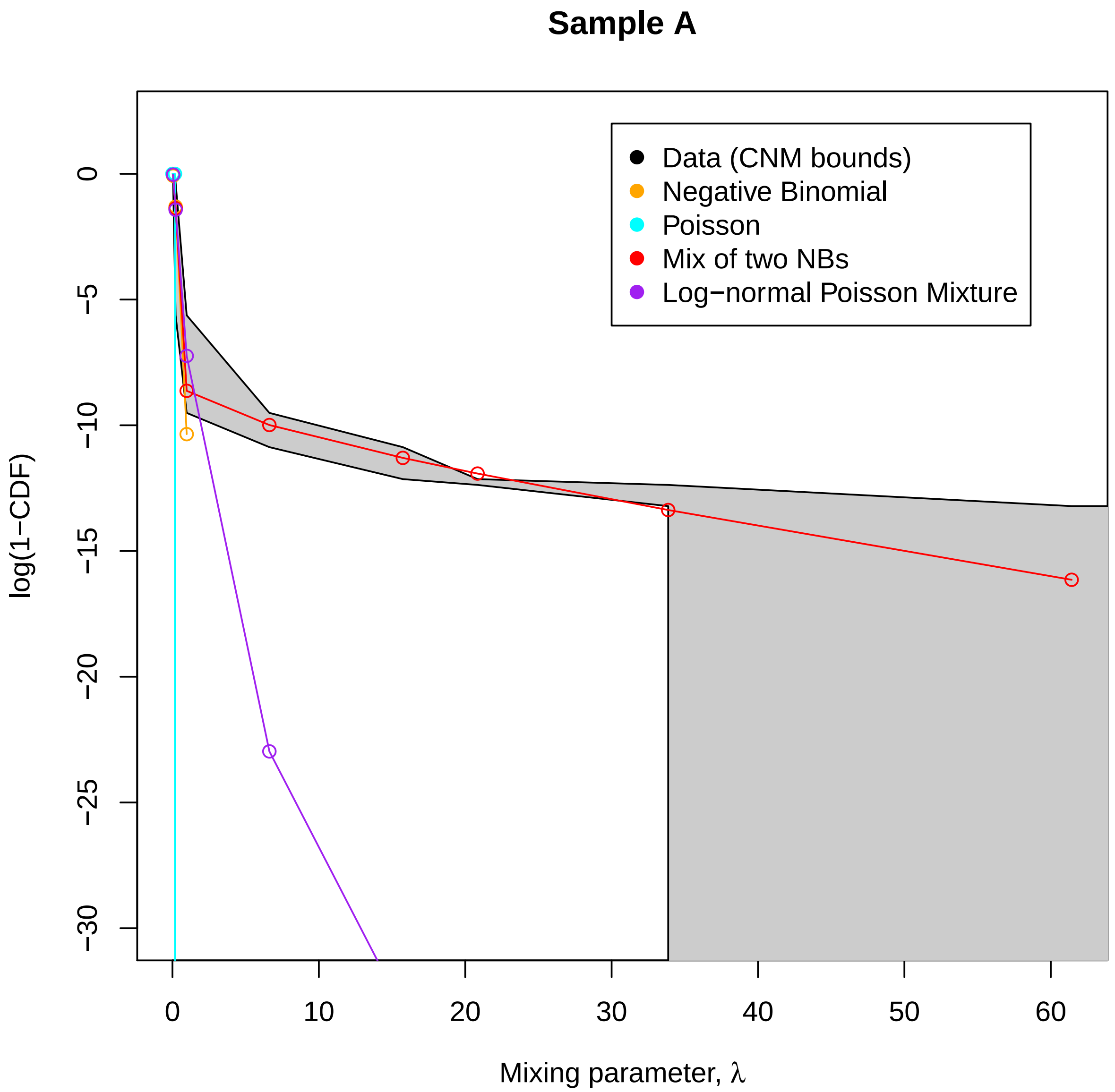
Sample A
Data (CNM bounds)
Negative Binomial
Poisson
Mix of two NBs
Log−normal Poisson Mixture
log(1−CDF)
Mixing parameter, λ
0
−5
−10
−15
−20
−25
−30
0
10
20
30
40
50
60

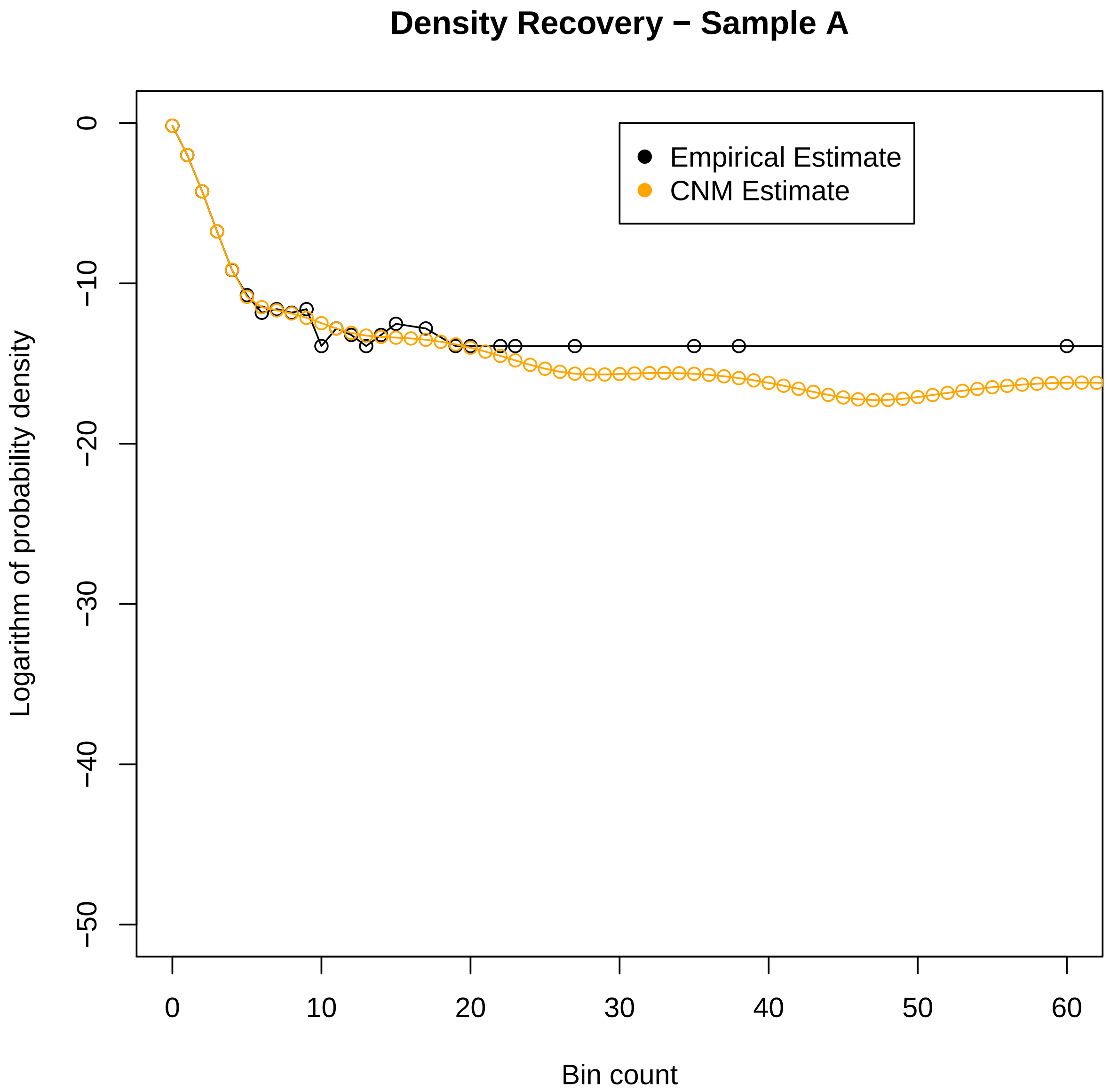
Density Recovery − Sample A
Empirical Estimate
CNM Estimate
Logarithm of probability density
Bin count
0
−10
−20
−30
−40
−50
0
10
20
30
40
50
60

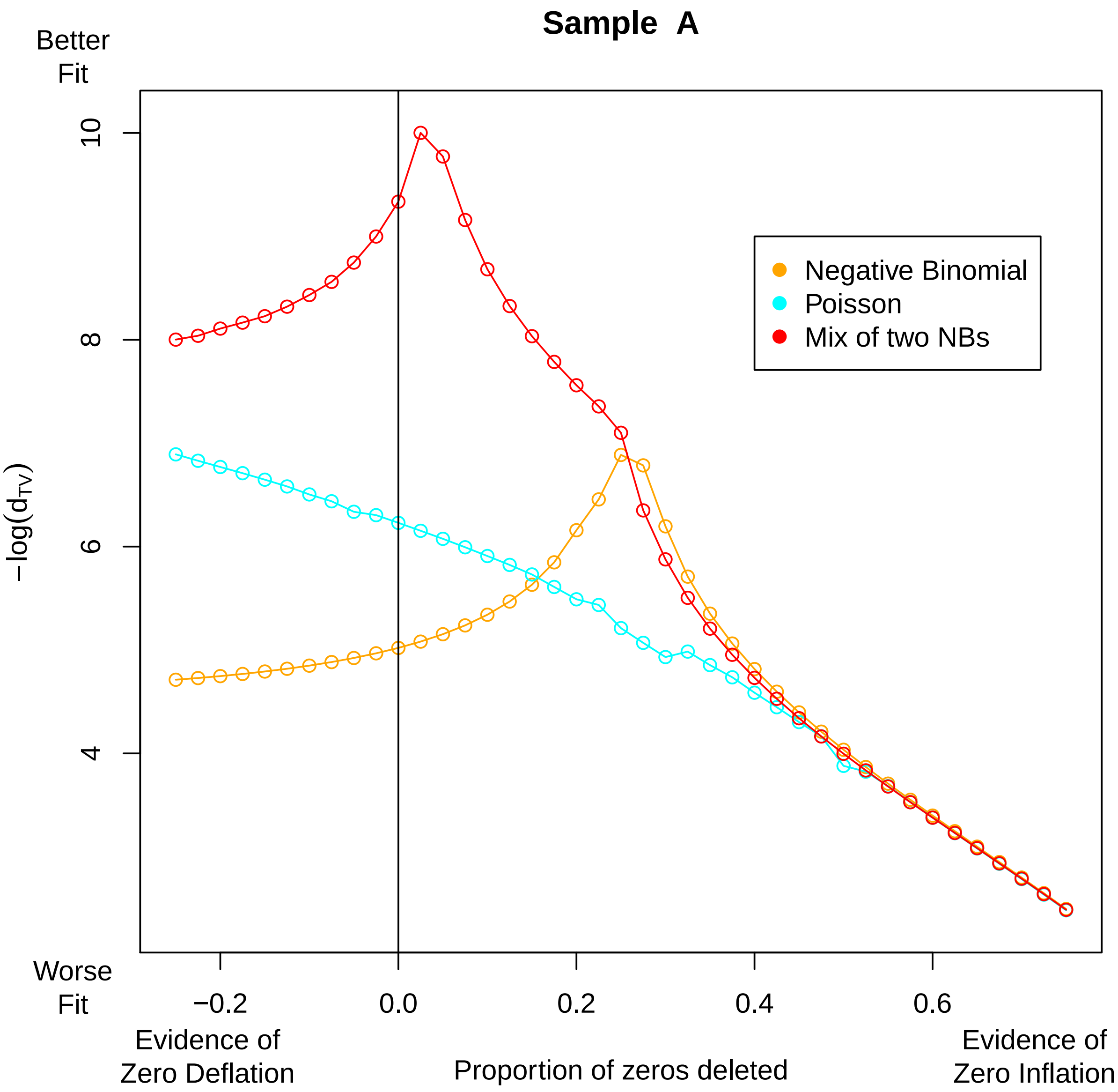
Sample A
Better
Fit
Worse
Fit
−log(d_{TV})
10
8
6
4
−0.2
0.0
0.2
0.4
0.6
Evidence of
Zero Deflation
Proportion of zeros deleted
Evidence of
Zero Inflation
Negative Binomial
Poisson
Mix of two NBs

Example plots - Sample B

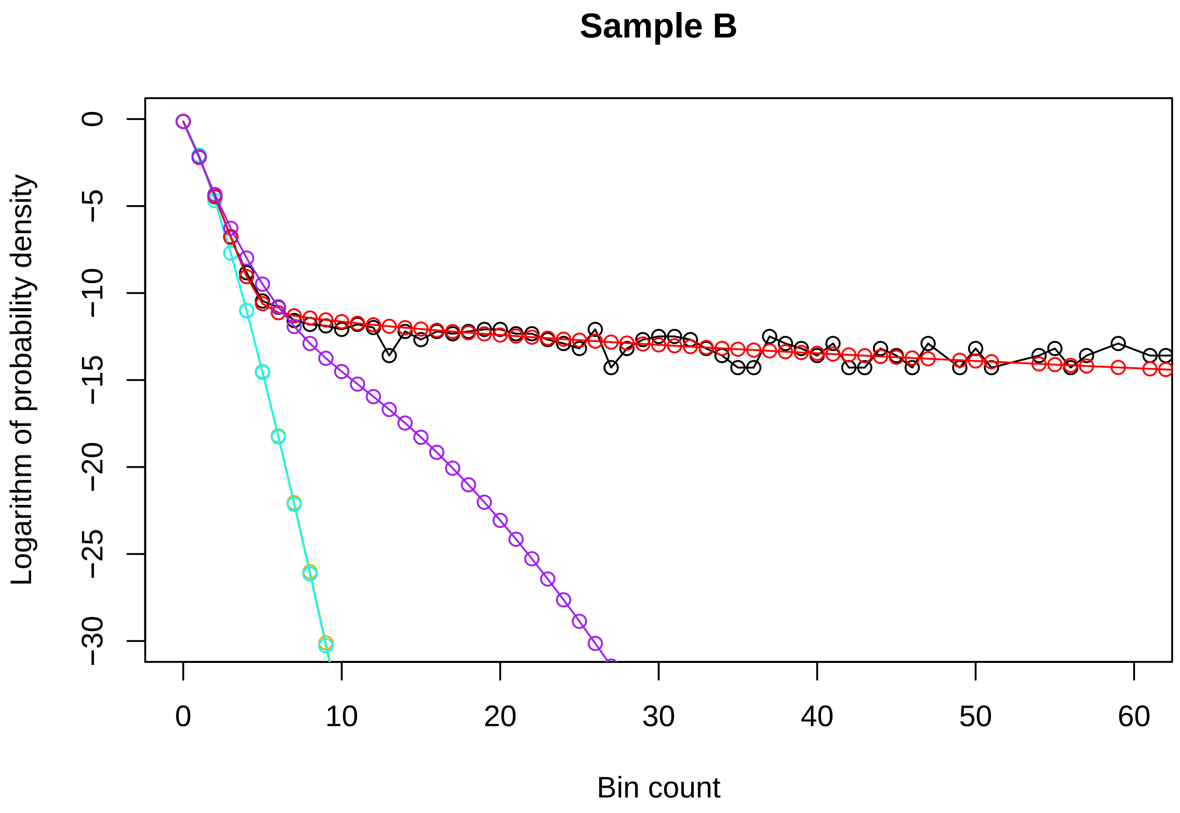

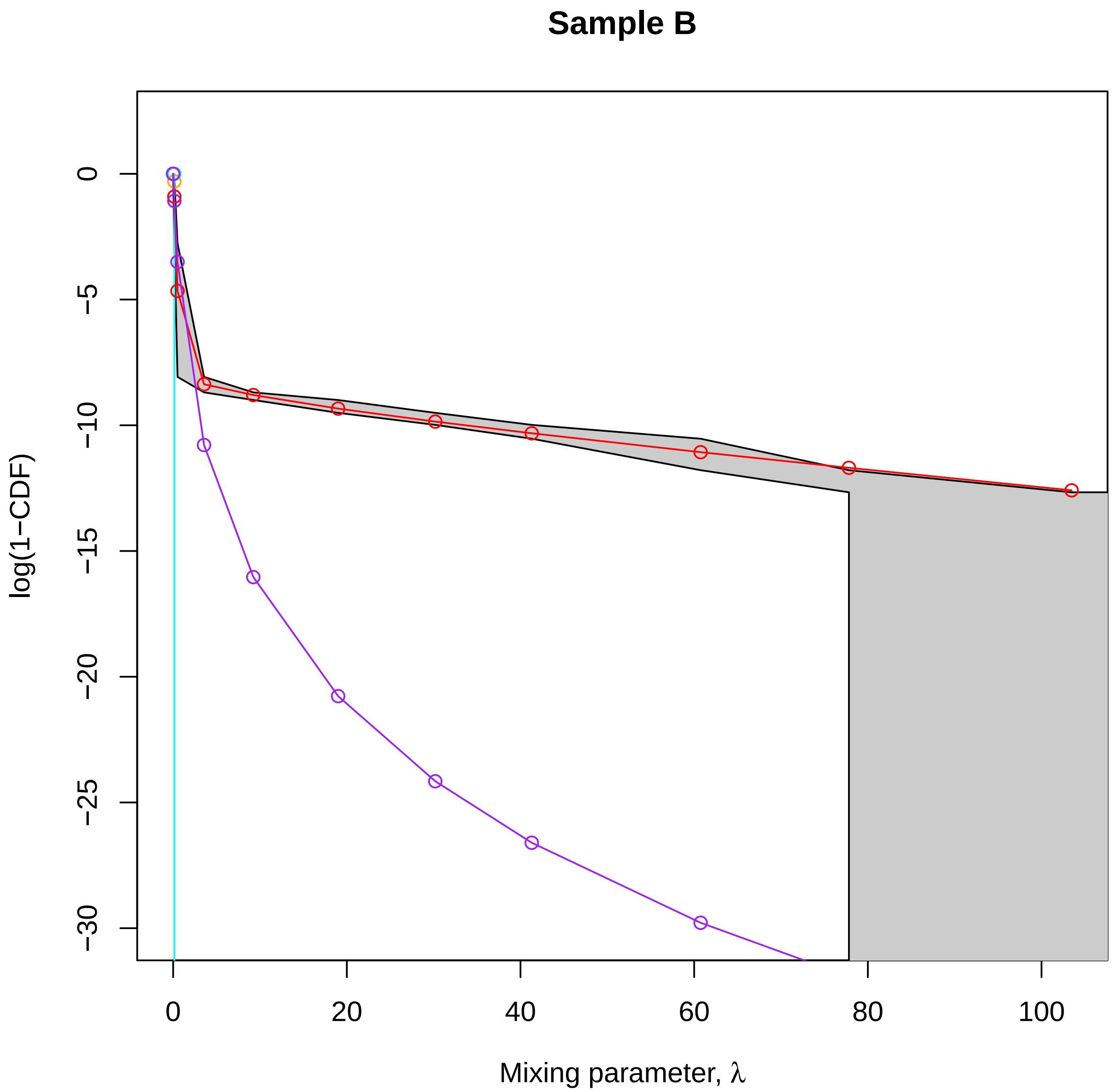
Sample B
log(1−CDF)
0
−5
−10
−15
−20
−25
−30
0
20
40
60
80
100
Mixing parameter, λ

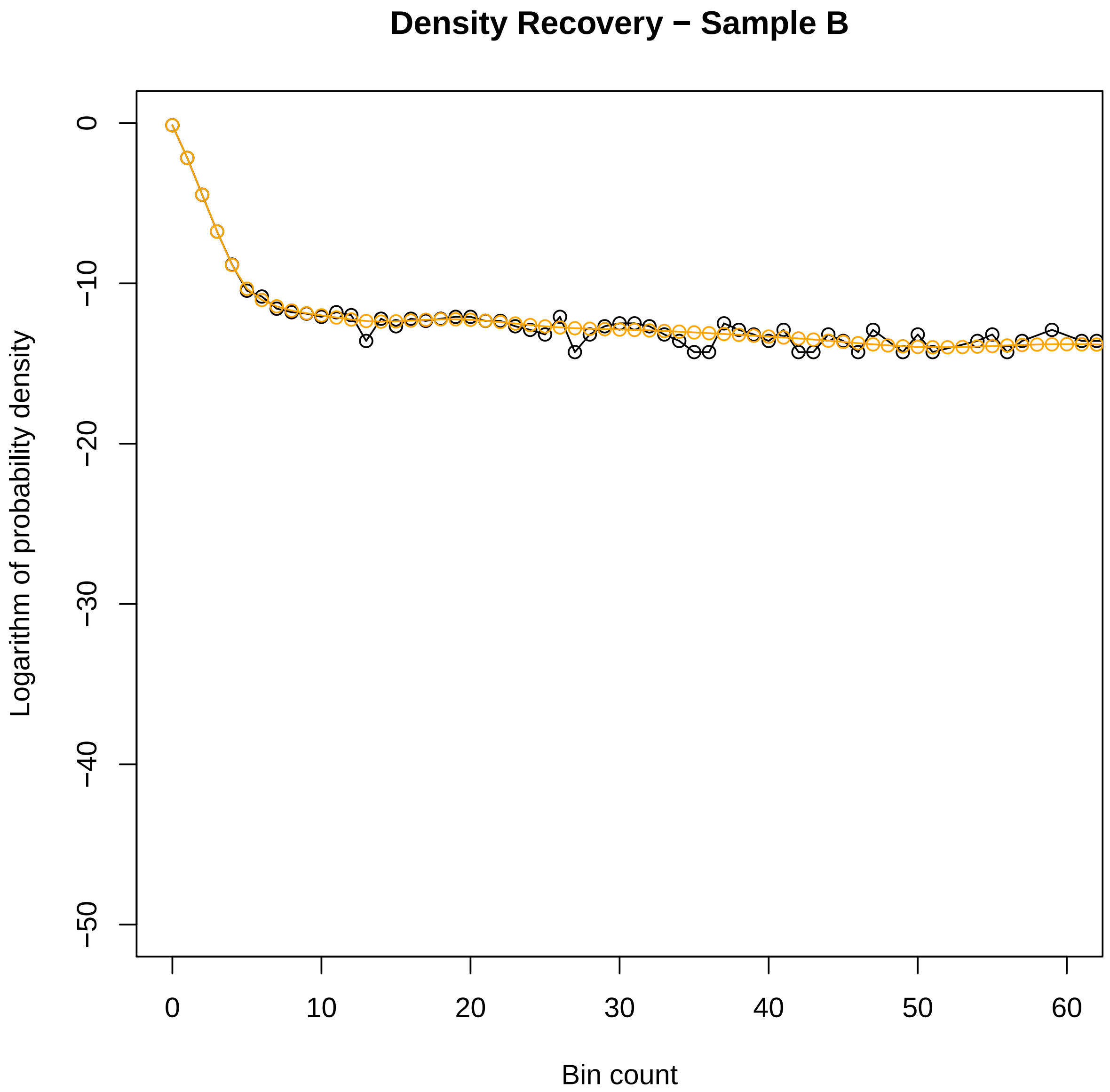

Density Recovery − Sample B
Logarithm of probability density
Bin count
0
−10
−20
−30
−40
−50
0
10
20
30
40
50
60

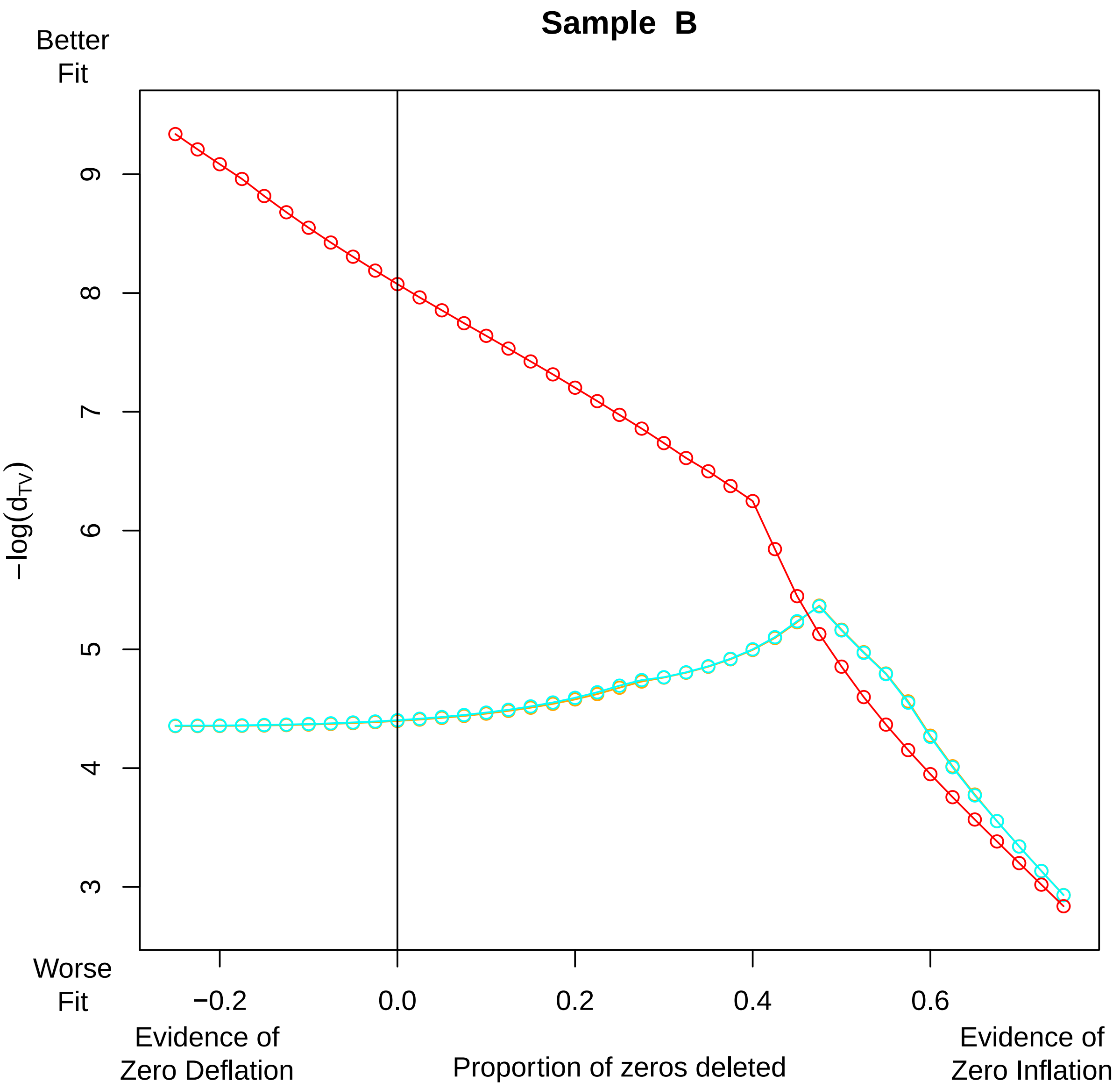
Sample B
Better Fit
Worse Fit
−log(dTV)
9
8
7
6
5
4
3
−0.2
0.0
0.2
0.4
0.6
Evidence of Zero Deflation
Proportion of zeros deleted
Evidence of Zero Inflation

Example plots - Sample C

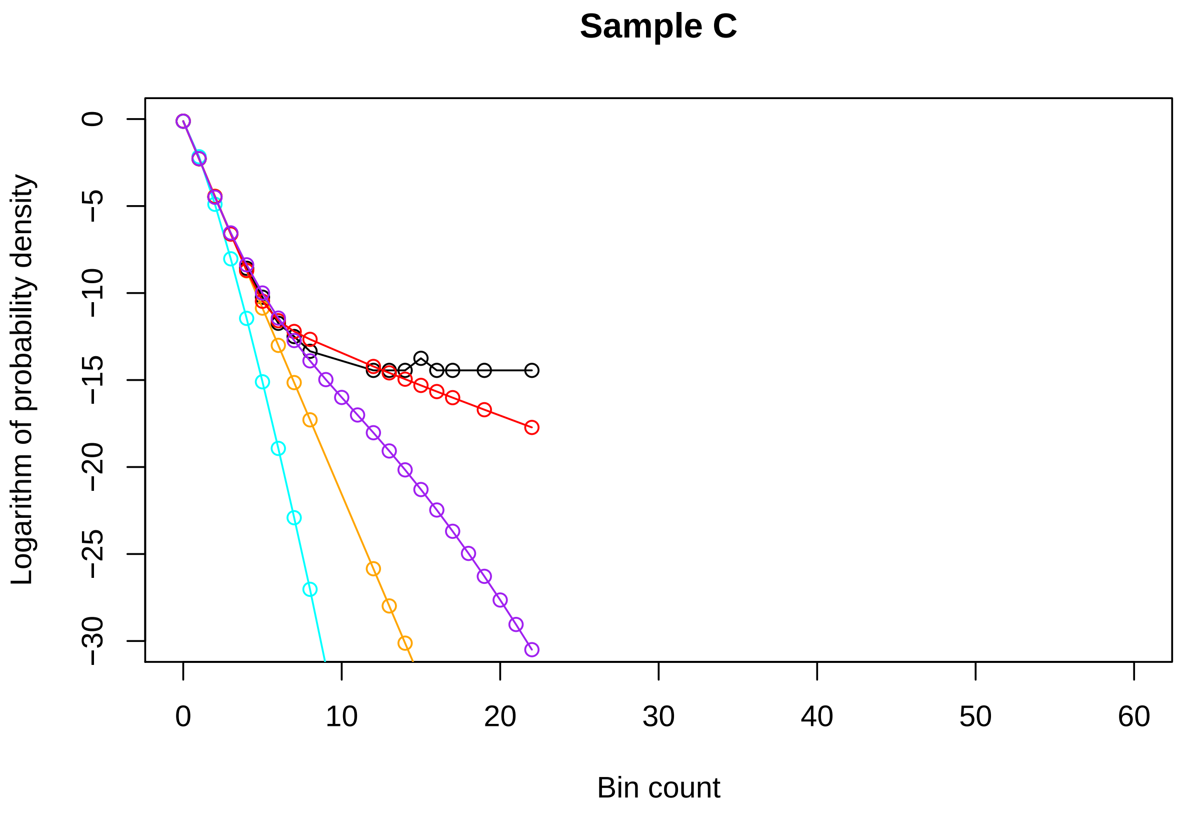

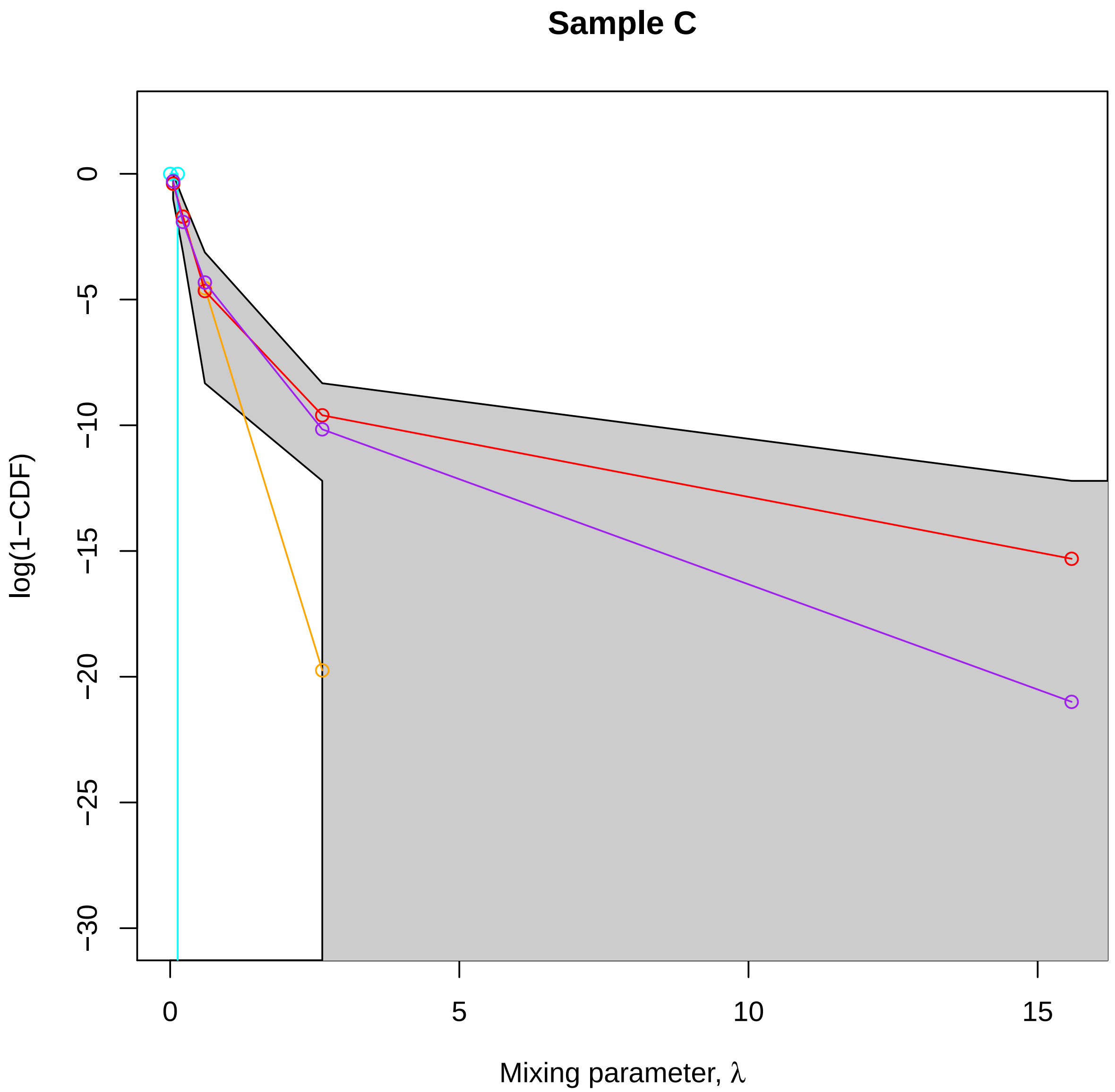
Sample C
log(1−CDF)
0
−5
−10
−15
−20
−25
−30
0
5
10
15
Mixing parameter, λ

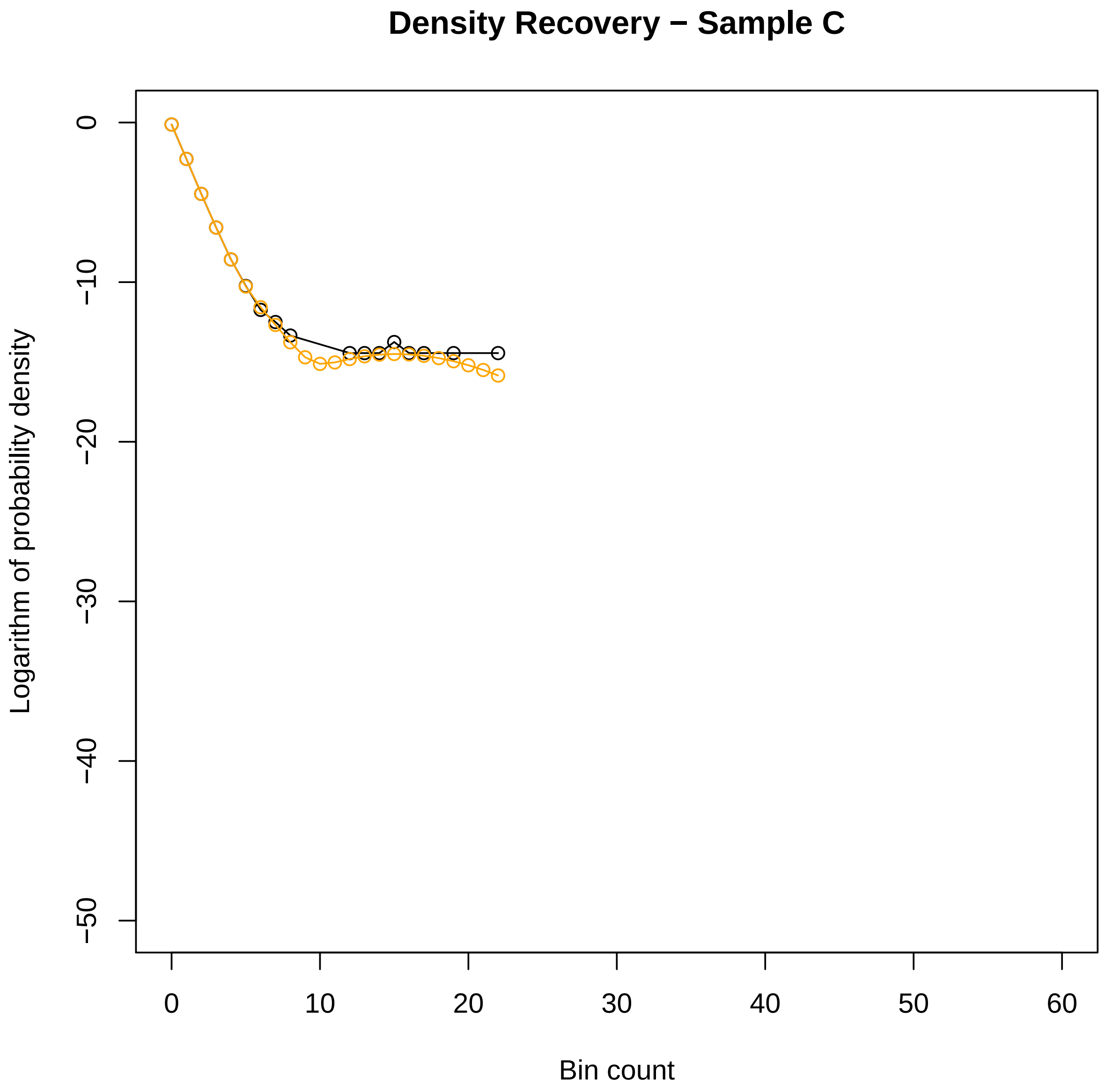
Density Recovery − Sample C
Logarithm of probability density
Bin count
0
−10
−20
−30
−40
−50
0
10
20
30
40
50
60

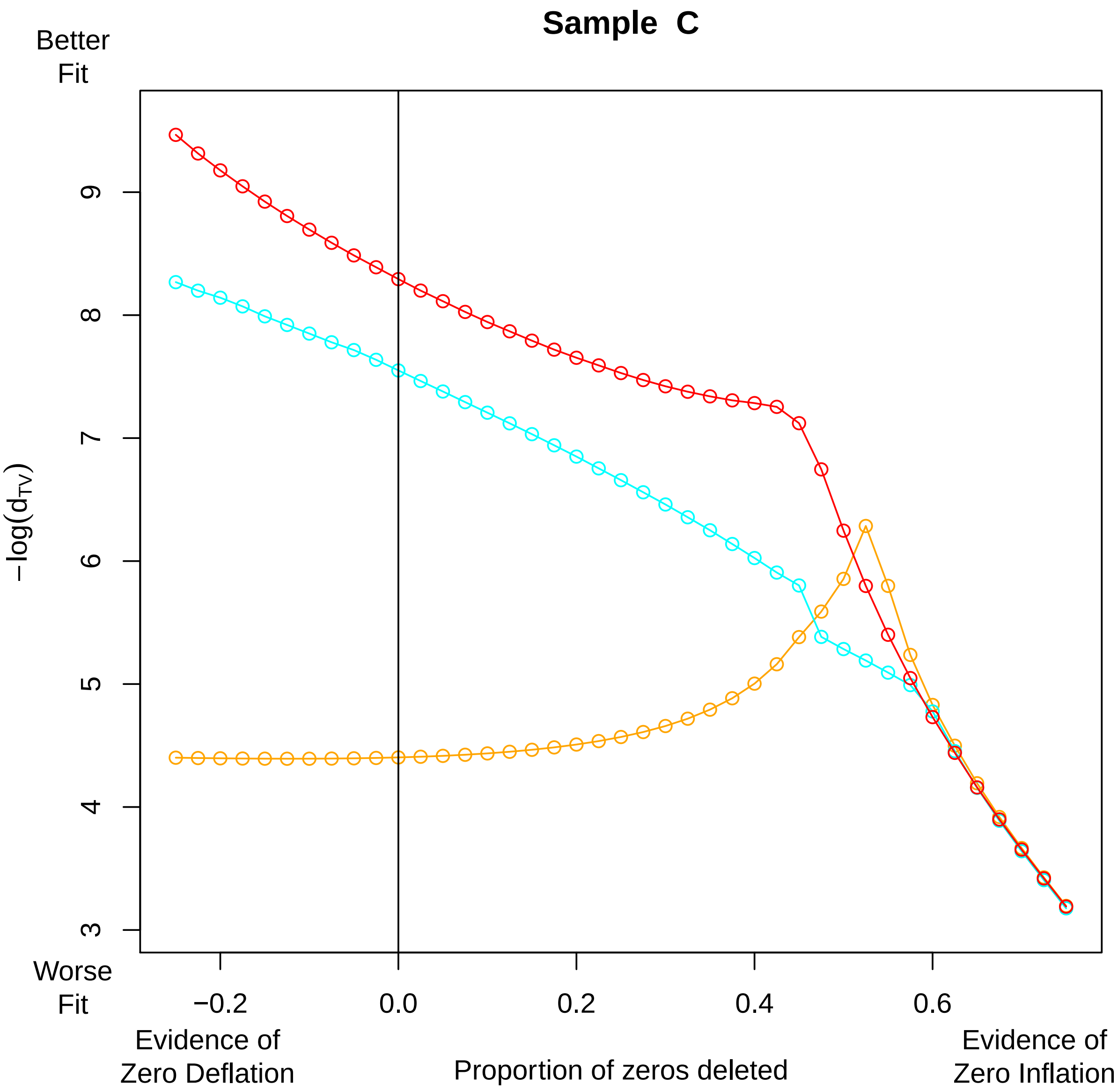
Sample C
Better
Fit
Worse
Fit
−log(d_TV)
9
8
7
6
5
4
3
−0.2
0.0
0.2
0.4
0.6
Evidence of
Zero Deflation
Proportion of zeros deleted
Evidence of
Zero Inflation

Example plots - Sample D

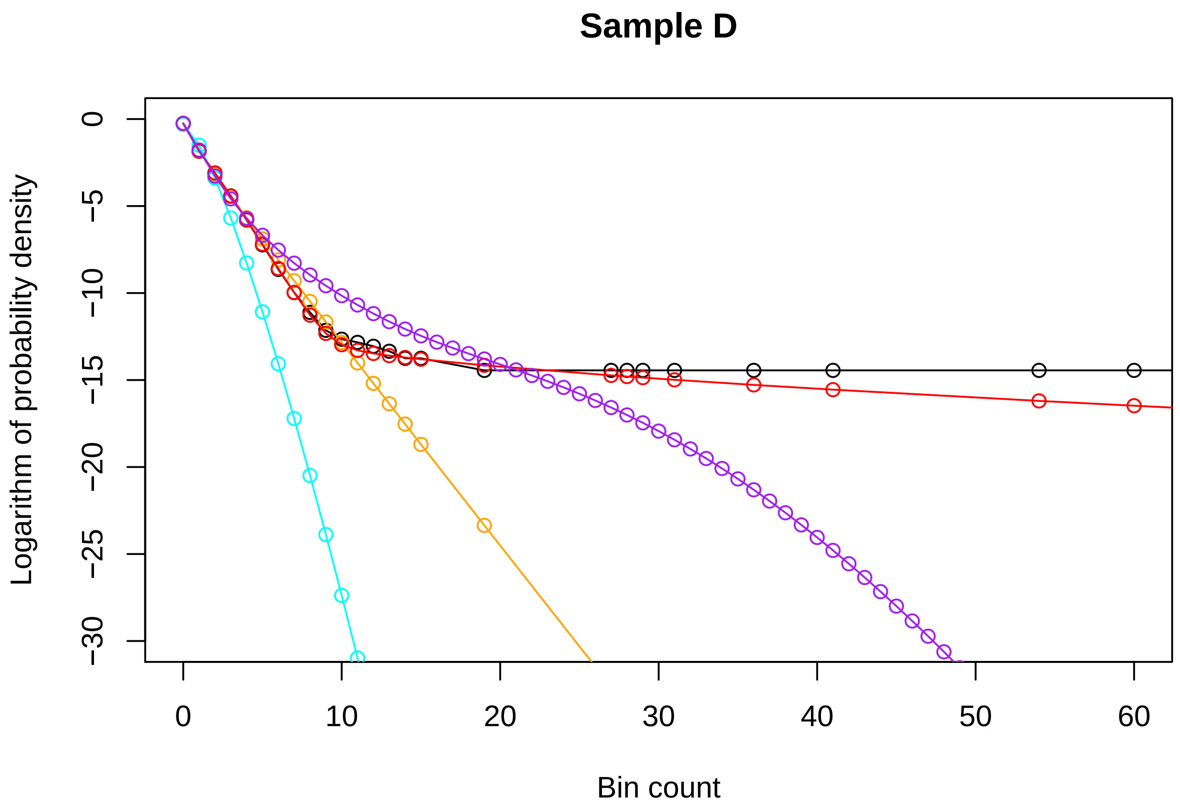

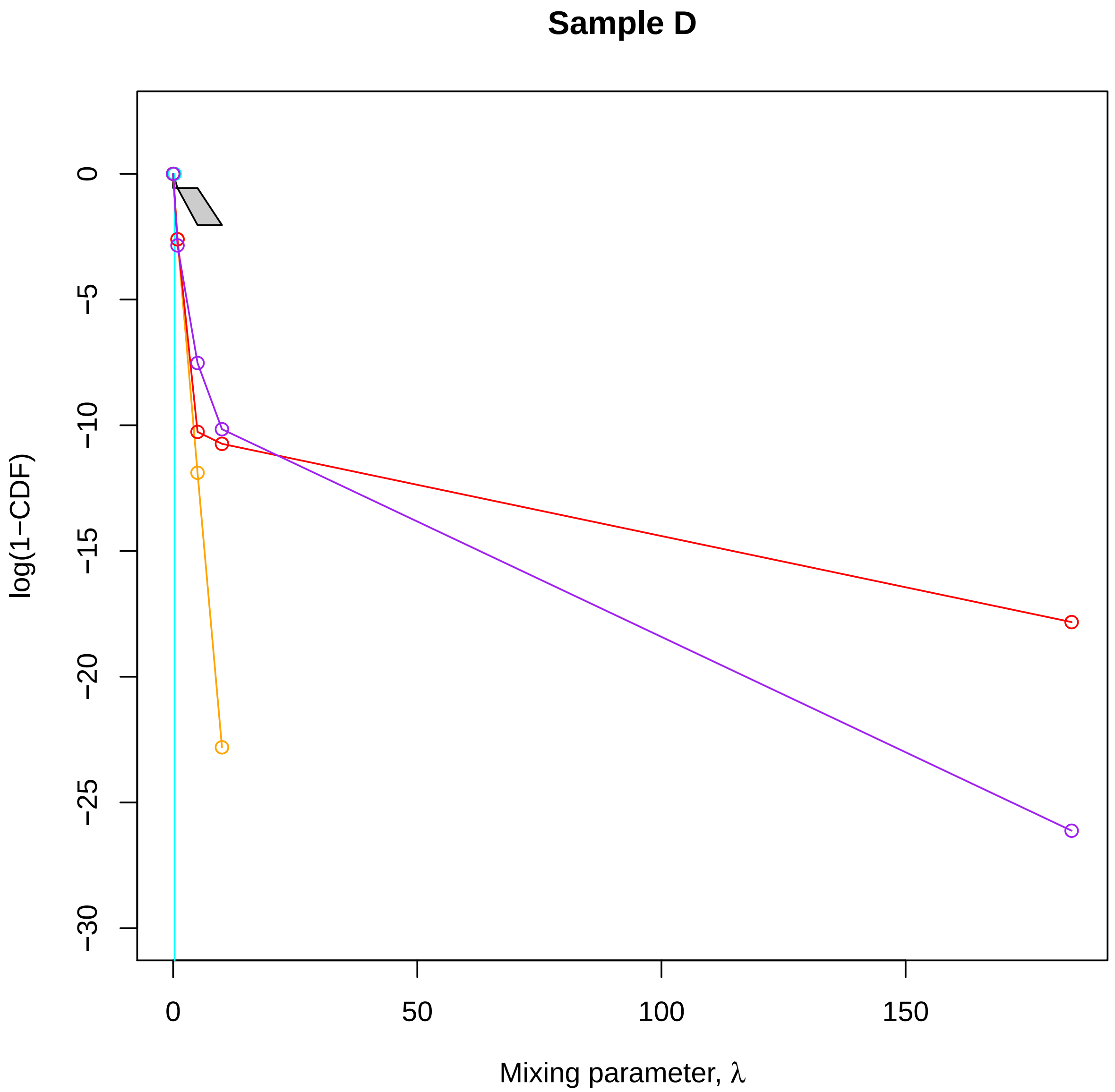
Sample D
log(1−CDF)
0
−5
−10
−15
−20
−25
−30
0
50
100
150
Mixing parameter, λ

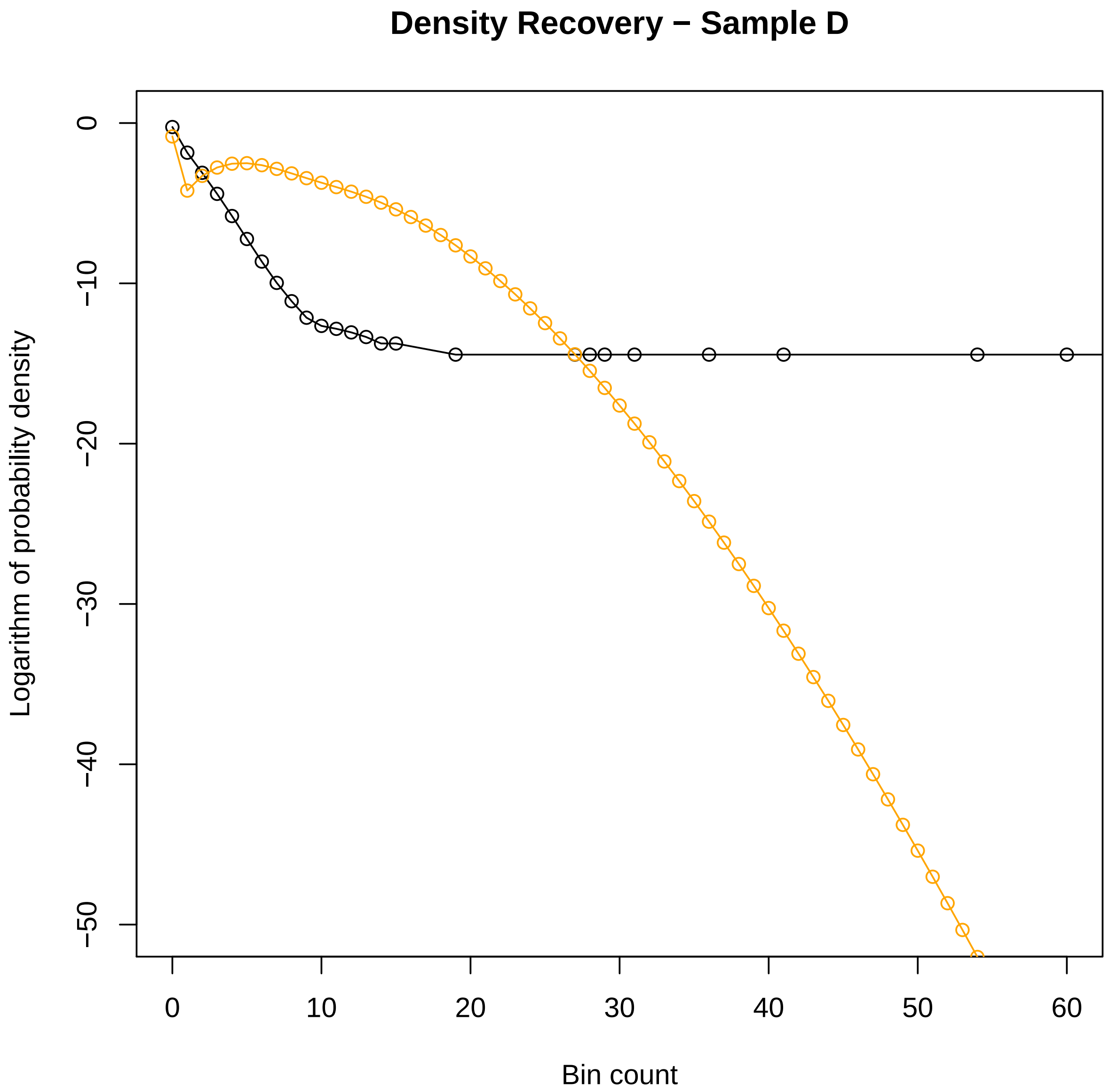

Density Recovery − Sample D
Logarithm of probability density
0
−10
−20
−30
−40
−50
0
10
20
30
40
50
60
Bin count

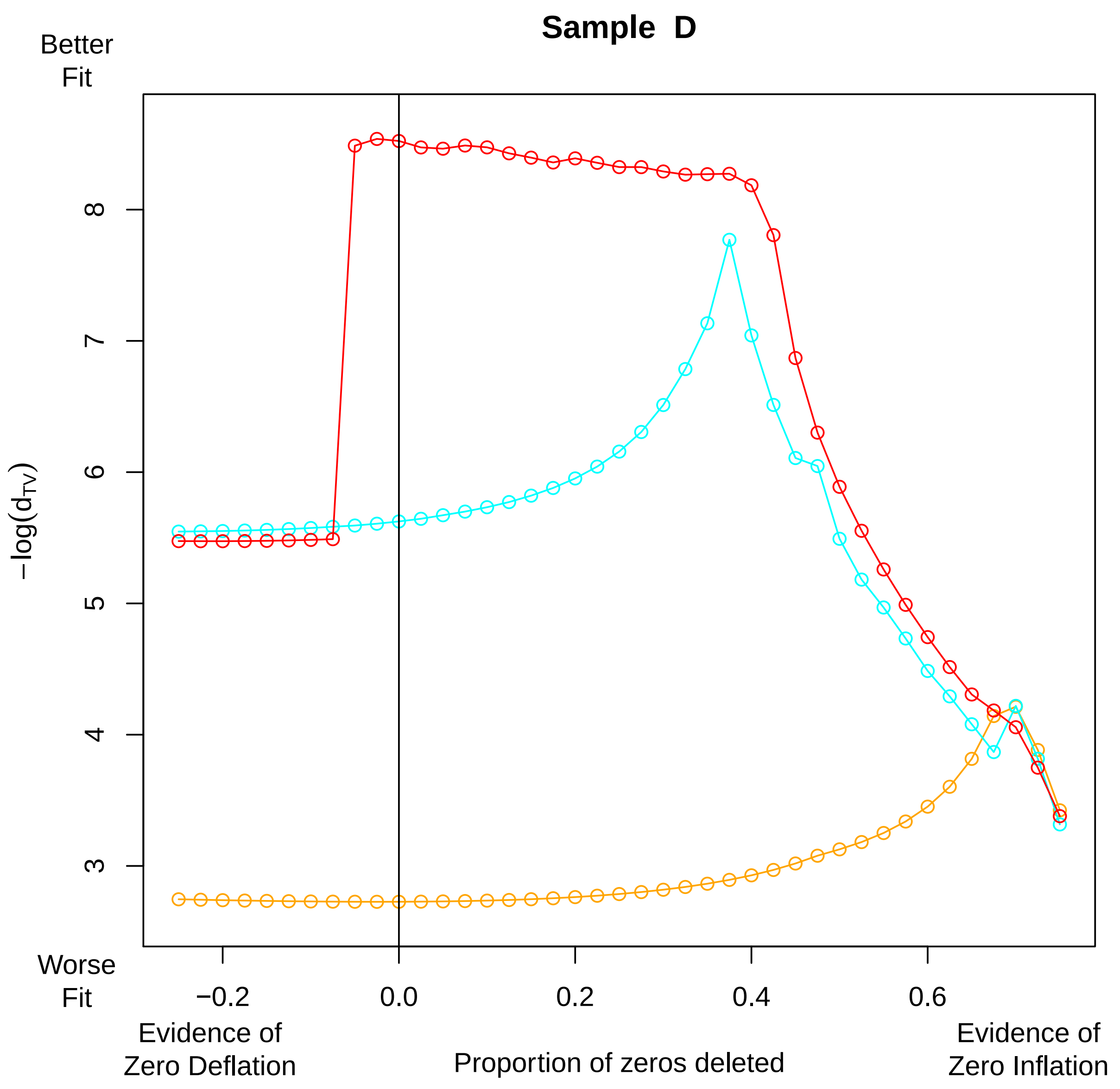

Sample D
Better
Fit
Worse
Fit
−log(d_TV)
3
4
5
6
7
8
−0.2
0.0
0.2
0.4
0.6
Evidence of
Zero Deflation
Proportion of zeros deleted
Evidence of
Zero Inflation

Example plots - Sample A, ChIP track

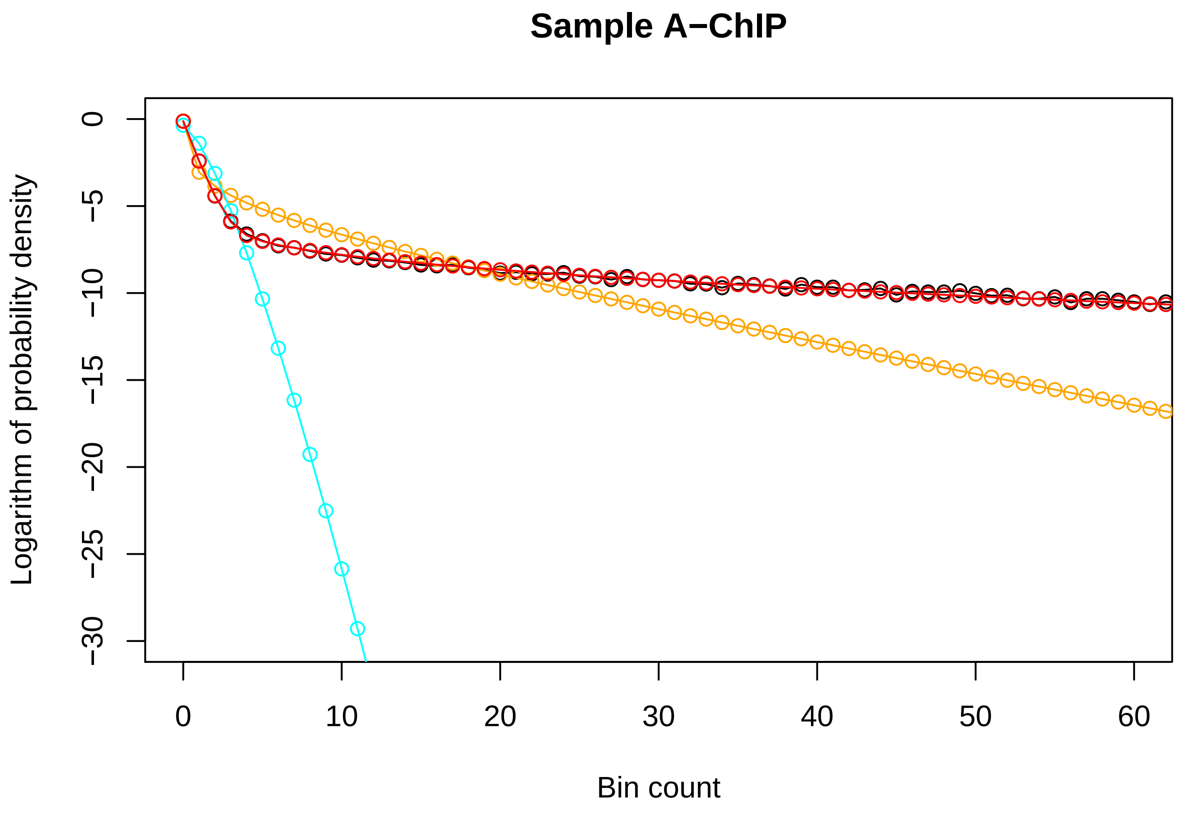

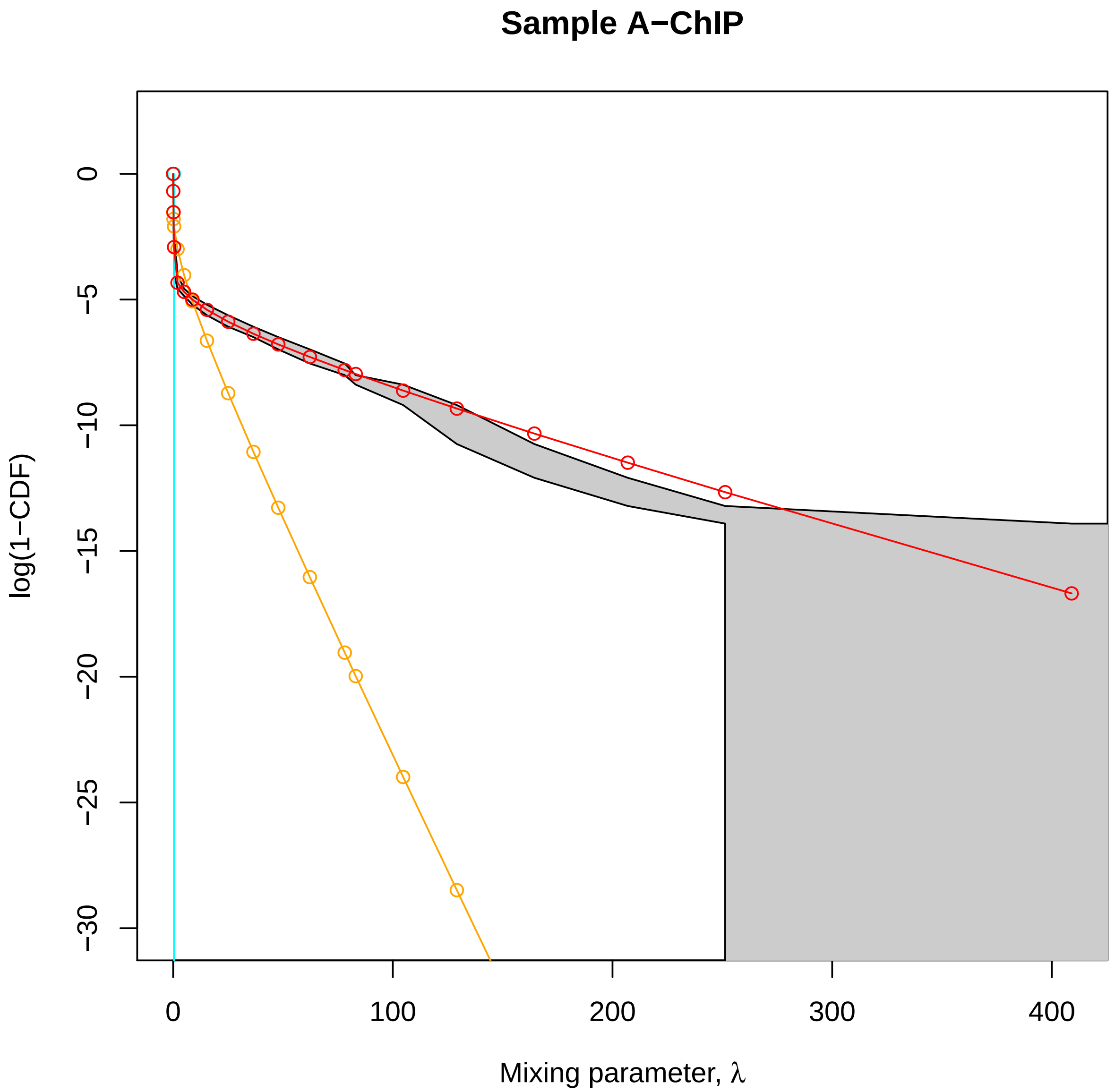
Sample A−ChIP
log(1−CDF)
0
−5
−10
−15
−20
−25
−30
0
100
200
300
400
Mixing parameter, λ

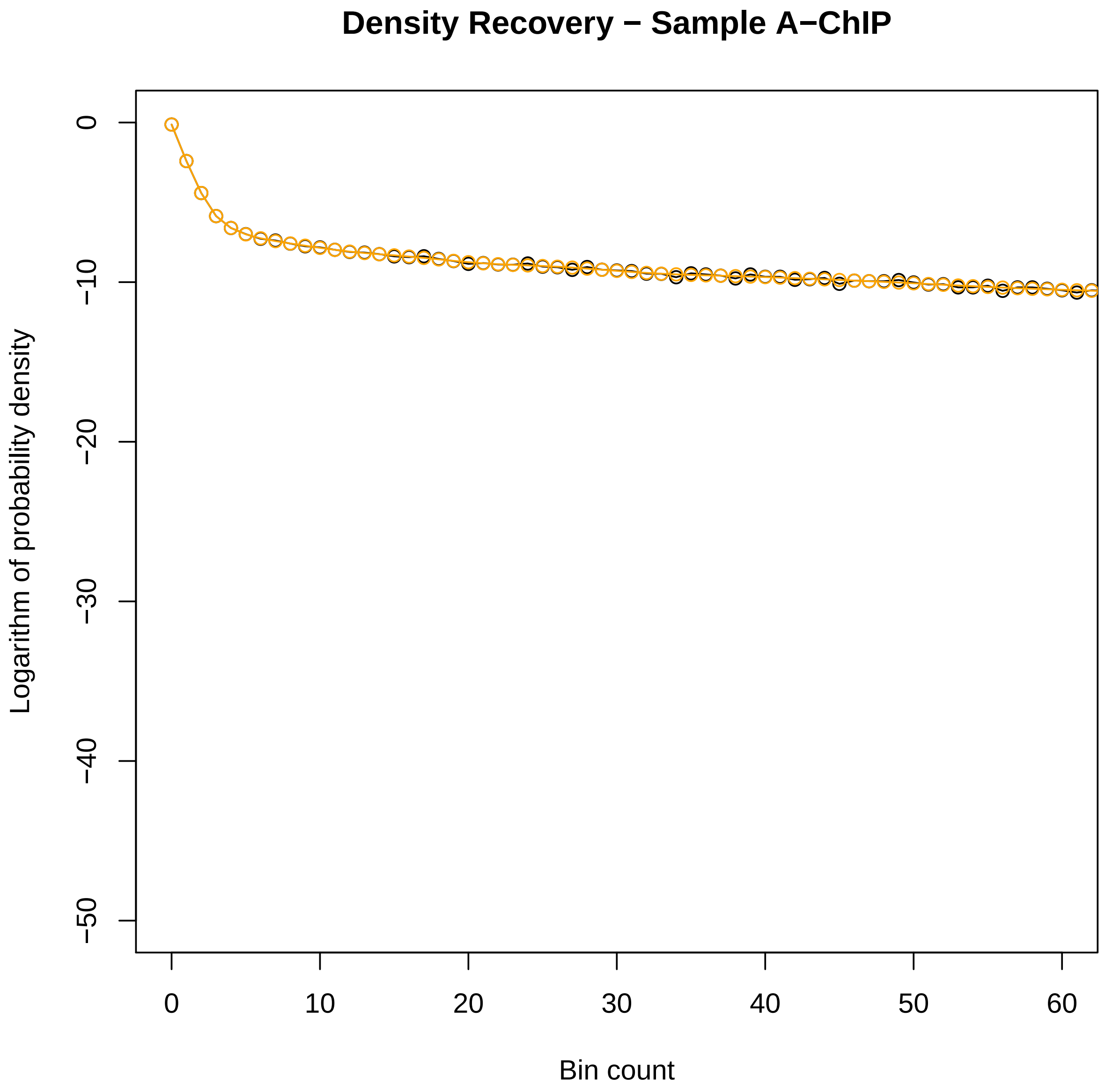
Density Recovery − Sample A−ChIP
Logarithm of probability density
0
−10
−20
−30
−40
−50
0
10
20
30
40
50
60
Bin count

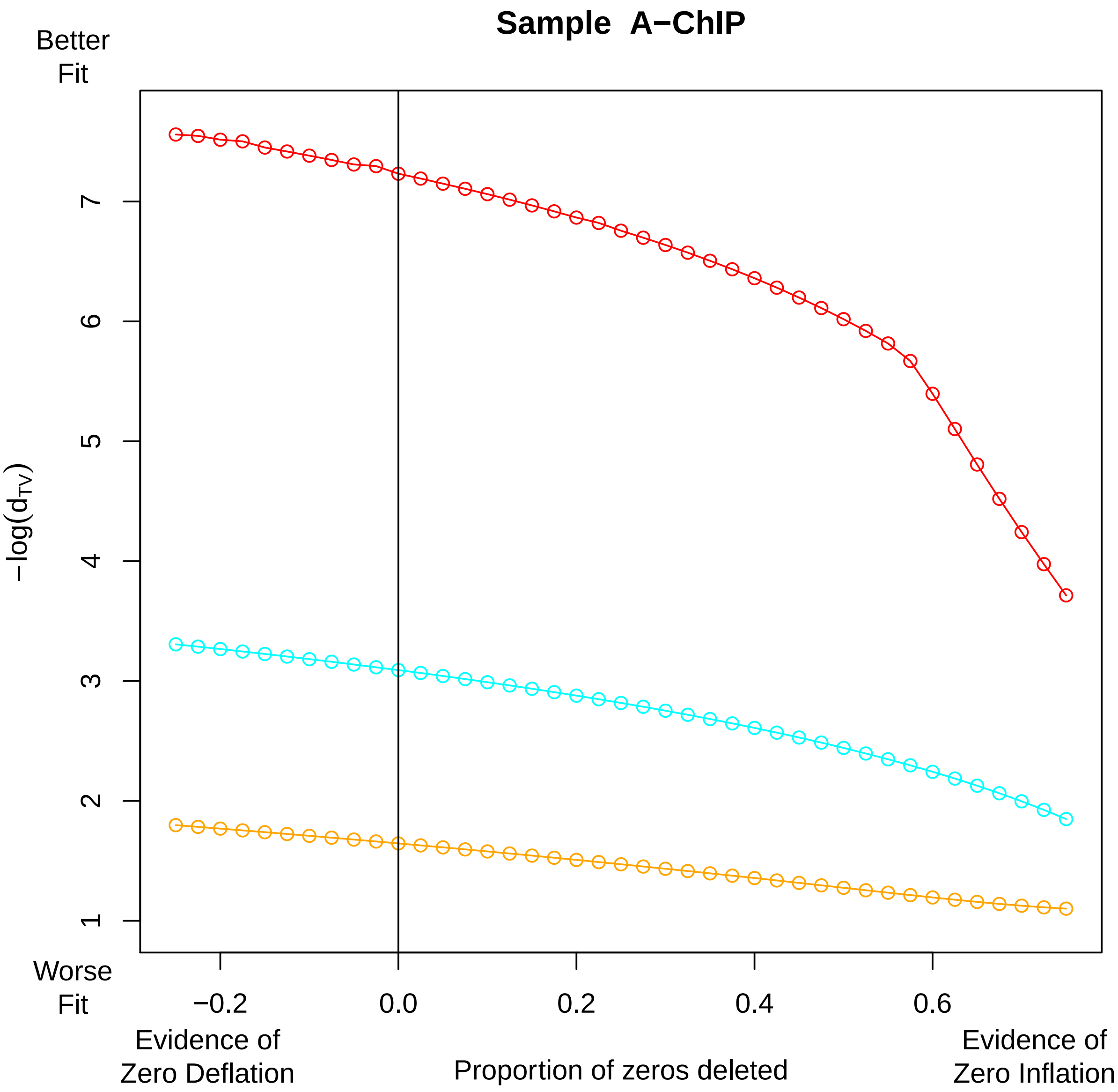
Sample A−ChIP
Better Fit
Worse Fit
−log(d_{TV})
1
2
3
4
5
6
7
−0.2
0.0
0.2
0.4
0.6
Evidence of Zero Deflation
Proportion of zeros deleted
Evidence of Zero Inflation

Example plots - Sample B, ChIP track

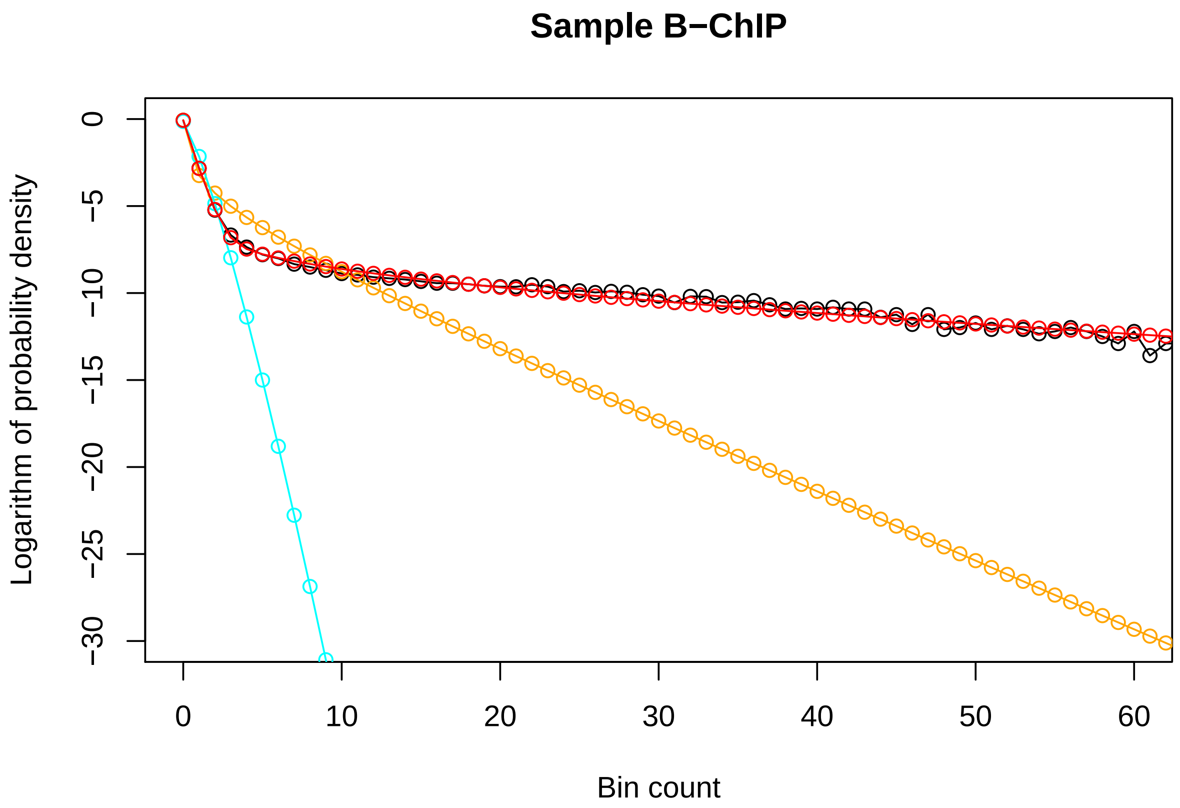

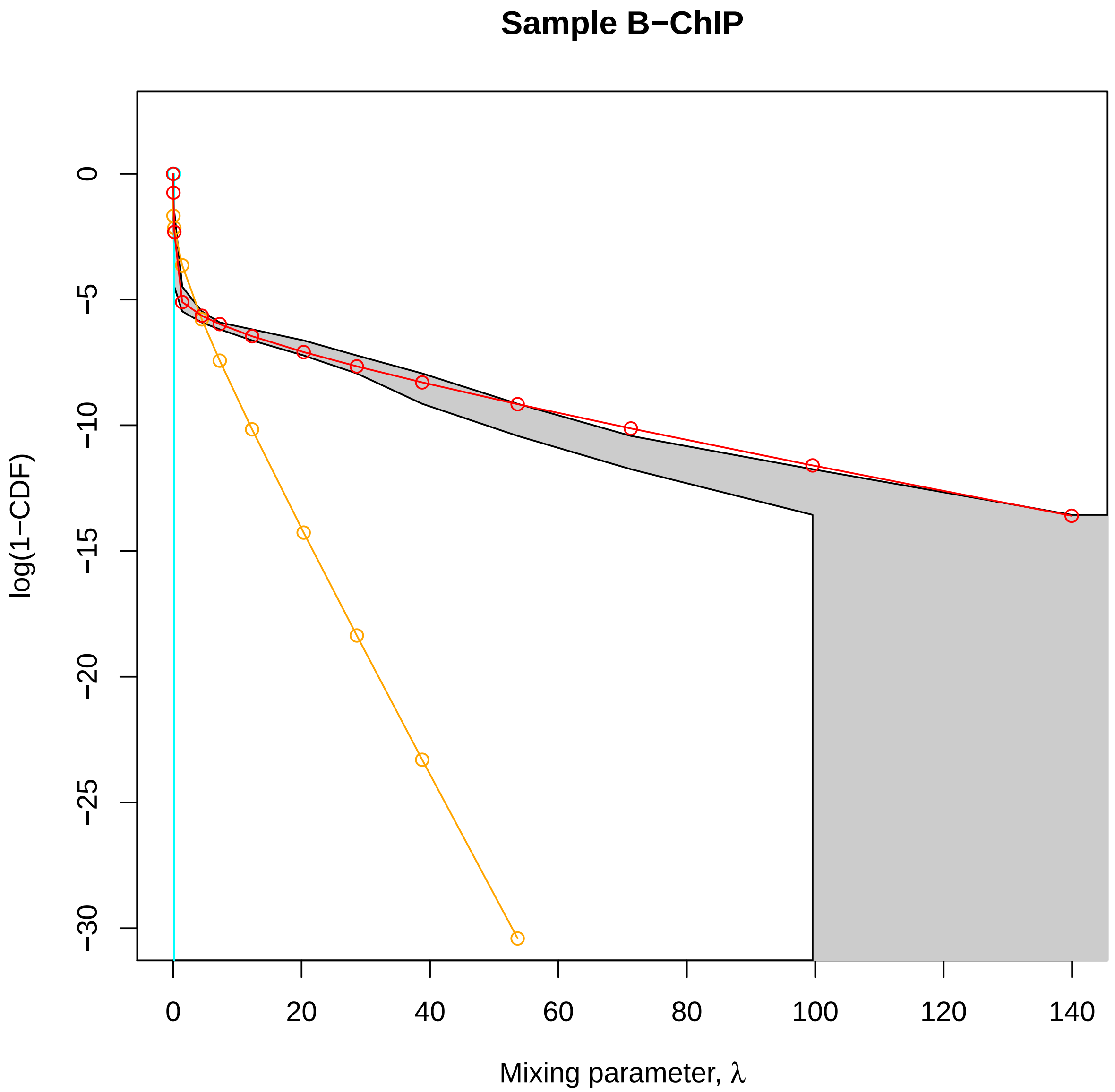

Sample B−ChIP
log(1−CDF)
0
−5
−10
−15
−20
−25
−30
0
20
40
60
80
100
120
140
Mixing parameter, λ

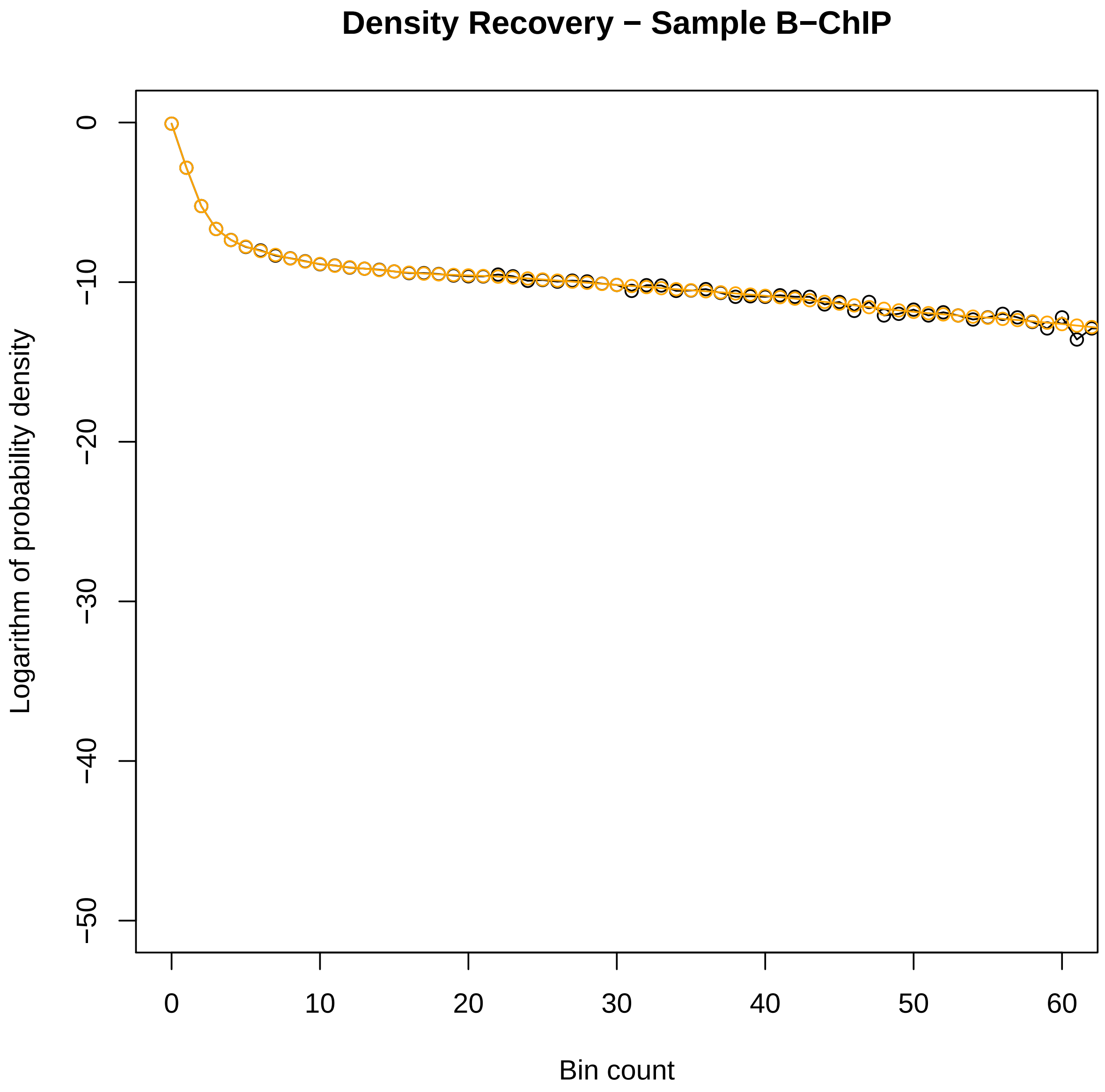

Density Recovery − Sample B−ChIP
Logarithm of probability density
0
−10
−20
−30
−40
−50
0
10
20
30
40
50
60
Bin count

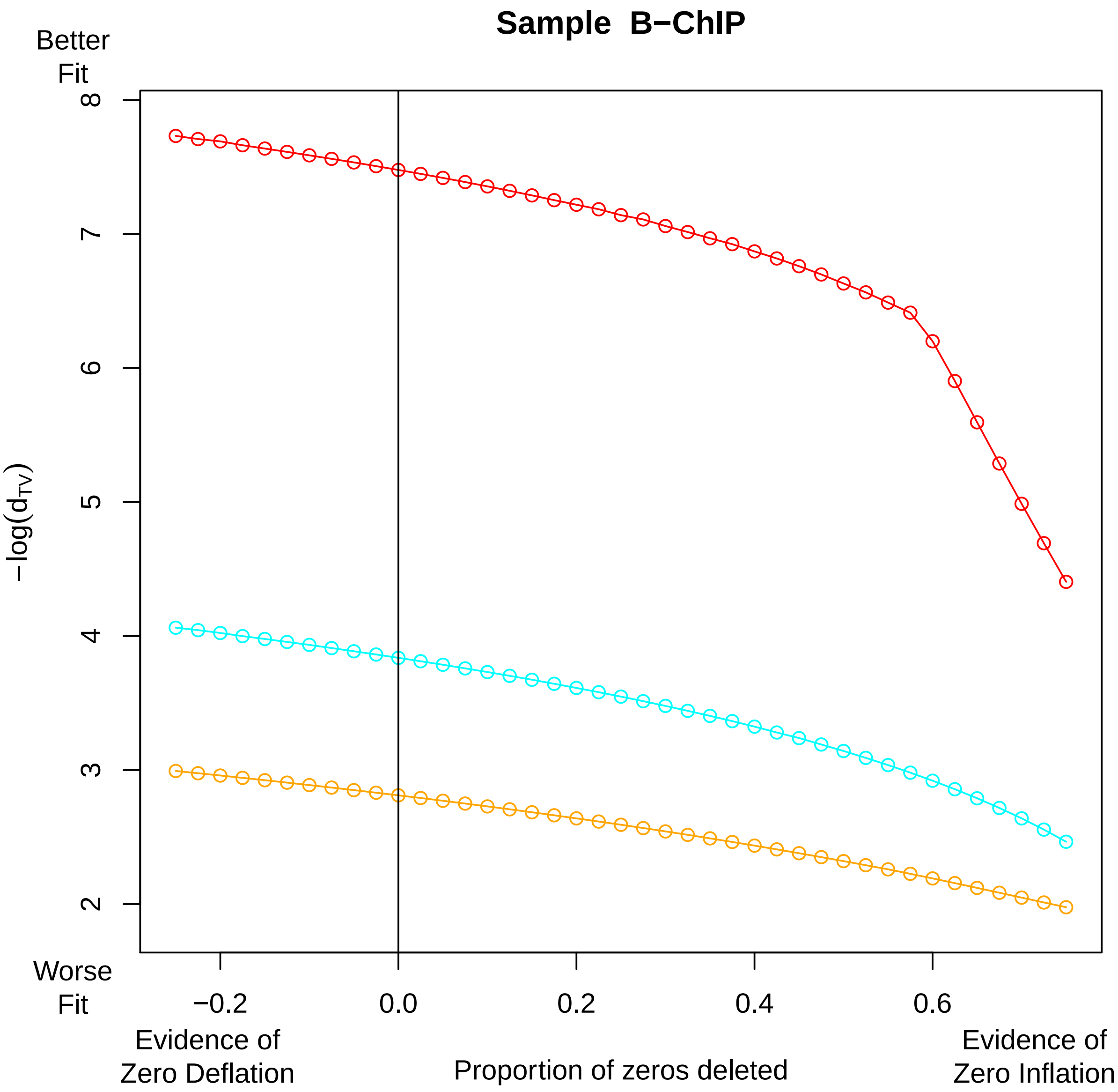
Sample B−ChIP
Better
Fit
Worse
Fit
−log(d_TV)
8
7
6
5
4
3
2
−0.2
0.0
0.2
0.4
0.6
Evidence of
Zero Deflation
Proportion of zeros deleted
Evidence of
Zero Inflation

Example plots - Sample C, ChIP track

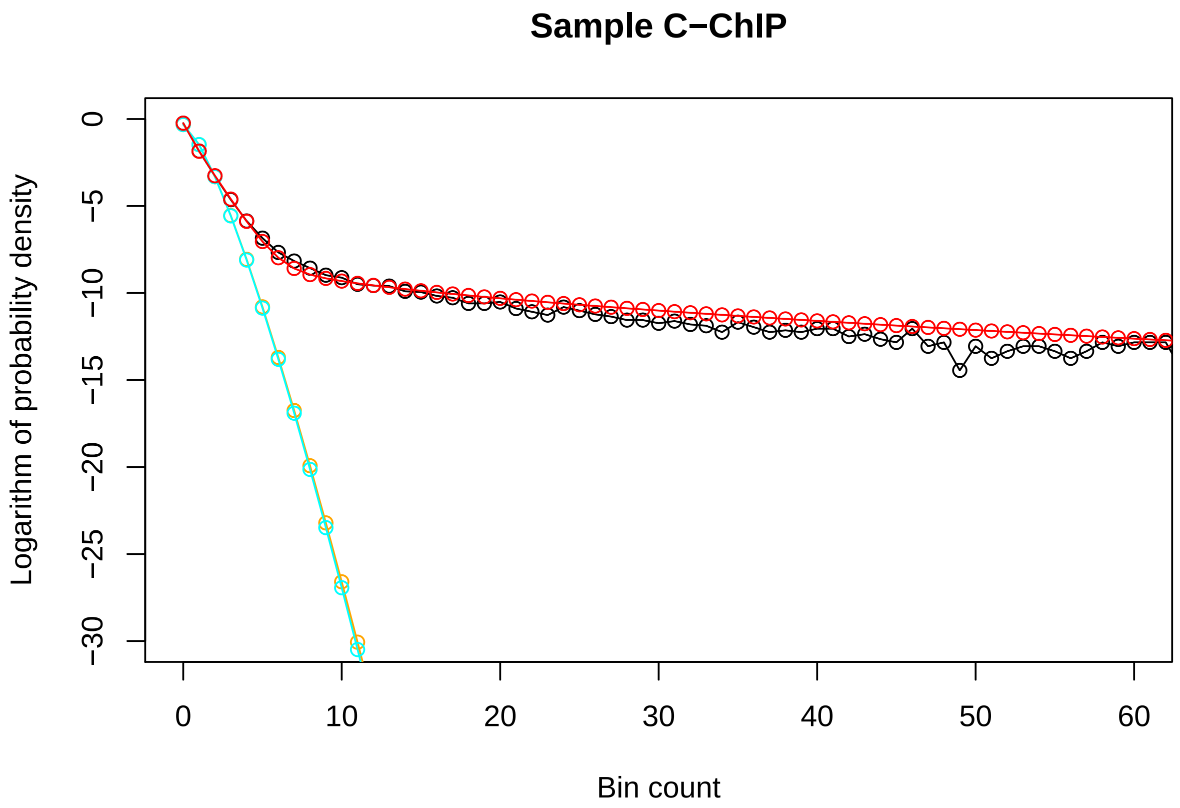

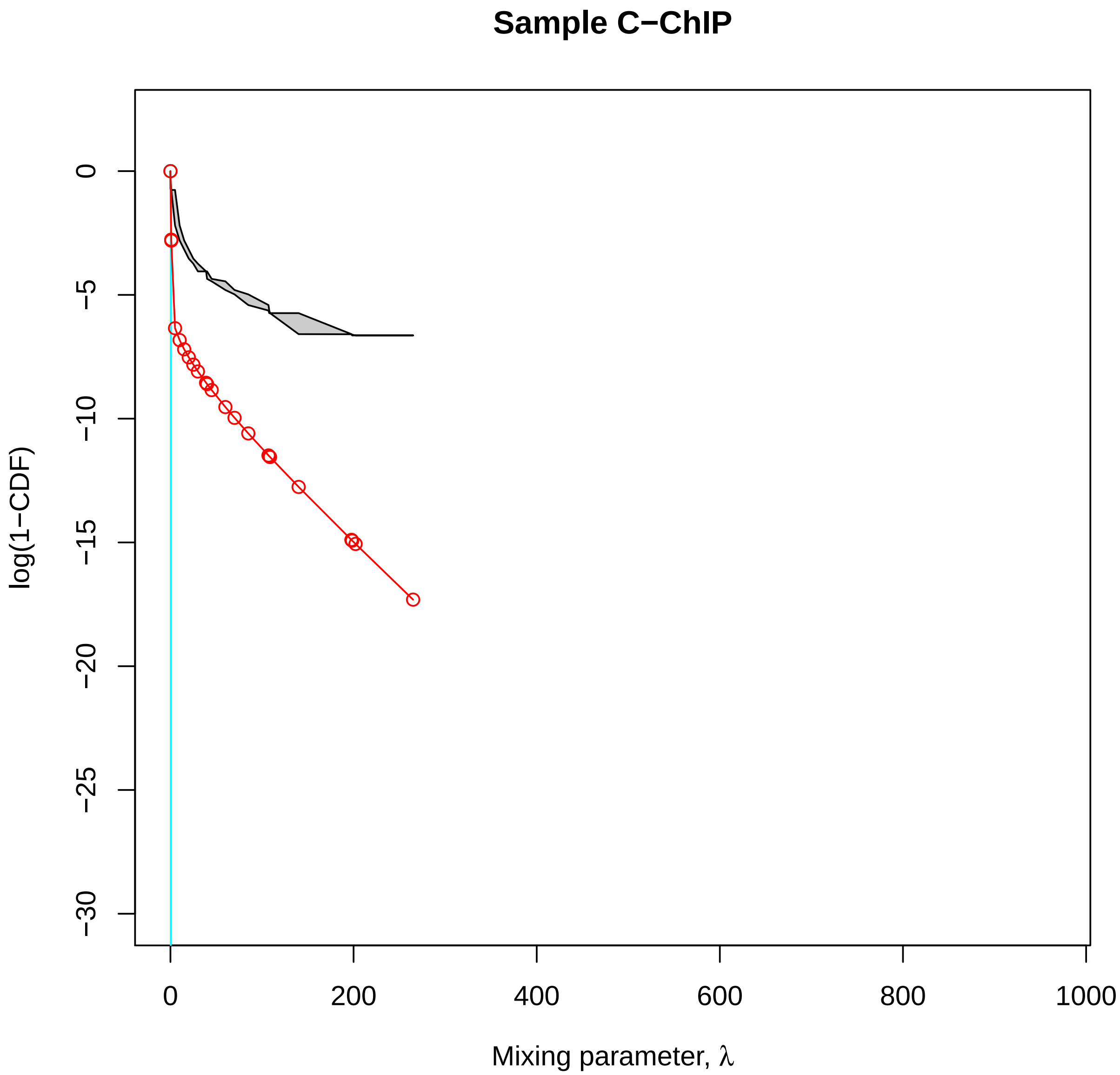
Sample C−ChIP
log(1−CDF)
0
−5
−10
−15
−20
−25
−30
0
200
400
600
800
1000
Mixing parameter, λ

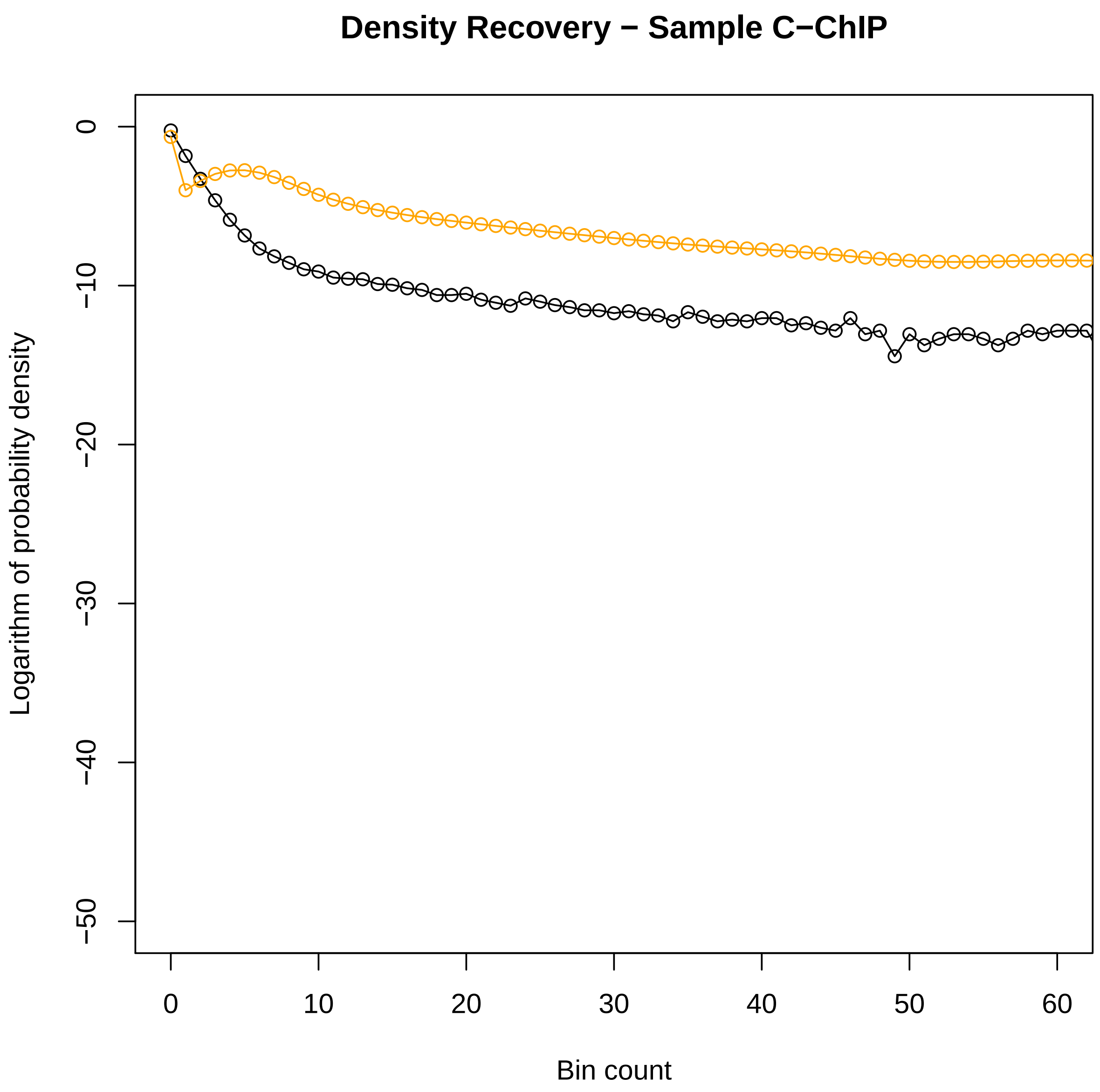

Density Recovery − Sample C−ChIP
Logarithm of probability density
Bin count
0
−10
−20
−30
−40
−50
0
10
20
30
40
50
60

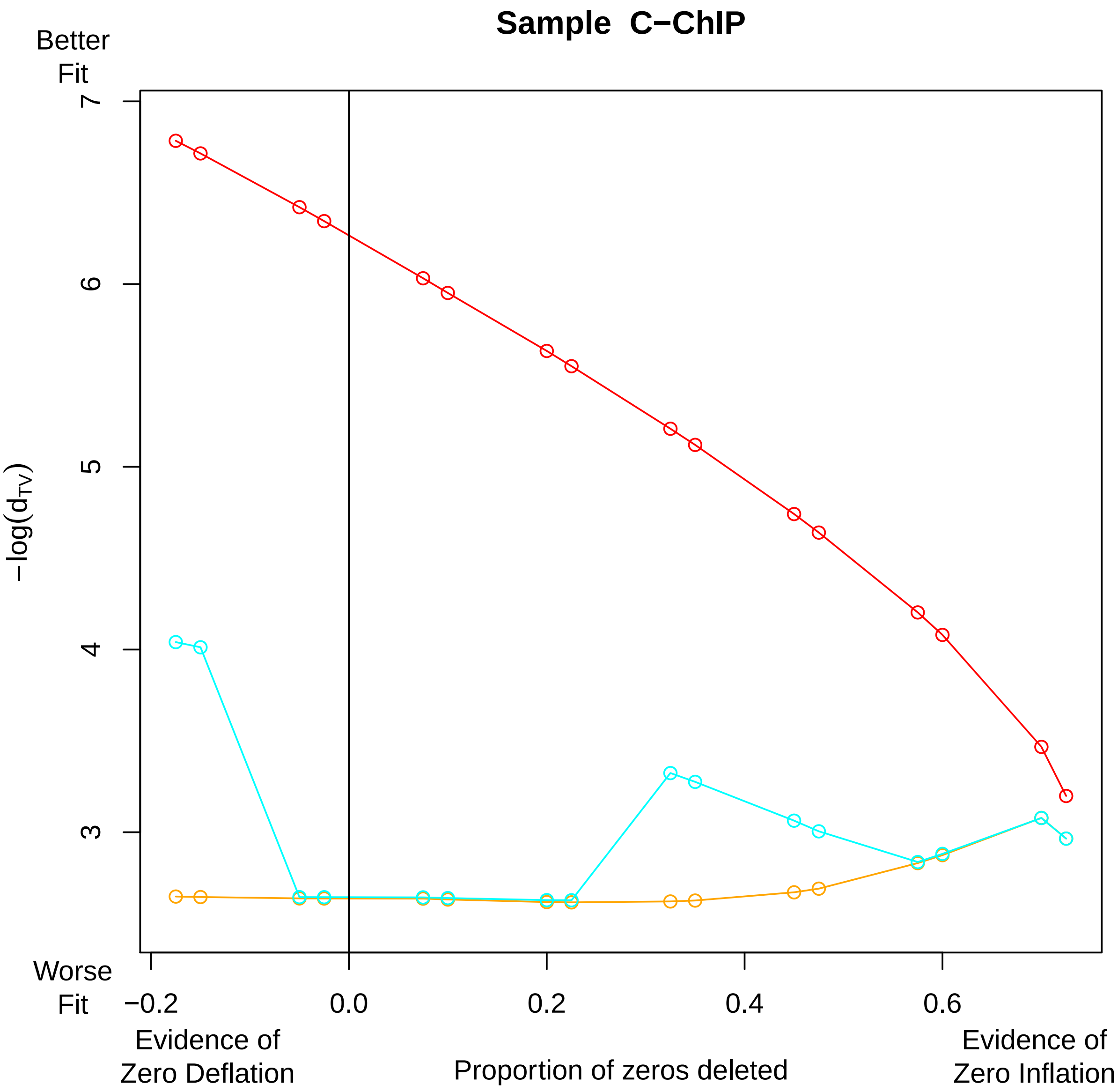

Sample C−ChIP
Better Fit
Worse Fit
−log(d_{TV})
7
6
5
4
3
−0.2
0.0
0.2
0.4
0.6
Evidence of Zero Deflation
Proportion of zeros deleted
Evidence of Zero Inflation

Example plots - Sample D, ChIP track

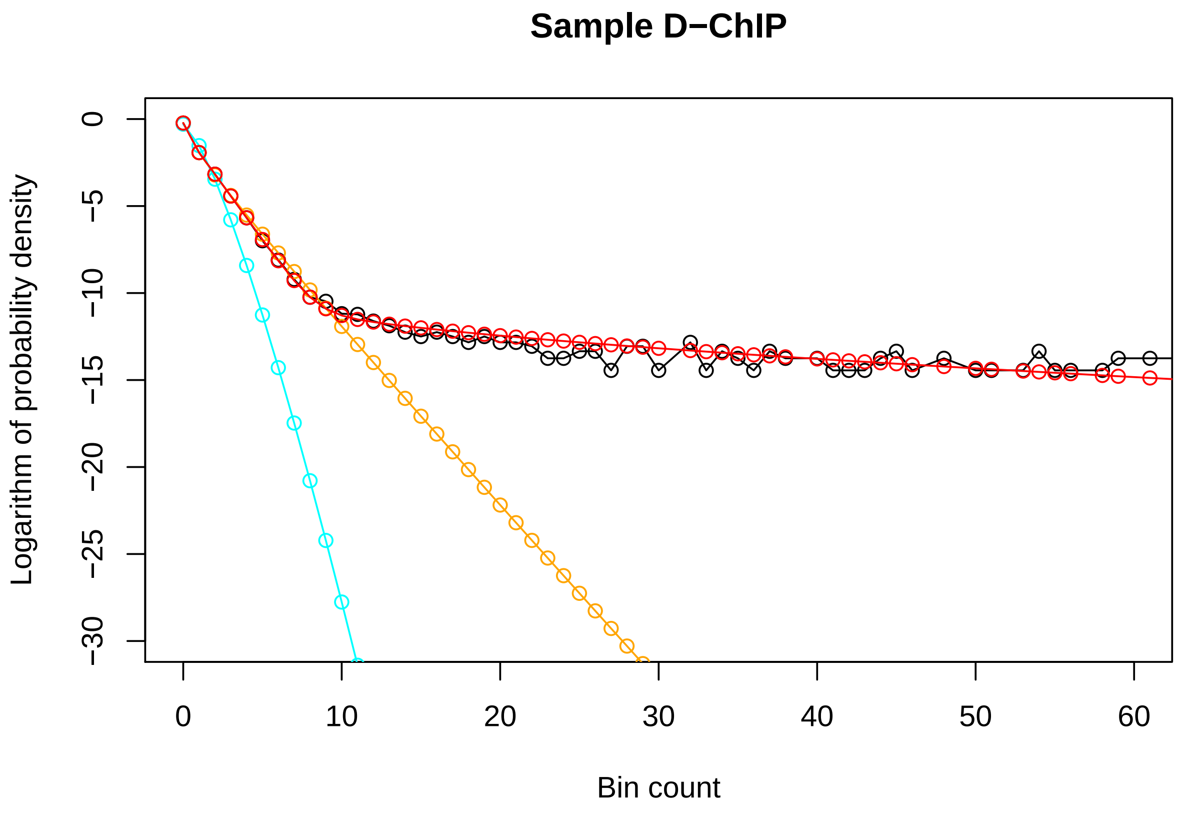

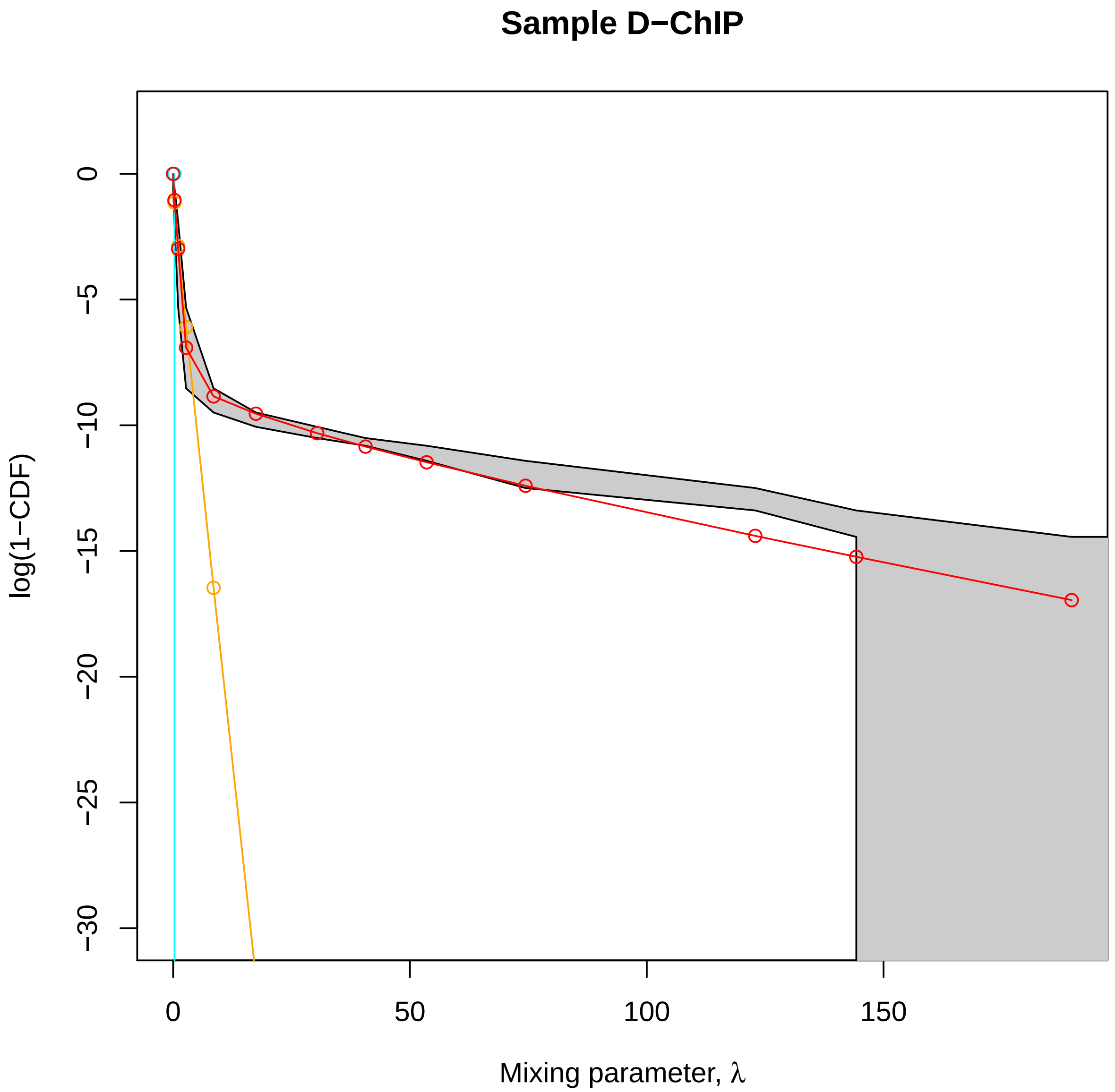
Sample D−ChIP
0
−5
−10
−15
−20
−25
−30
log(1−CDF)
0
50
100
150
Mixing parameter, λ

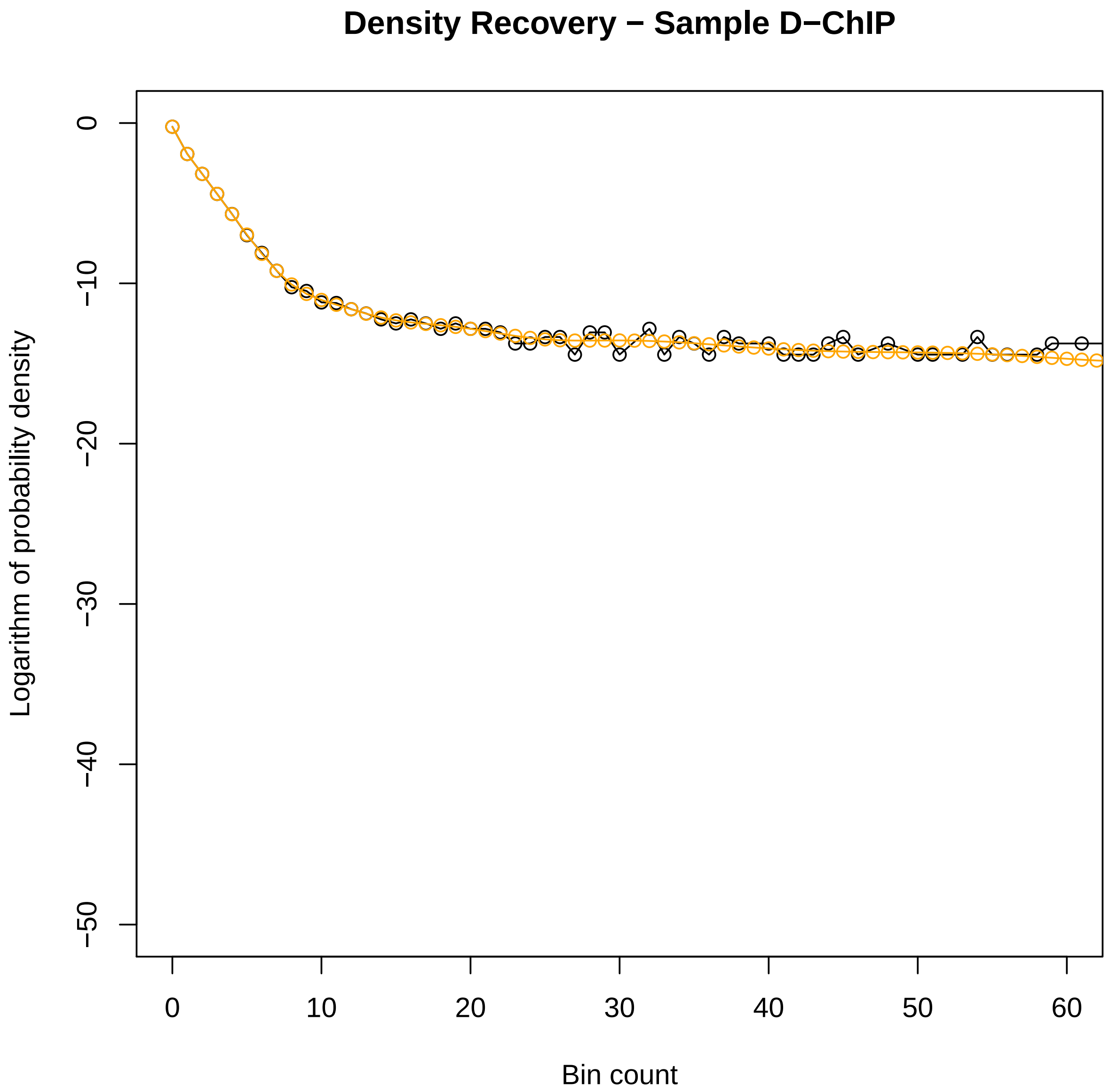
Density Recovery − Sample D−ChIP
Logarithm of probability density
Bin count
0
−10
−20
−30
−40
−50
0
10
20
30
40
50
60

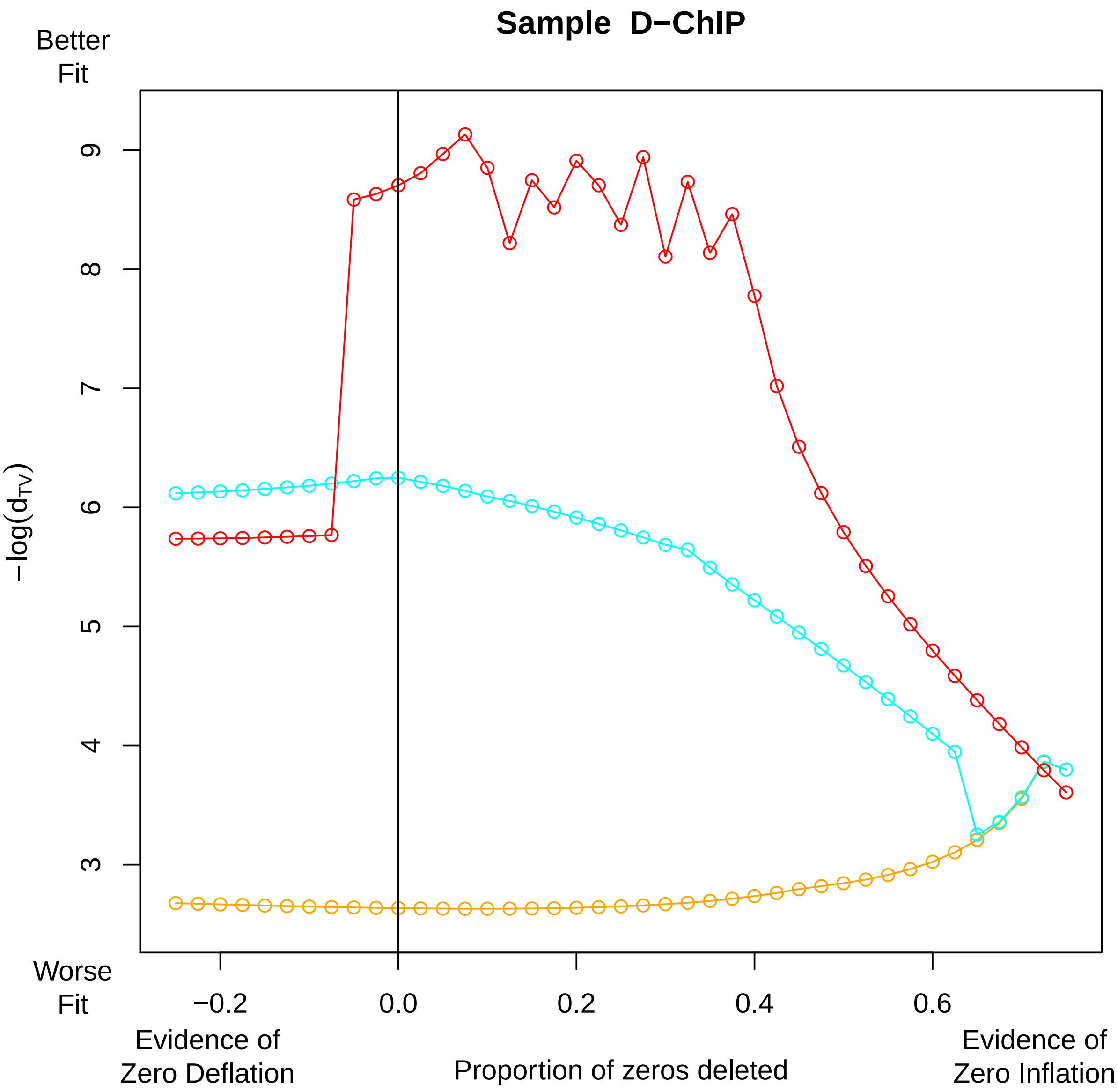

Sample D−ChIP
Better Fit
Worse Fit
−log(d_{TV})
9
8
7
6
5
4
3
−0.2
0.0
0.2
0.4
0.6
Evidence of Zero Deflation
Proportion of zeros deleted
Evidence of Zero Inflation

Example plots - Sample A, duplicates removed

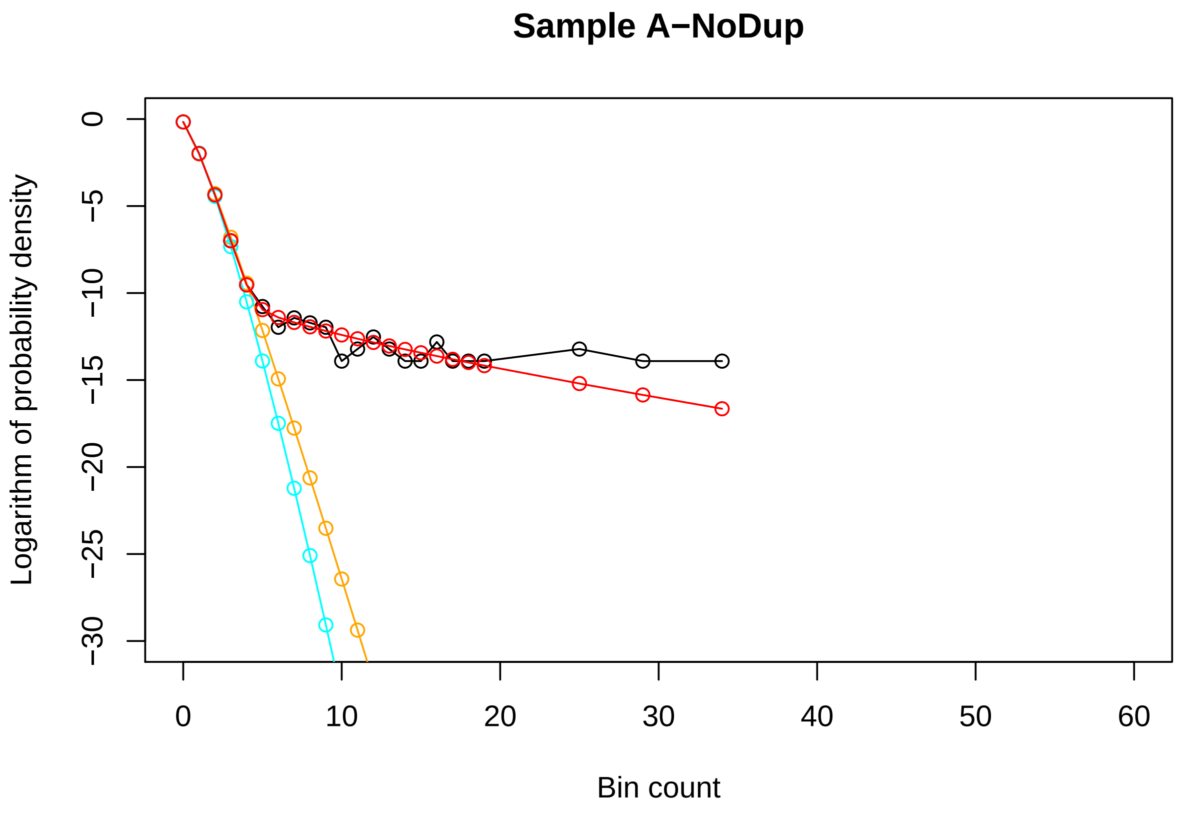

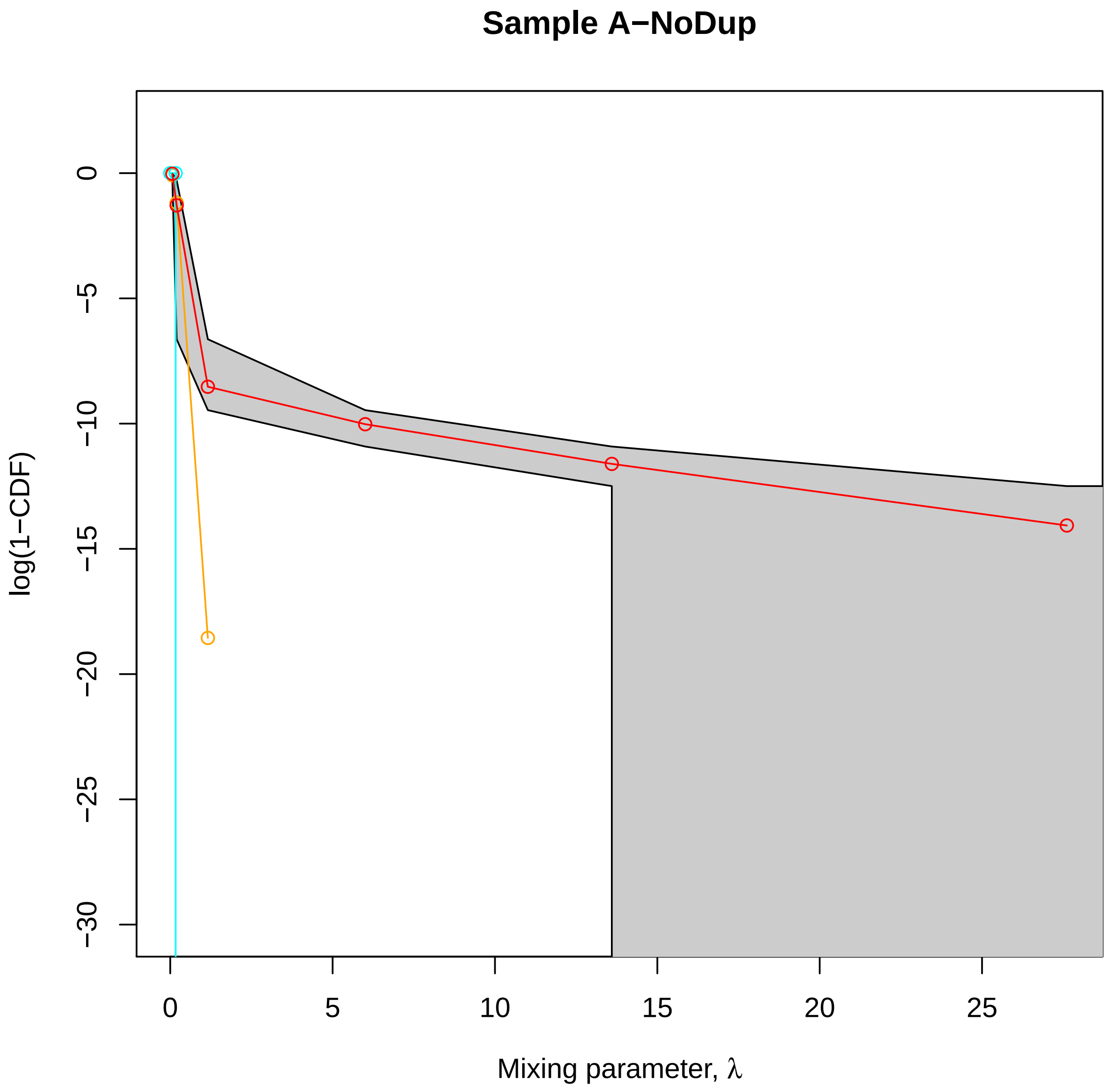
Sample A−NoDup
0
−5
−10
−15
−20
−25
−30
log(1−CDF)
0
5
10
15
20
25
Mixing parameter, λ

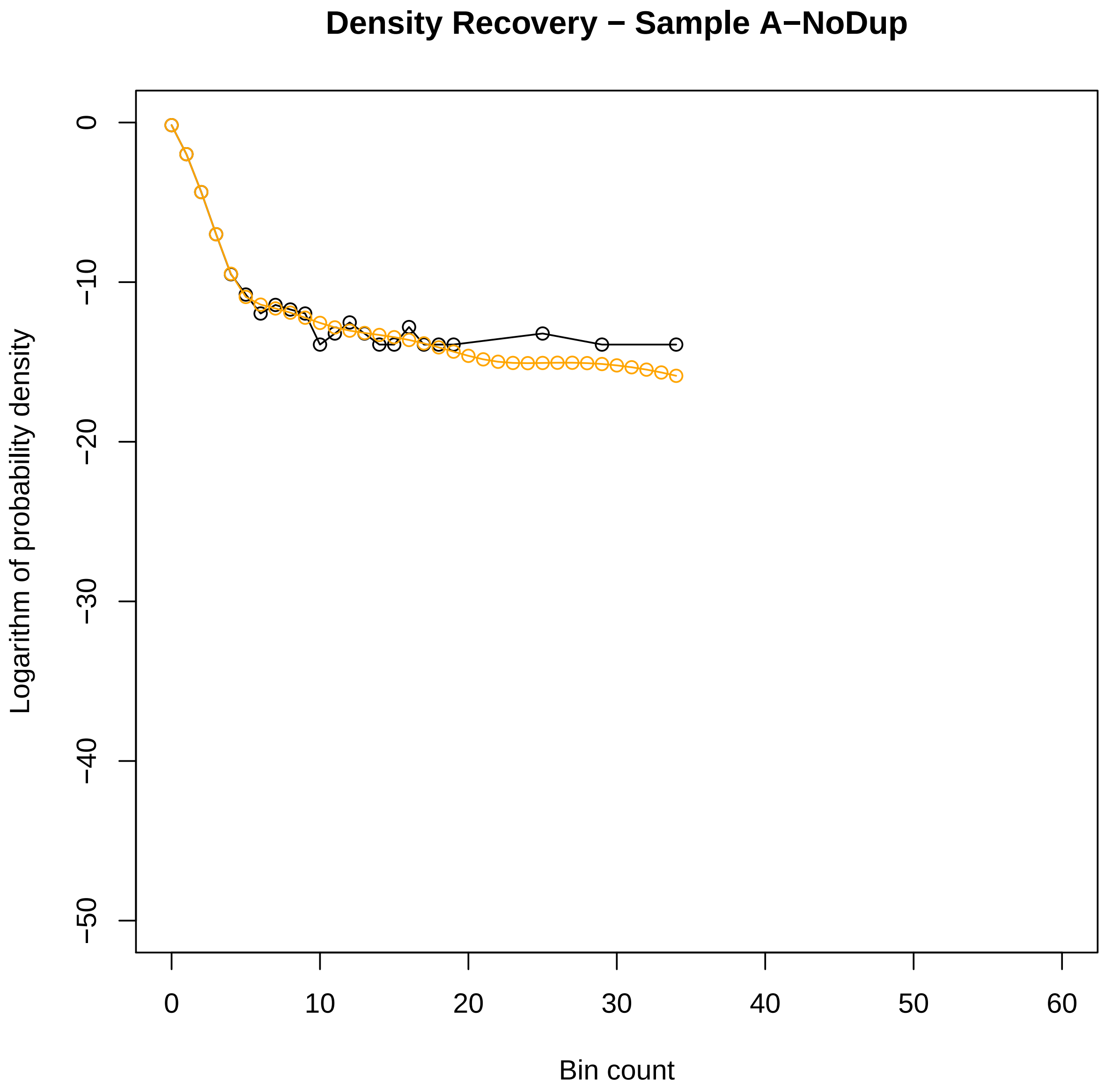

Density Recovery − Sample A−NoDup
Logarithm of probability density
0
−10
−20
−30
−40
−50
0
10
20
30
40
50
60
Bin count

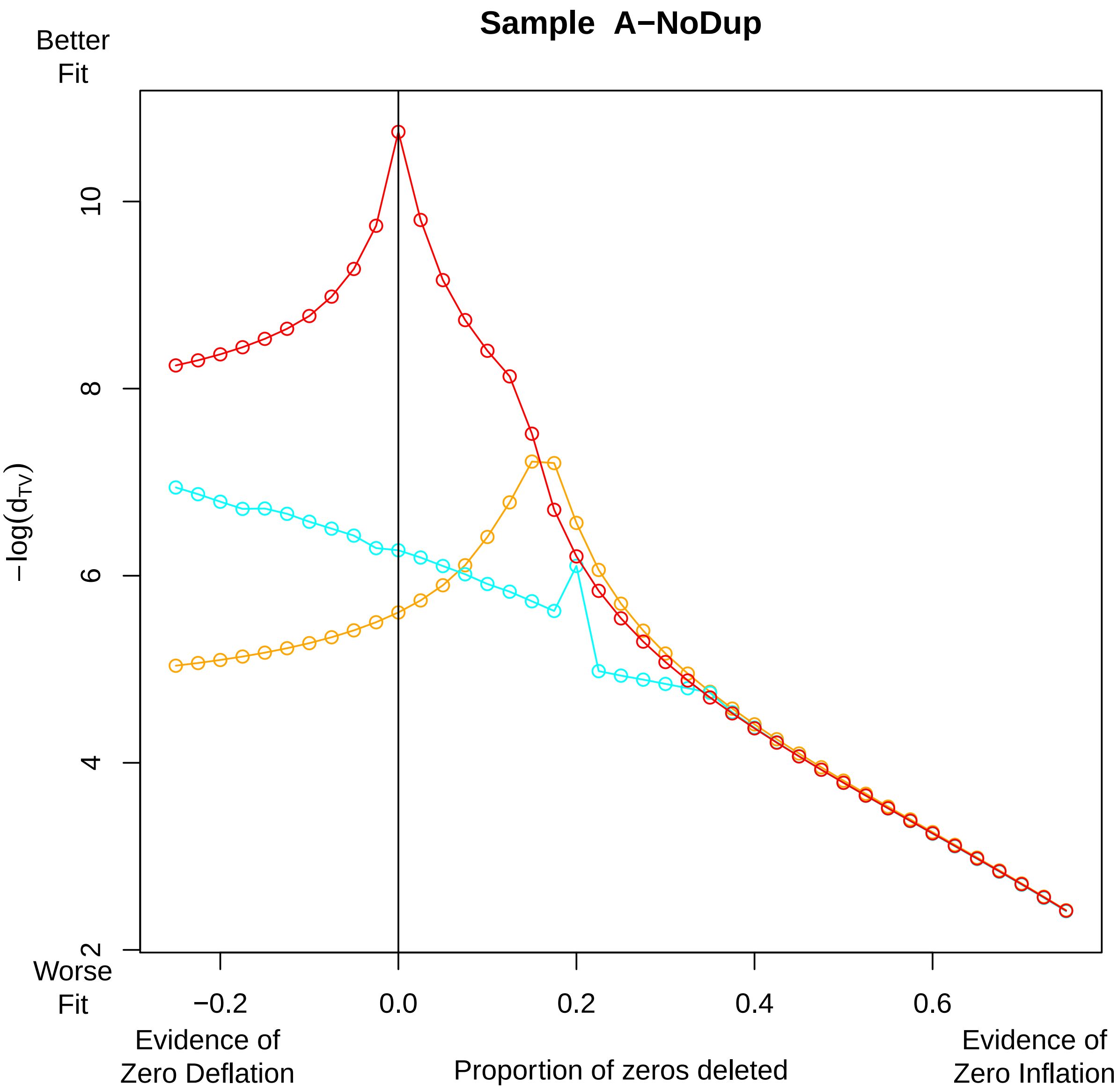

Sample A−NoDup
Better Fit
Worse Fit
−log(d_TV)
2
4
6
8
10
−0.2
0.0
0.2
0.4
0.6
Evidence of Zero Deflation
Proportion of zeros deleted
Evidence of Zero Inflation

Example plots - Sample B, duplicates removed

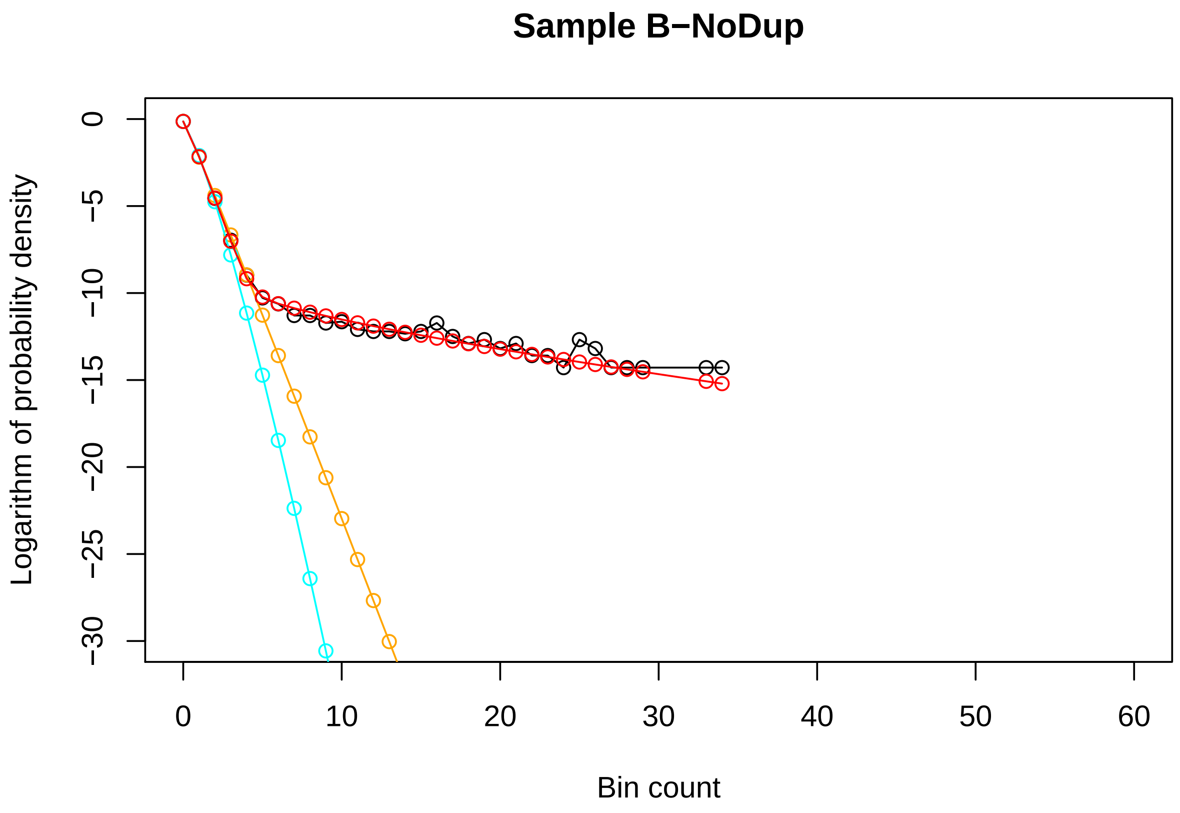

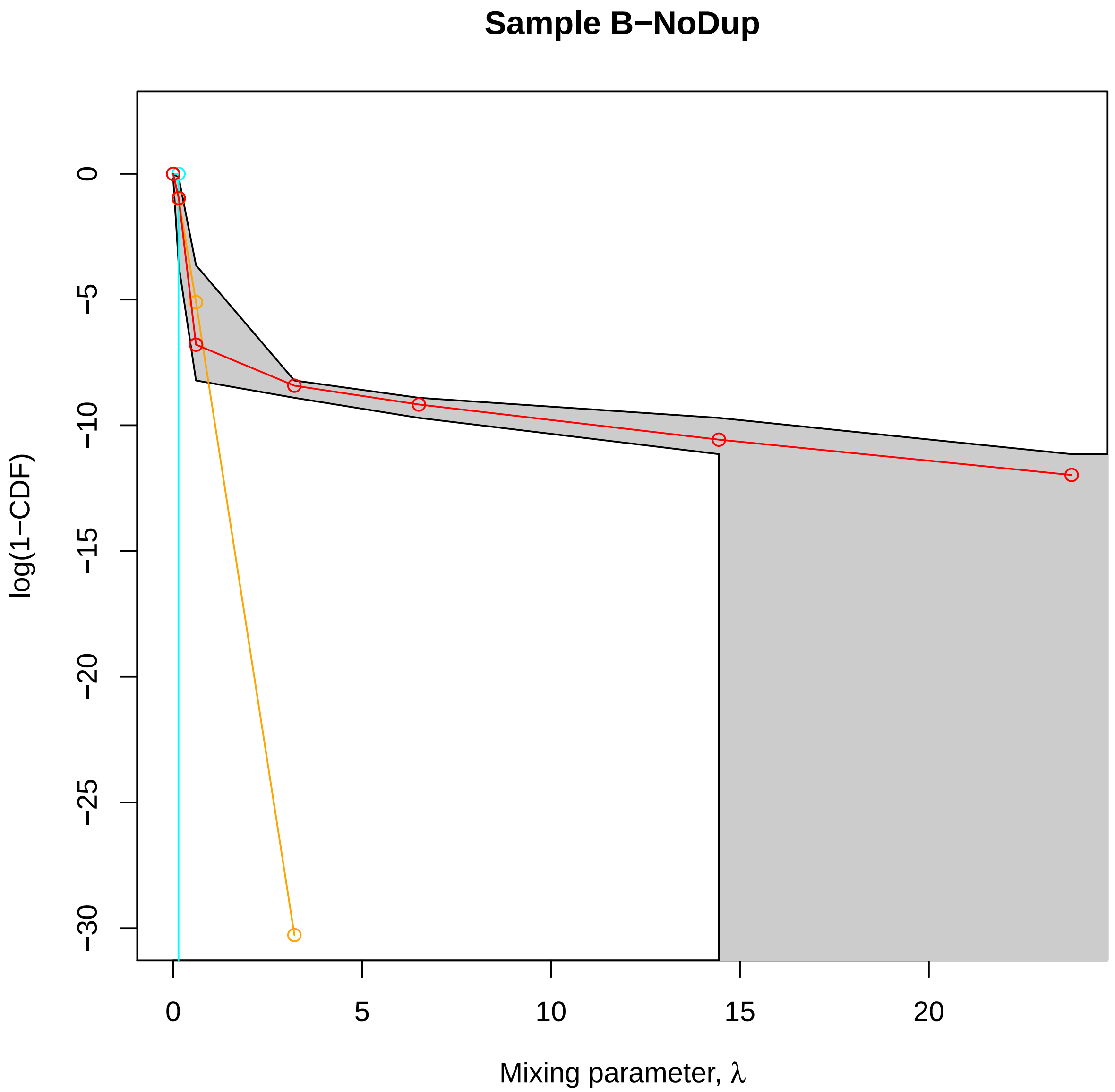
Sample B−NoDup
log(1−CDF)
0
−5
−10
−15
−20
−25
−30
0
5
10
15
20
Mixing parameter, λ

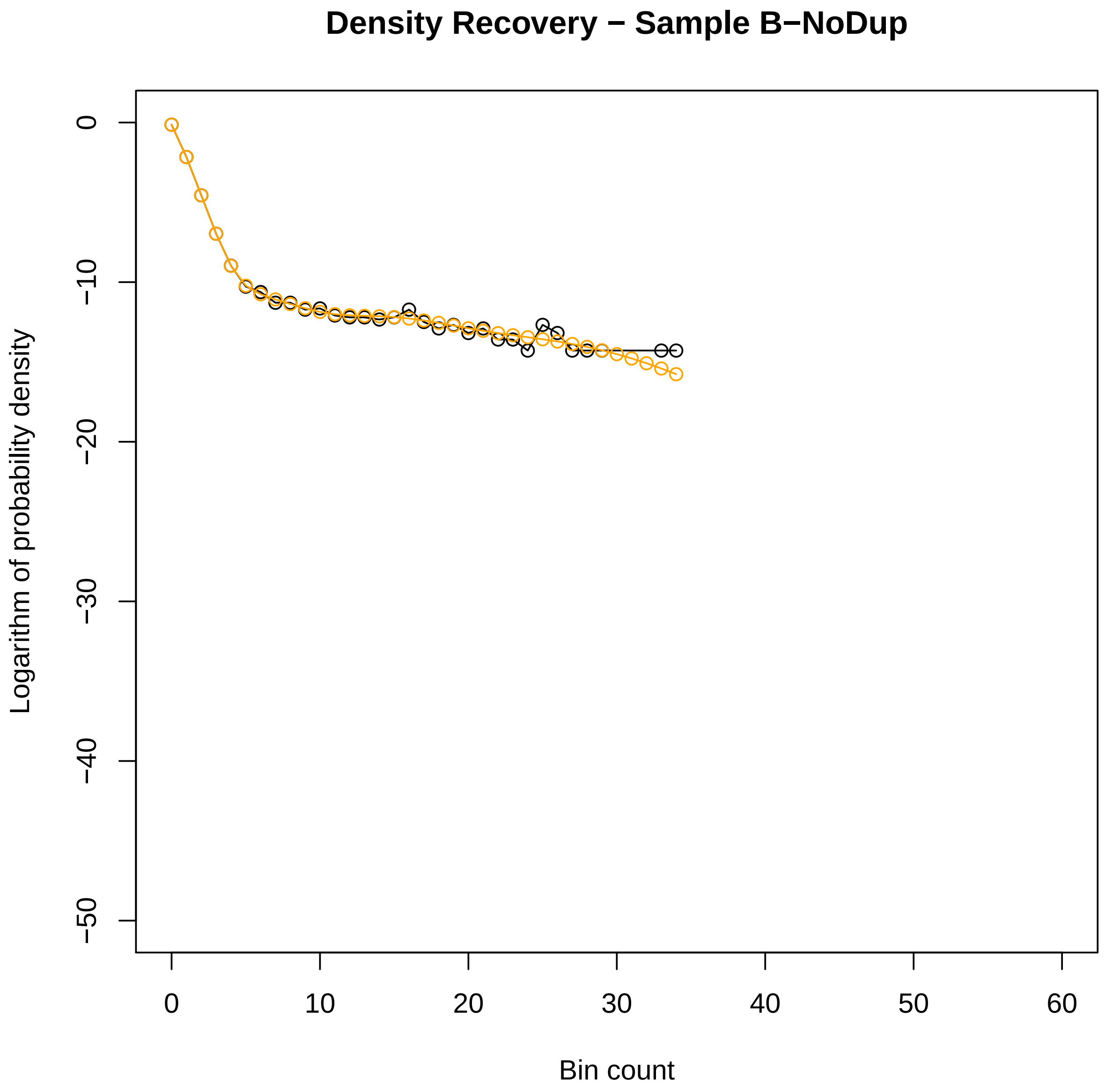

Density Recovery − Sample B−NoDup
Logarithm of probability density
0
−10
−20
−30
−40
−50
0
10
20
30
40
50
60
Bin count

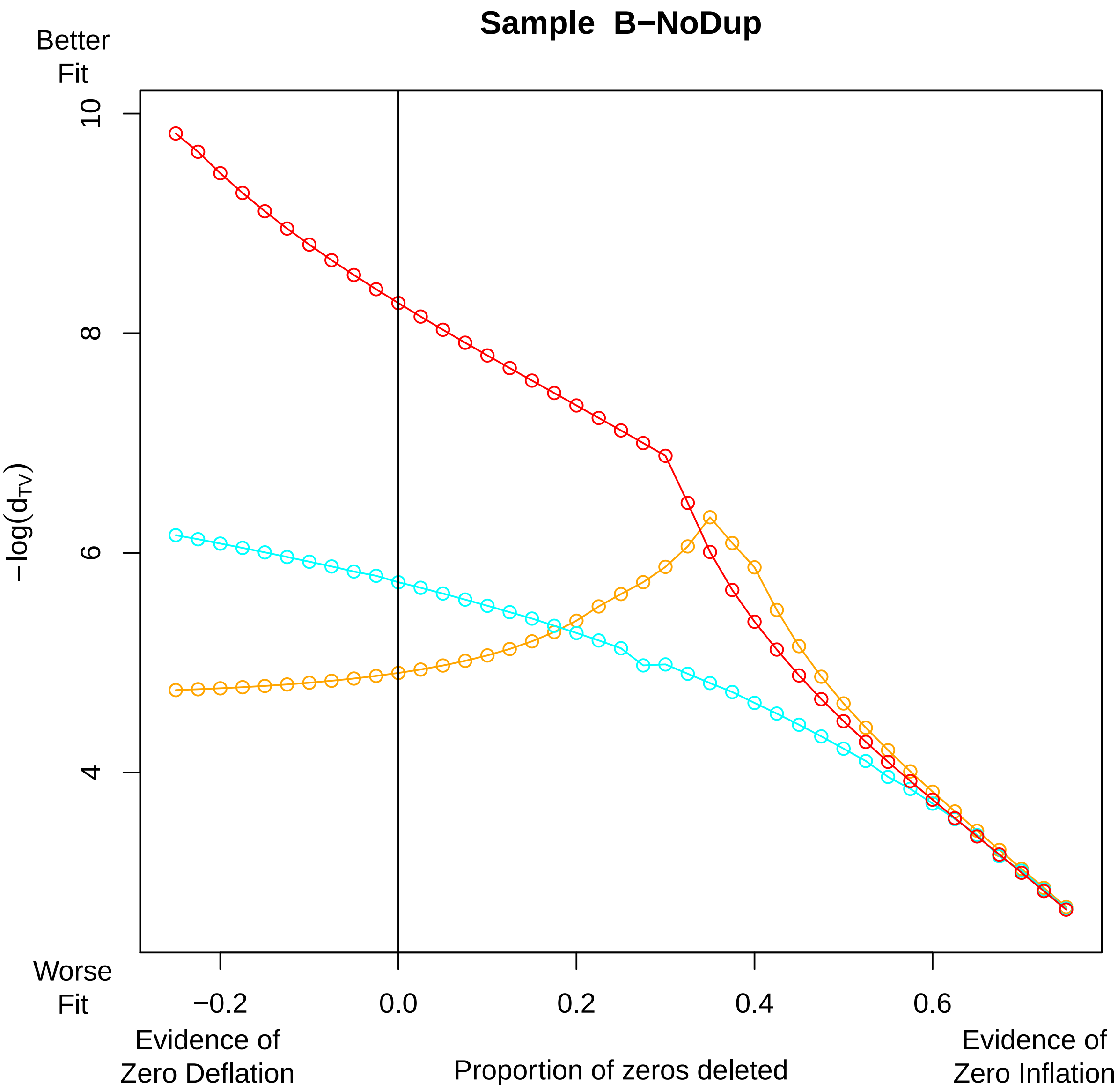
Sample B−NoDup
Better Fit
Worse Fit
−log(d_TV)
10
8
6
4
−0.2
0.0
0.2
0.4
0.6
Evidence of Zero Deflation
Proportion of zeros deleted
Evidence of Zero Inflation

Example plots - Sample C, duplicates removed

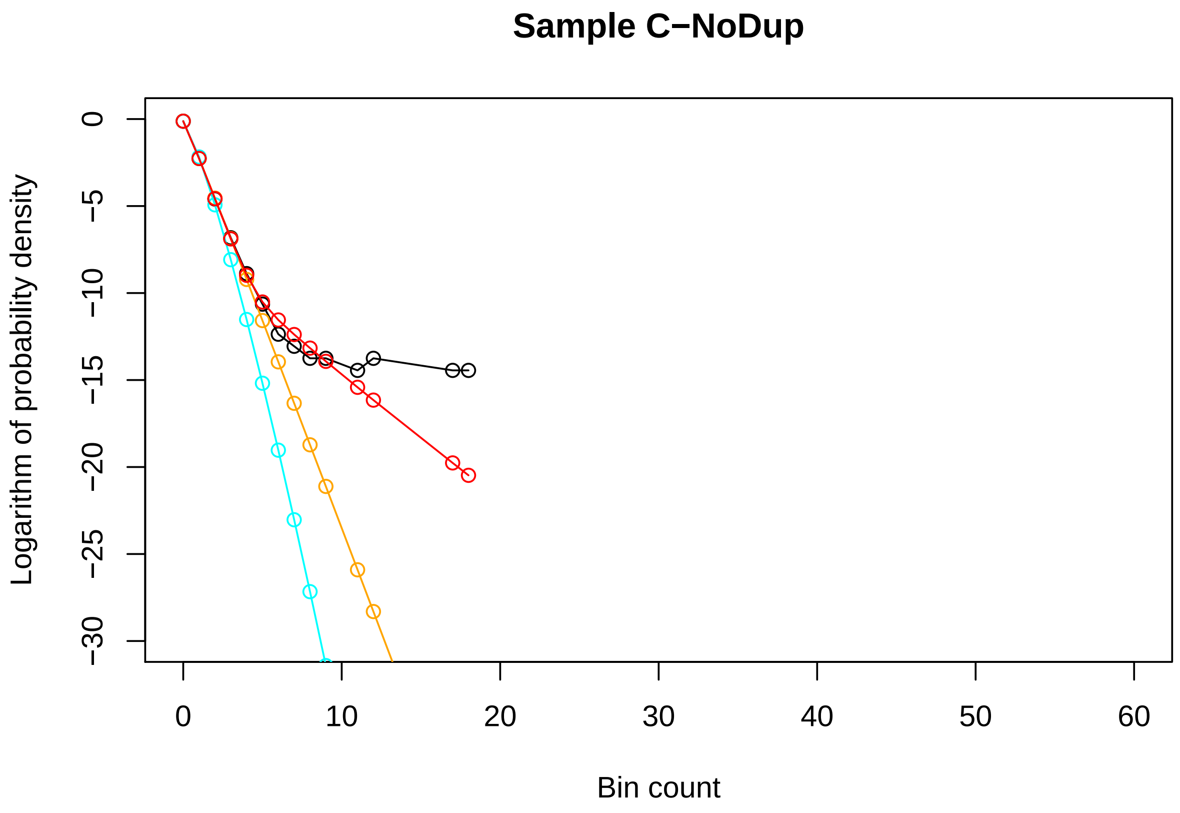

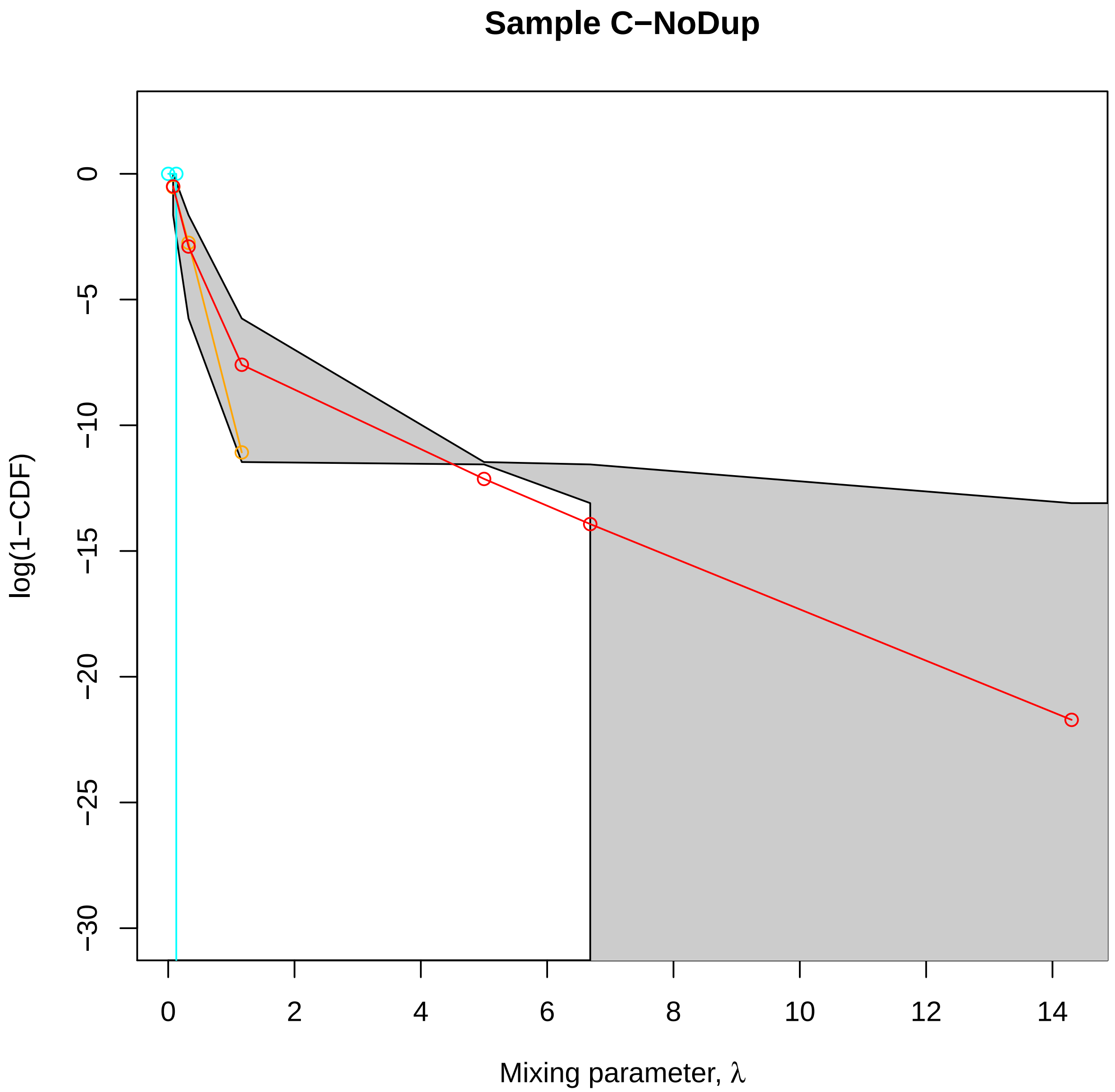

Sample C−NoDup
log(1−CDF)
0
−5
−10
−15
−20
−25
−30
0
2
4
6
8
10
12
14
Mixing parameter, λ

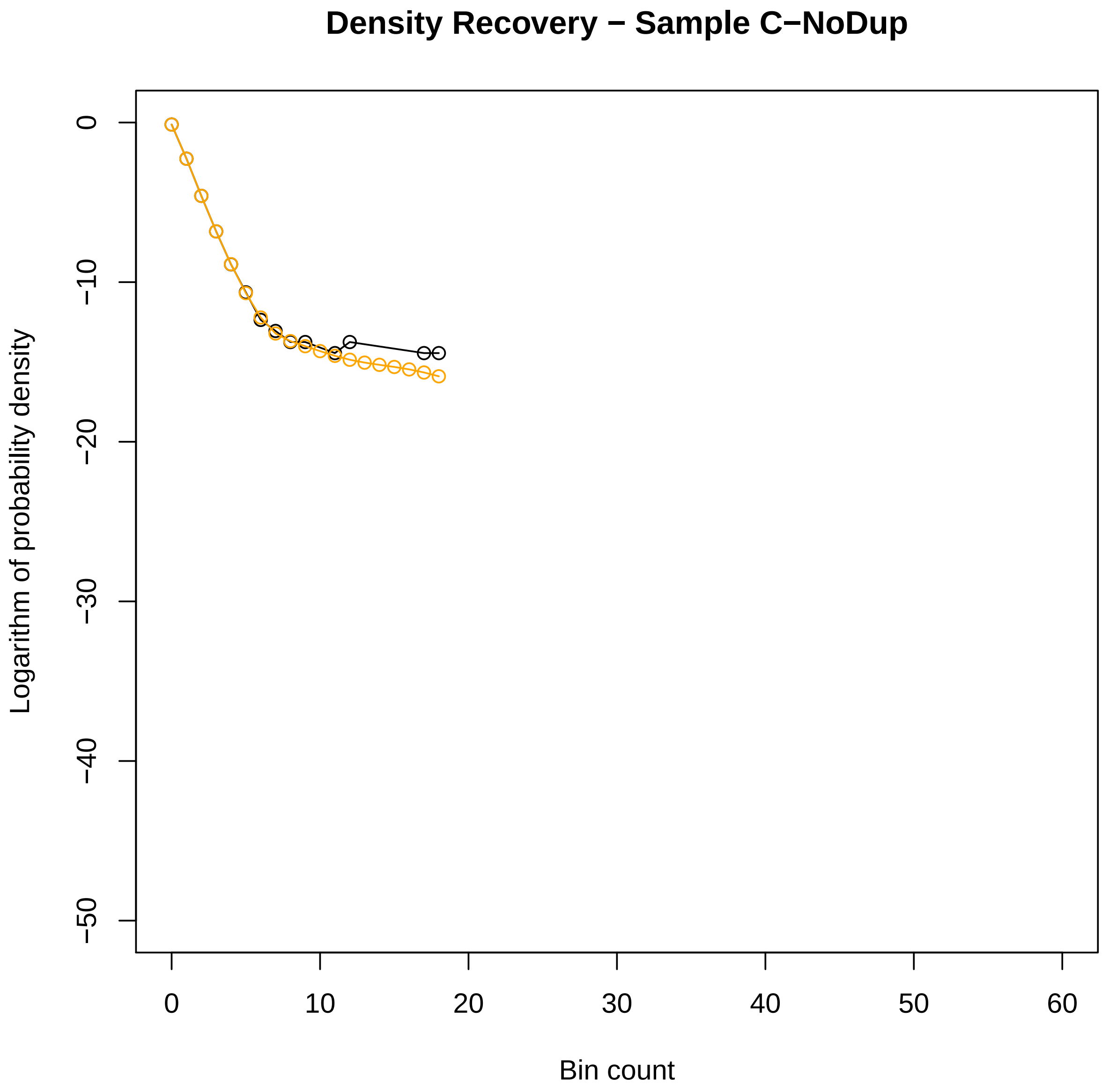
Density Recovery − Sample C−NoDup
Logarithm of probability density
Bin count
0
−10
−20
−30
−40
−50
0
10
20
30
40
50
60

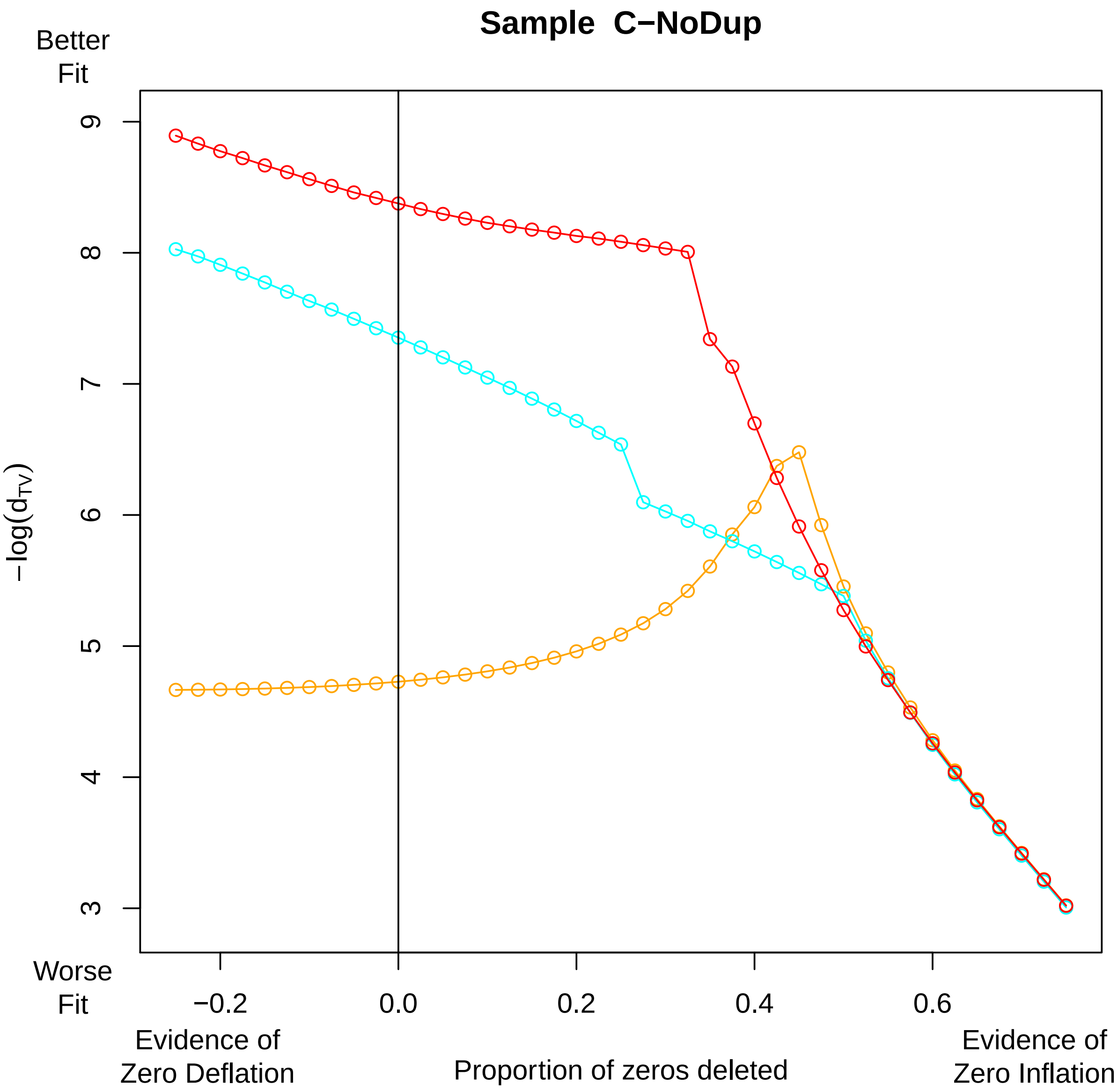
Sample C−NoDup
Better
Fit
Worse
Fit
−log(d_{TV})
9
8
7
6
5
4
3
−0.2
0.0
0.2
0.4
0.6
Evidence of
Zero Deflation
Proportion of zeros deleted
Evidence of
Zero Inflation

Example plots - Sample D, duplicates removed

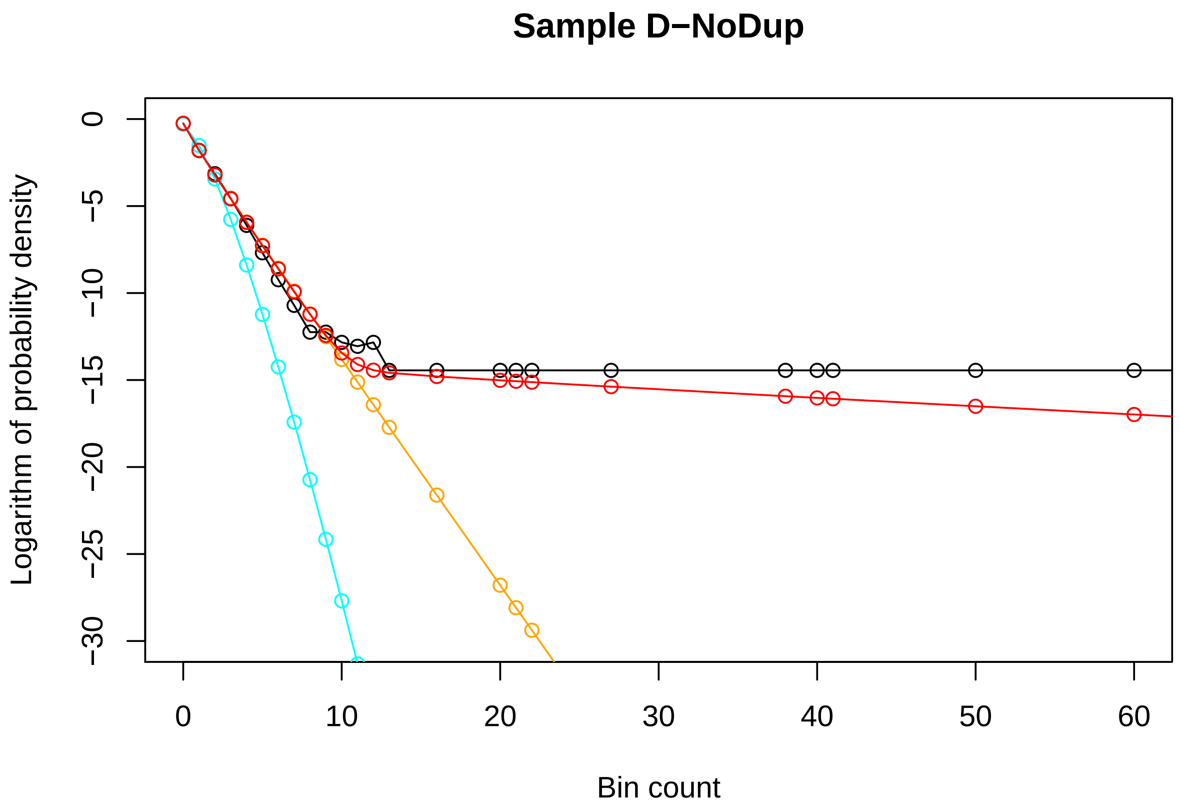

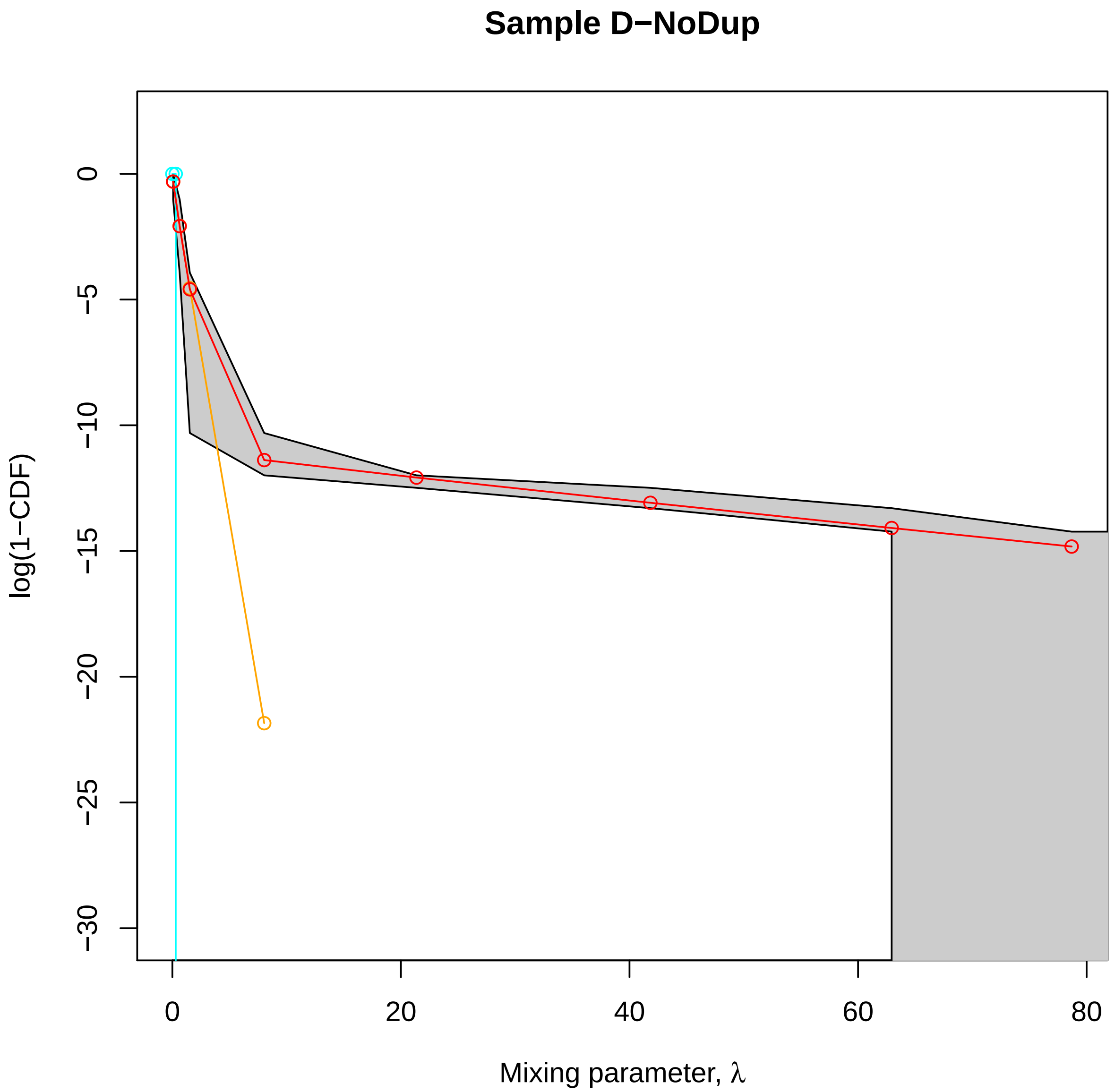

Sample D−NoDup
log(1−CDF)
0
−5
−10
−15
−20
−25
−30
0
20
40
60
80
Mixing parameter, λ

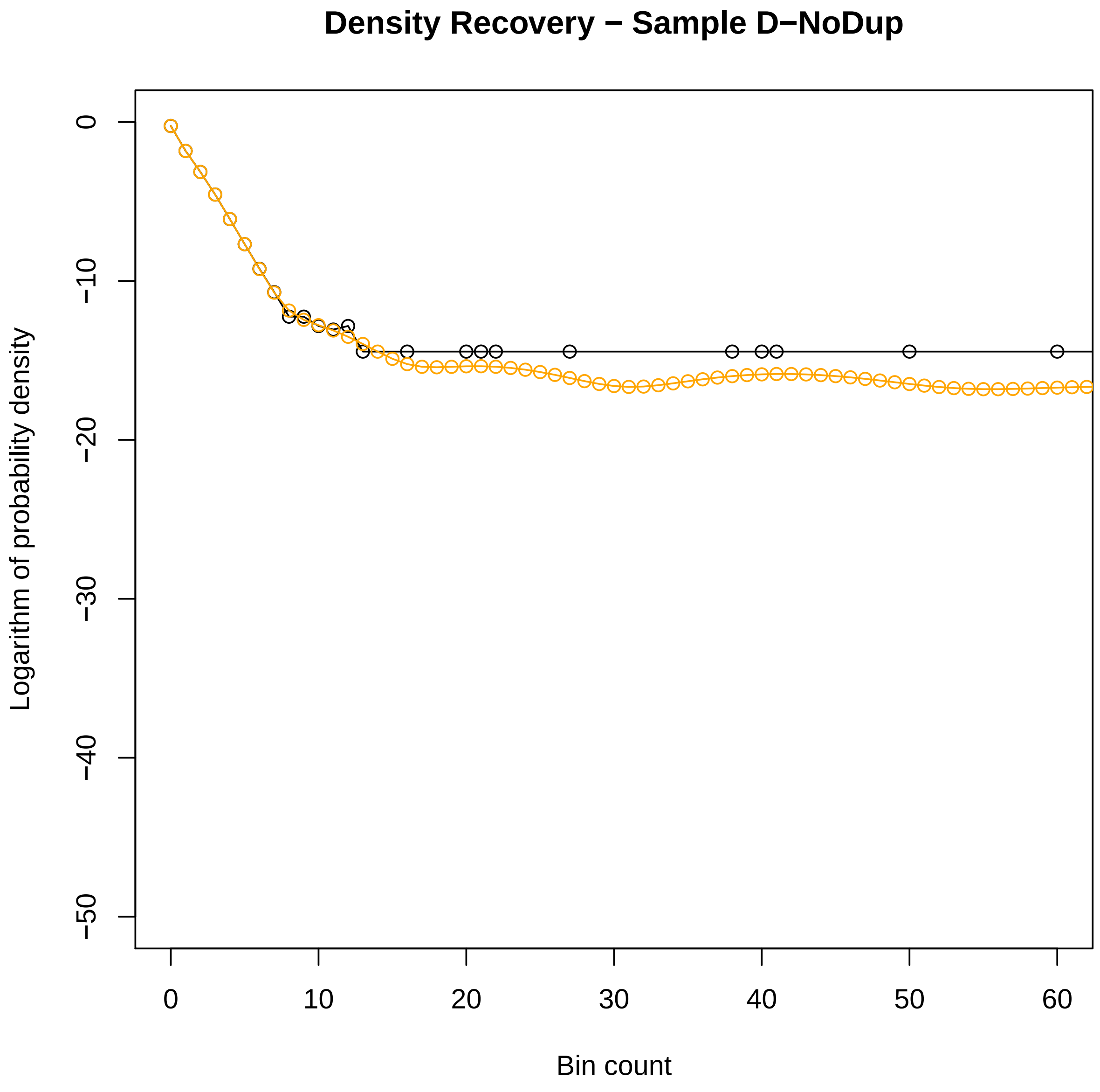

Density Recovery − Sample D−NoDup
Logarithm of probability density
0
−10
−20
−30
−40
−50
0
10
20
30
40
50
60
Bin count

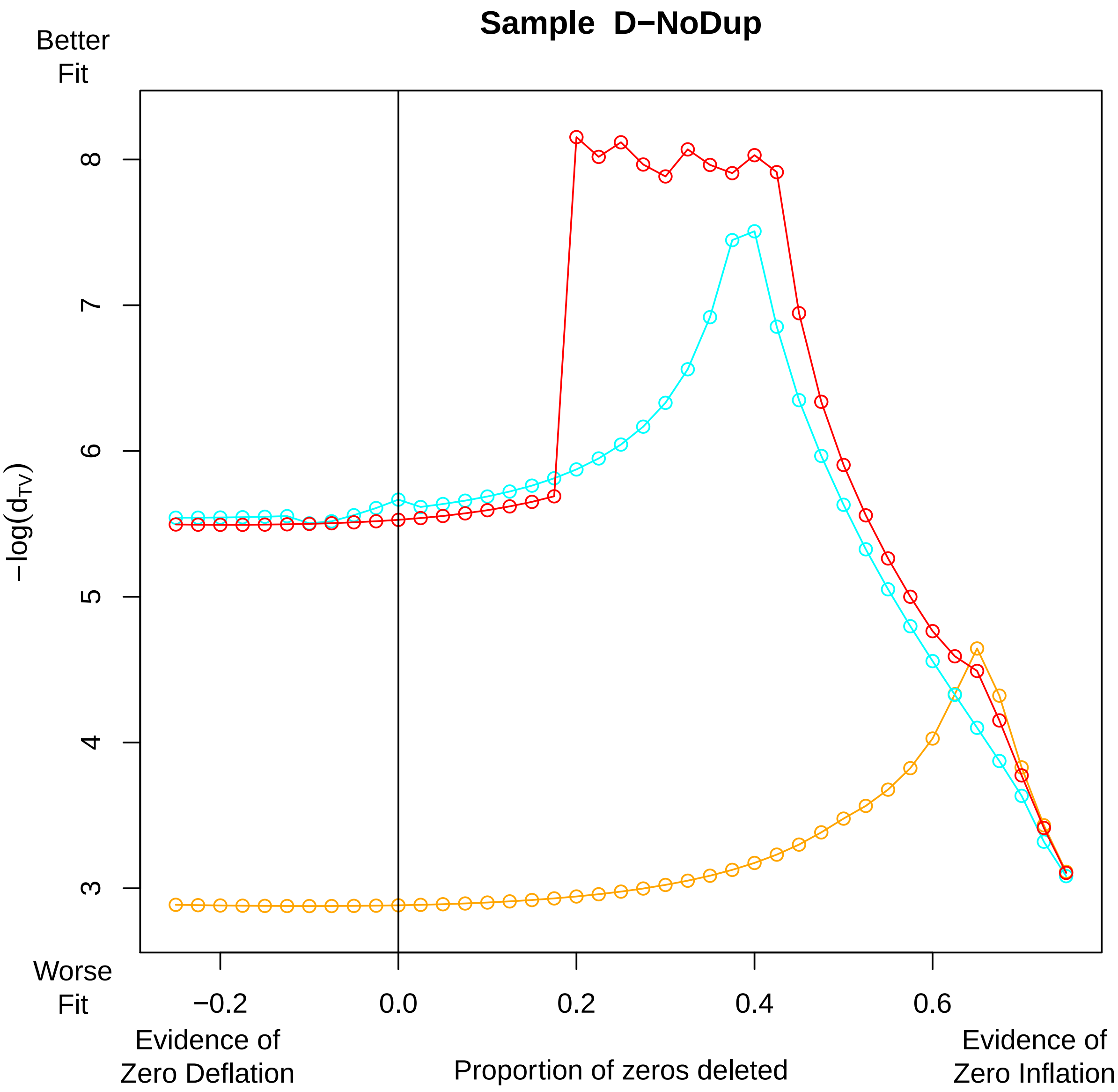
Sample D−NoDup
Better Fit
Worse Fit
−log(dTV)
8
7
6
5
4
3
−0.2
0.0
0.2
0.4
0.6
Evidence of Zero Deflation
Proportion of zeros deleted
Evidence of Zero Inflation
