# Supplementary figures and images for: Quantifying the impact of inter-site heterogeneity on the distribution of ChIP-seq data
Source: Front Genet. 2014 Nov 14;5:399. doi: 10.3389/fgene.2014.00399 (PMC4231950; doi:10.3389/fgene.2014.00399)

# Sample B-NoDup

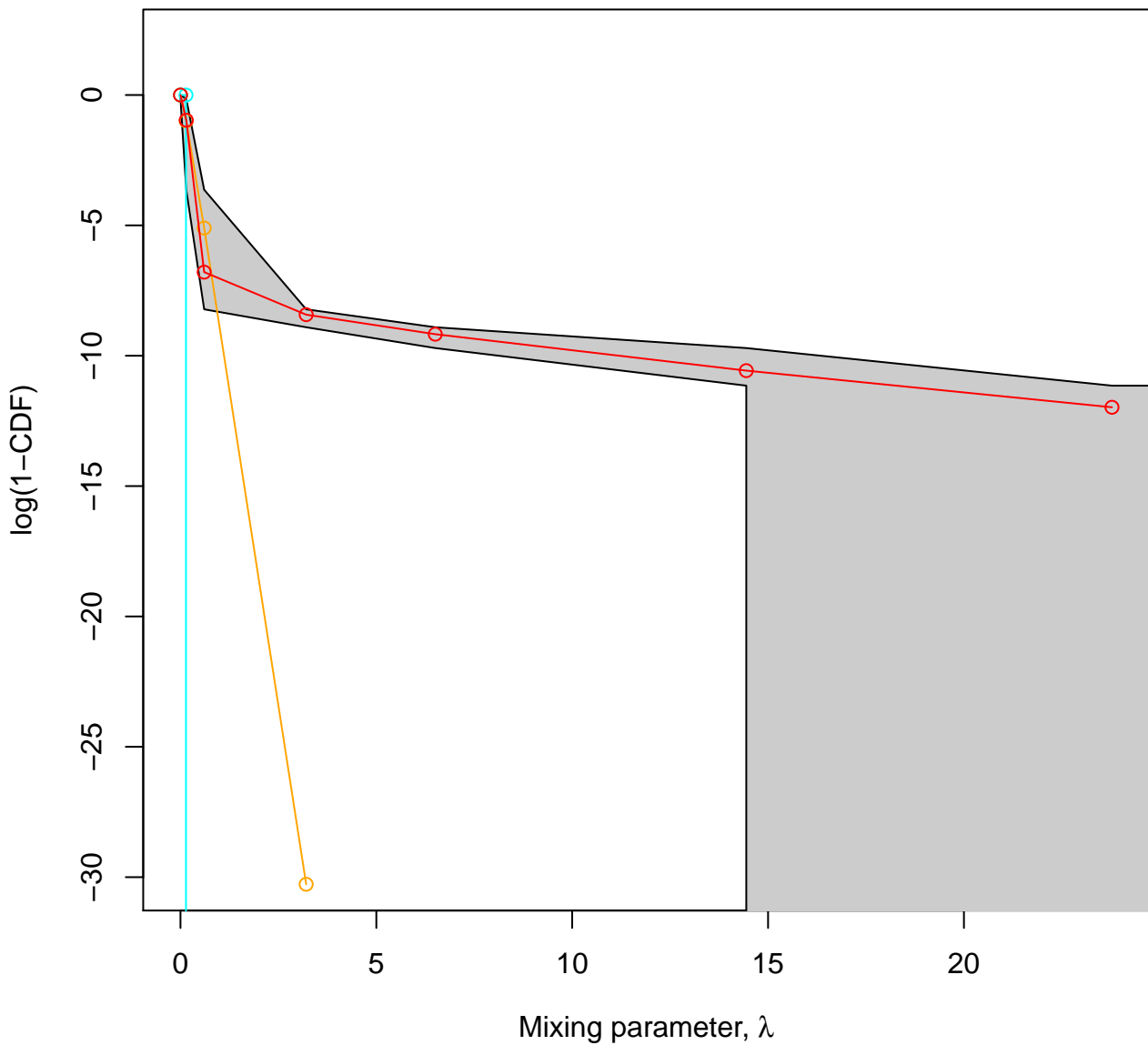

Supplement: Supplementary file 1 [file DataSheet1.ZIP › plots/s10Amix.pdf]

# Sample A-ChIP

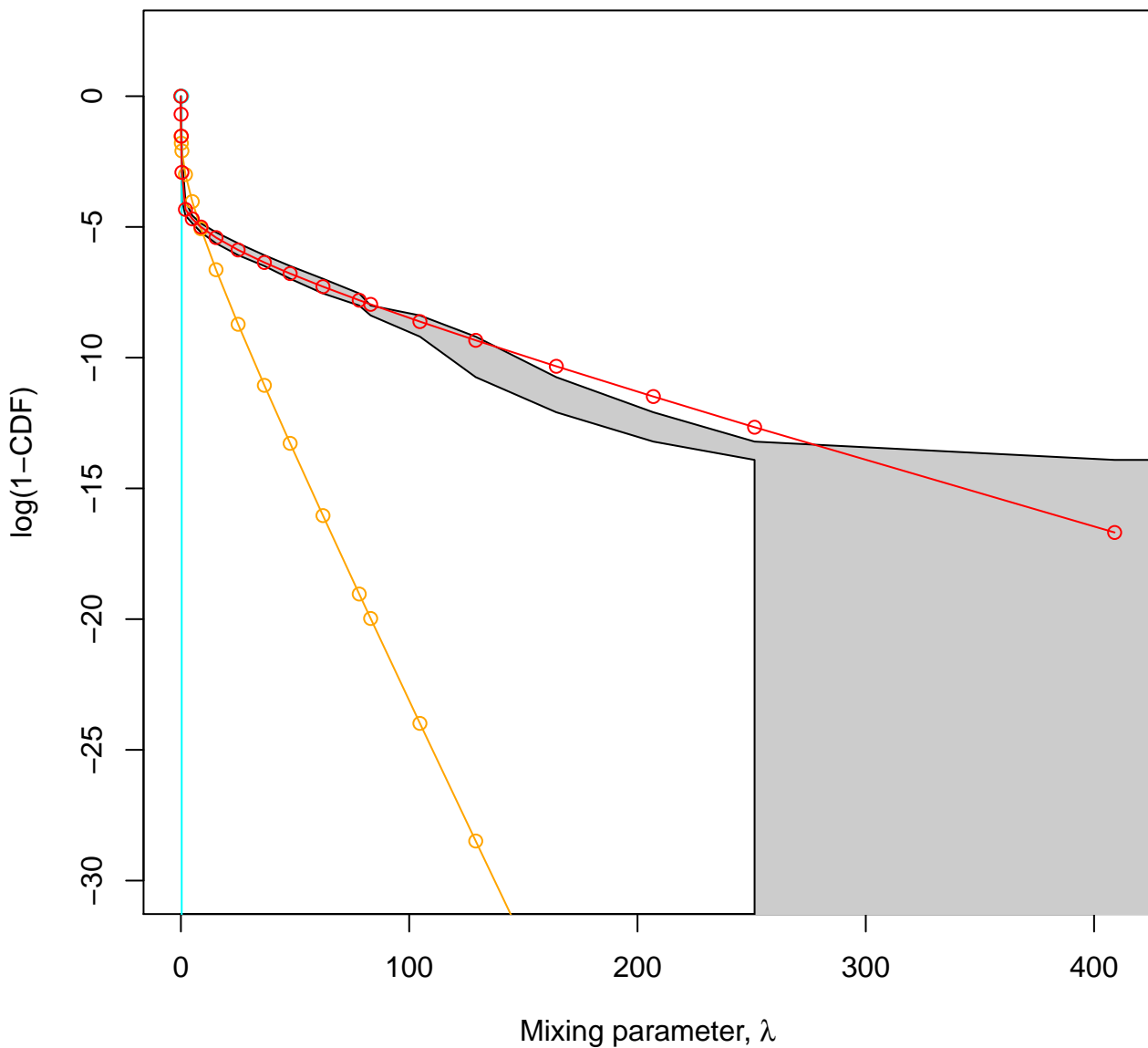

Supplement: Supplementary file 1 [file DataSheet1.ZIP › plots/s5Amix.pdf]

# Sample B-ChIP

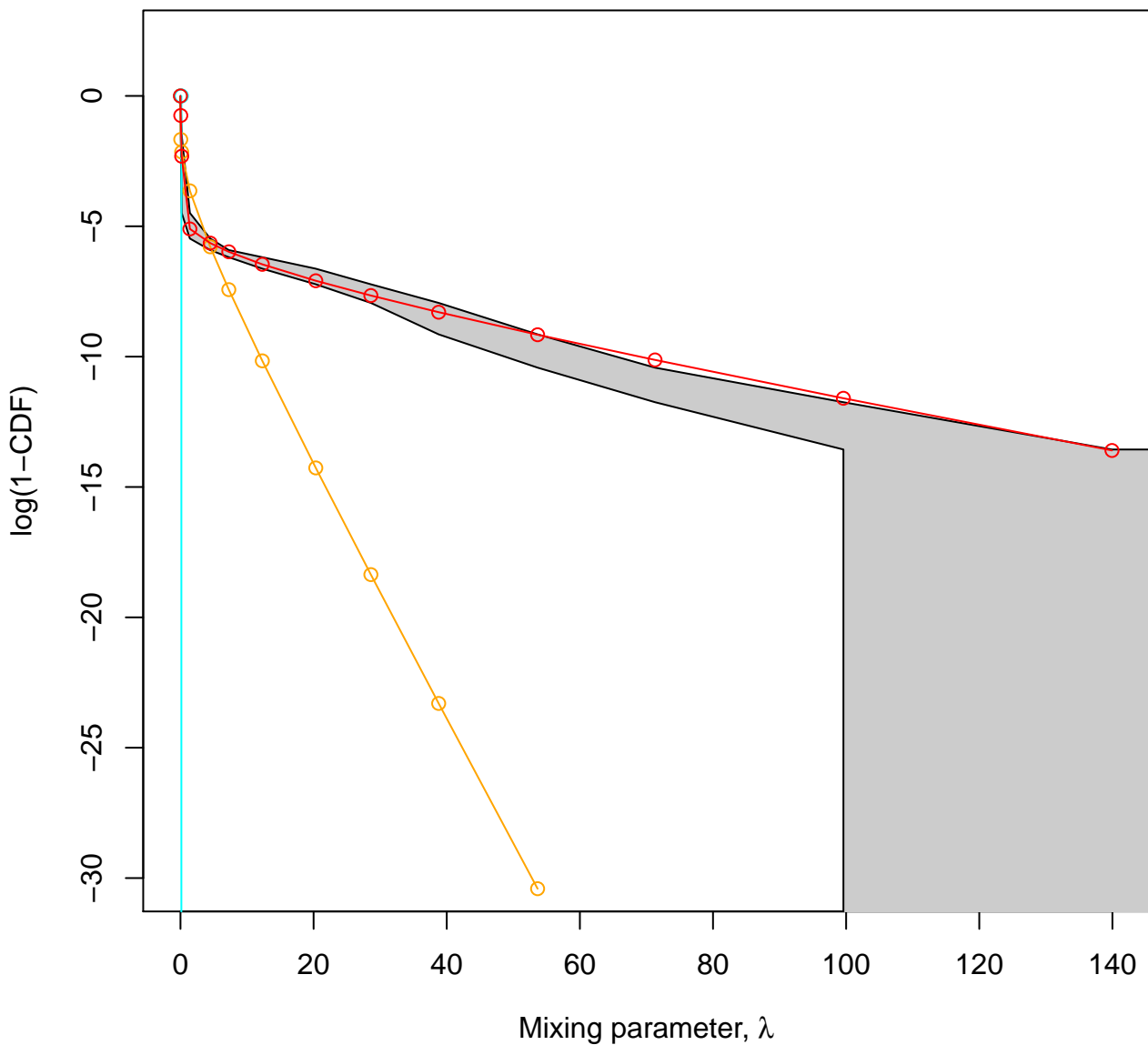

Supplement: Supplementary file 1 [file DataSheet1.ZIP › plots/s6Amix.pdf]

# Sample A

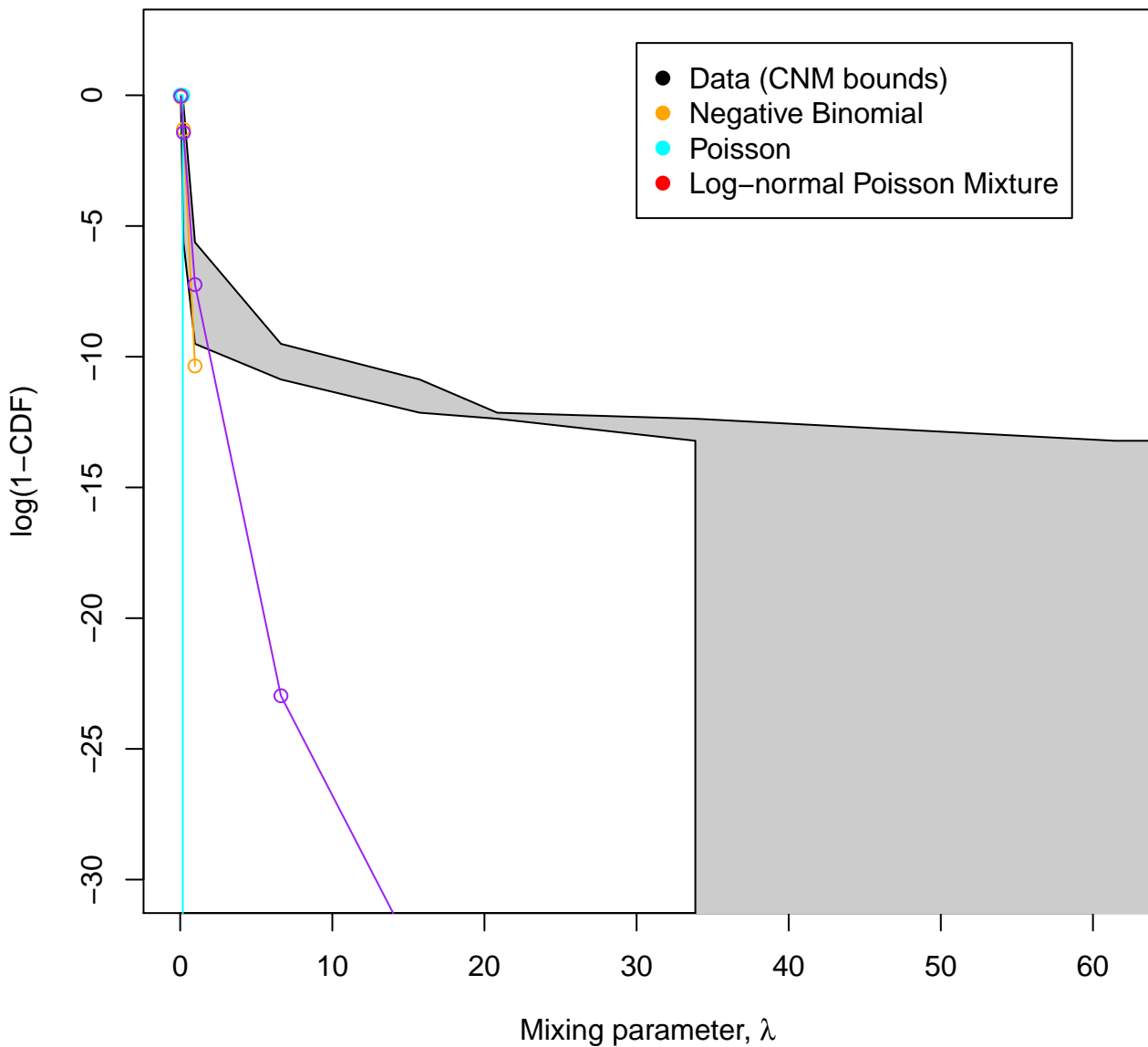

Supplement: Supplementary file 1 [file DataSheet1.ZIP › plots/Prelim2.pdf]

# Sample A-NoDup

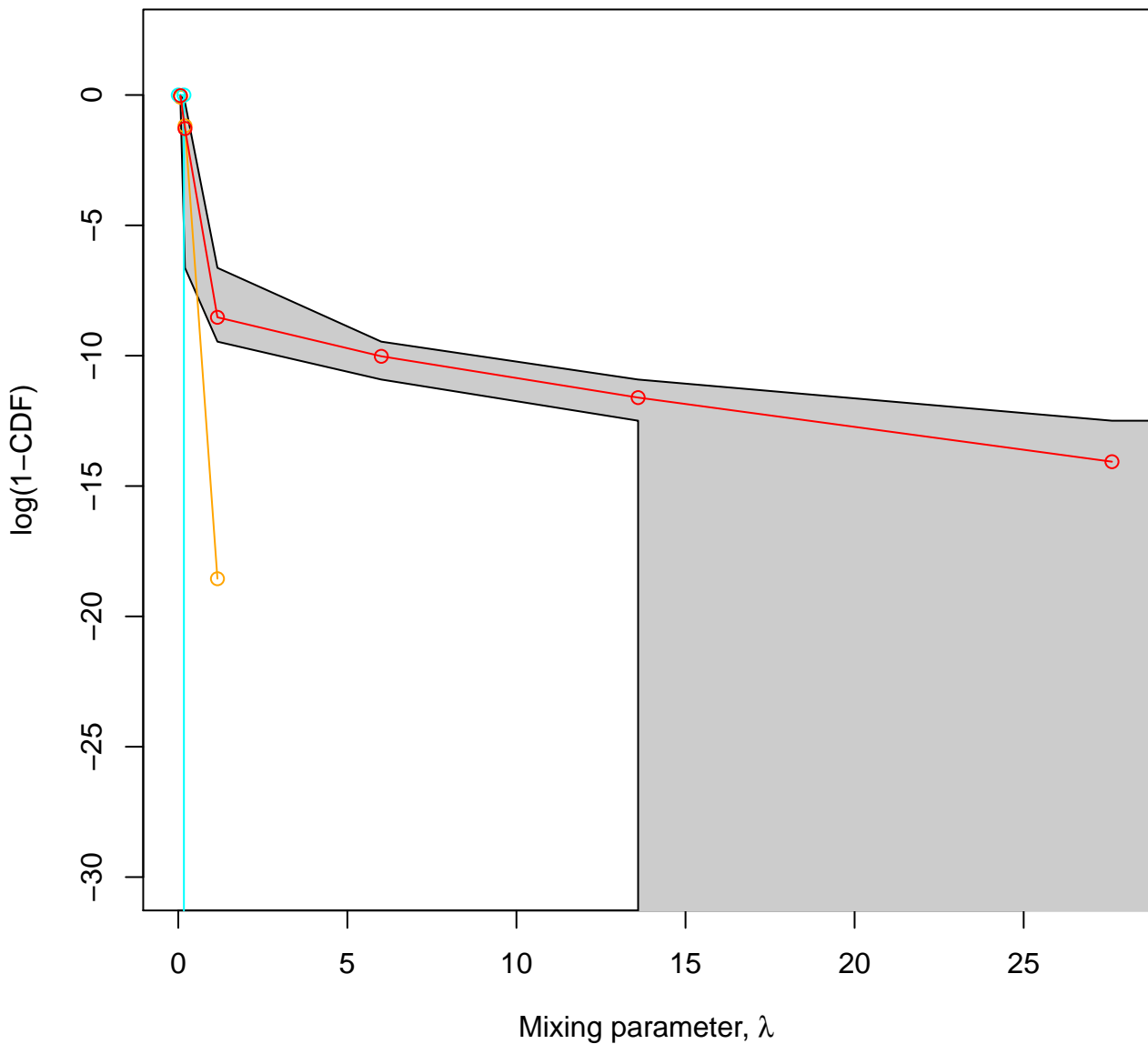

Supplement: Supplementary file 1 [file DataSheet1.ZIP › plots/s9Amix.pdf]

## Sample A

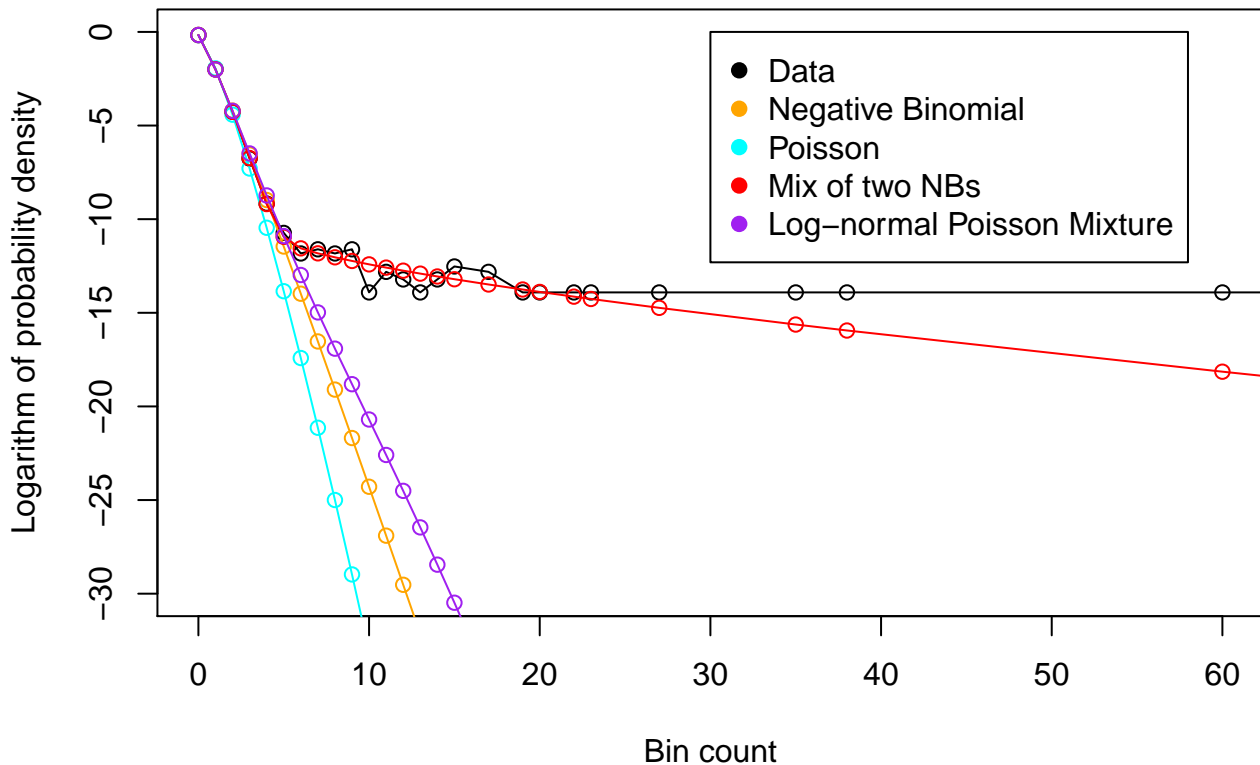

Supplement: Supplementary file 1 [file DataSheet1.ZIP › plots/s1Acount.pdf]

## Sample C-ChIP

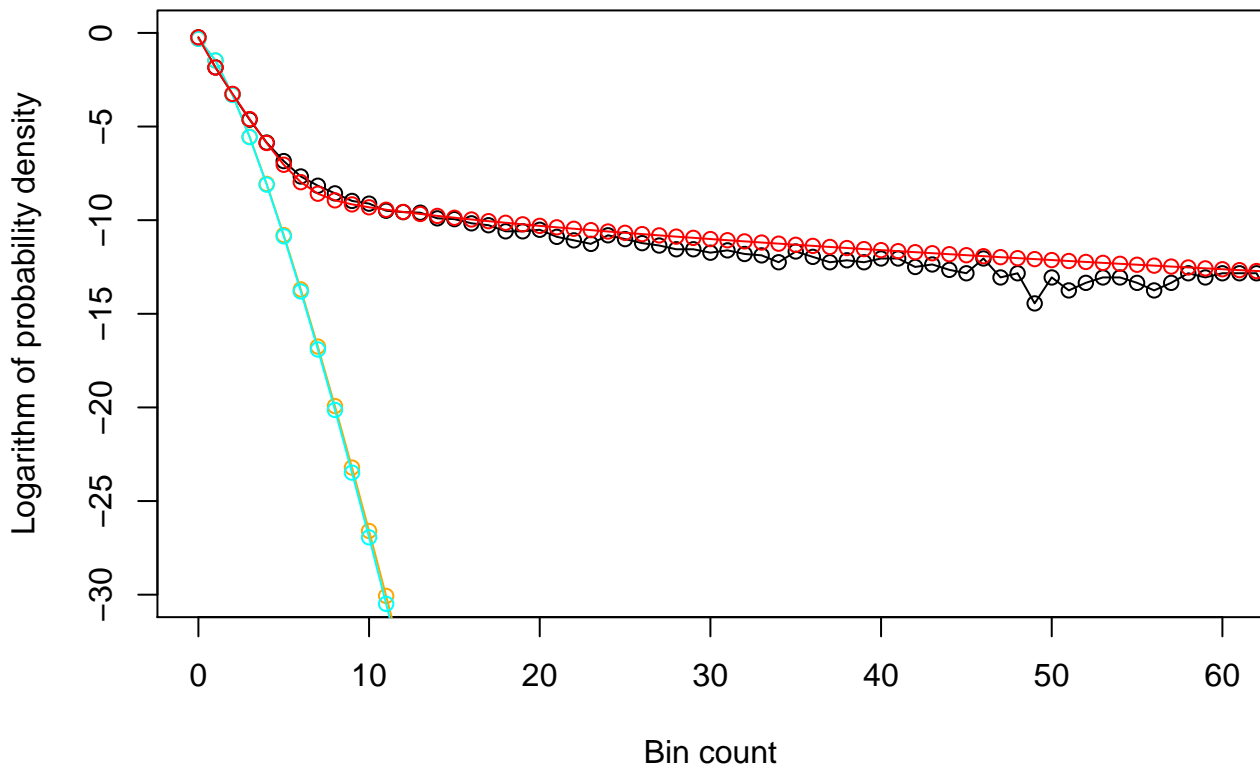

Supplement: Supplementary file 1 [file DataSheet1.ZIP › plots/s7Acount.pdf]

# Sample A-ChIP

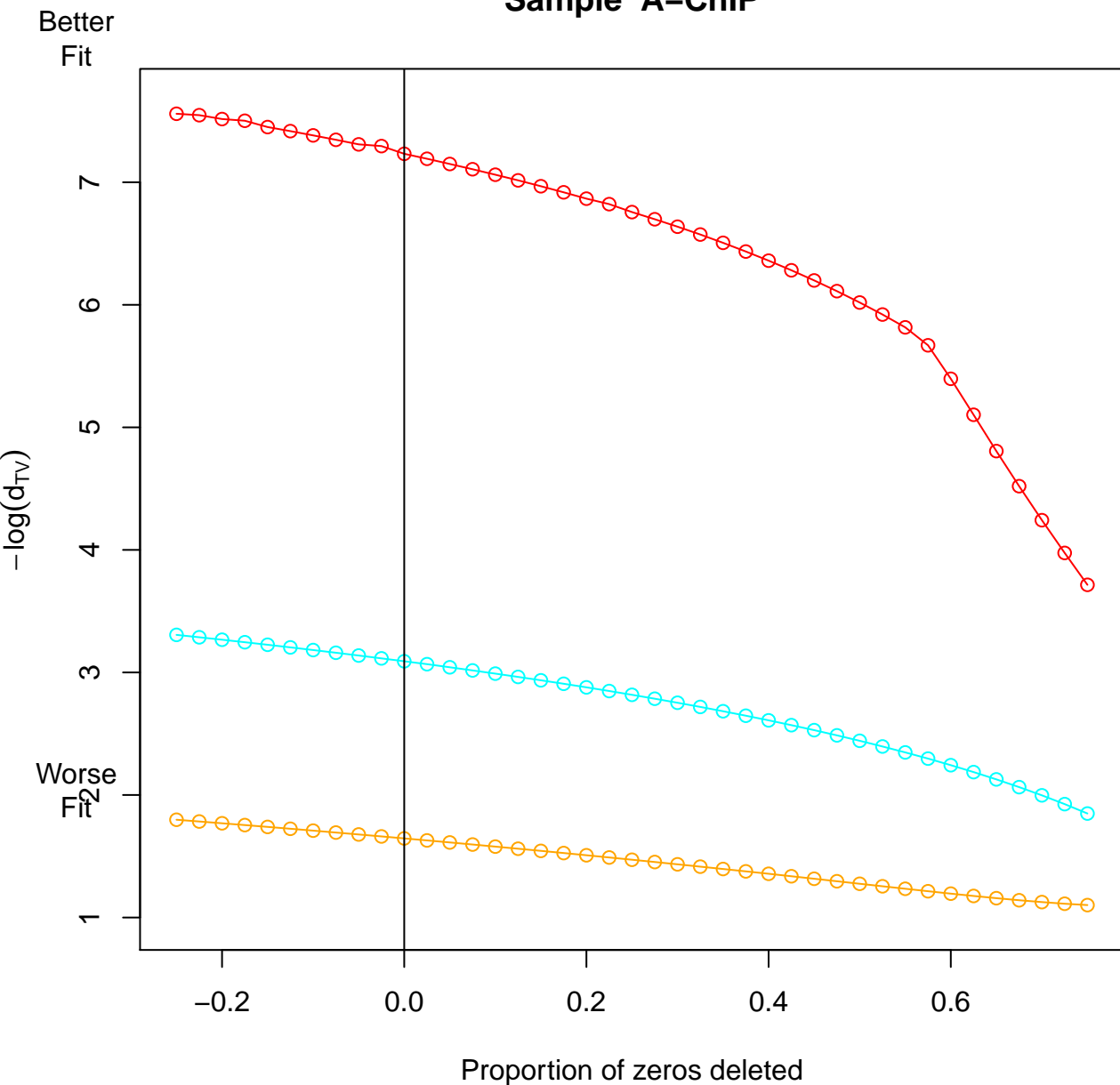

Supplement: Supplementary file 1 [file DataSheet1.ZIP › plots/s5TV.pdf]

# Sample A

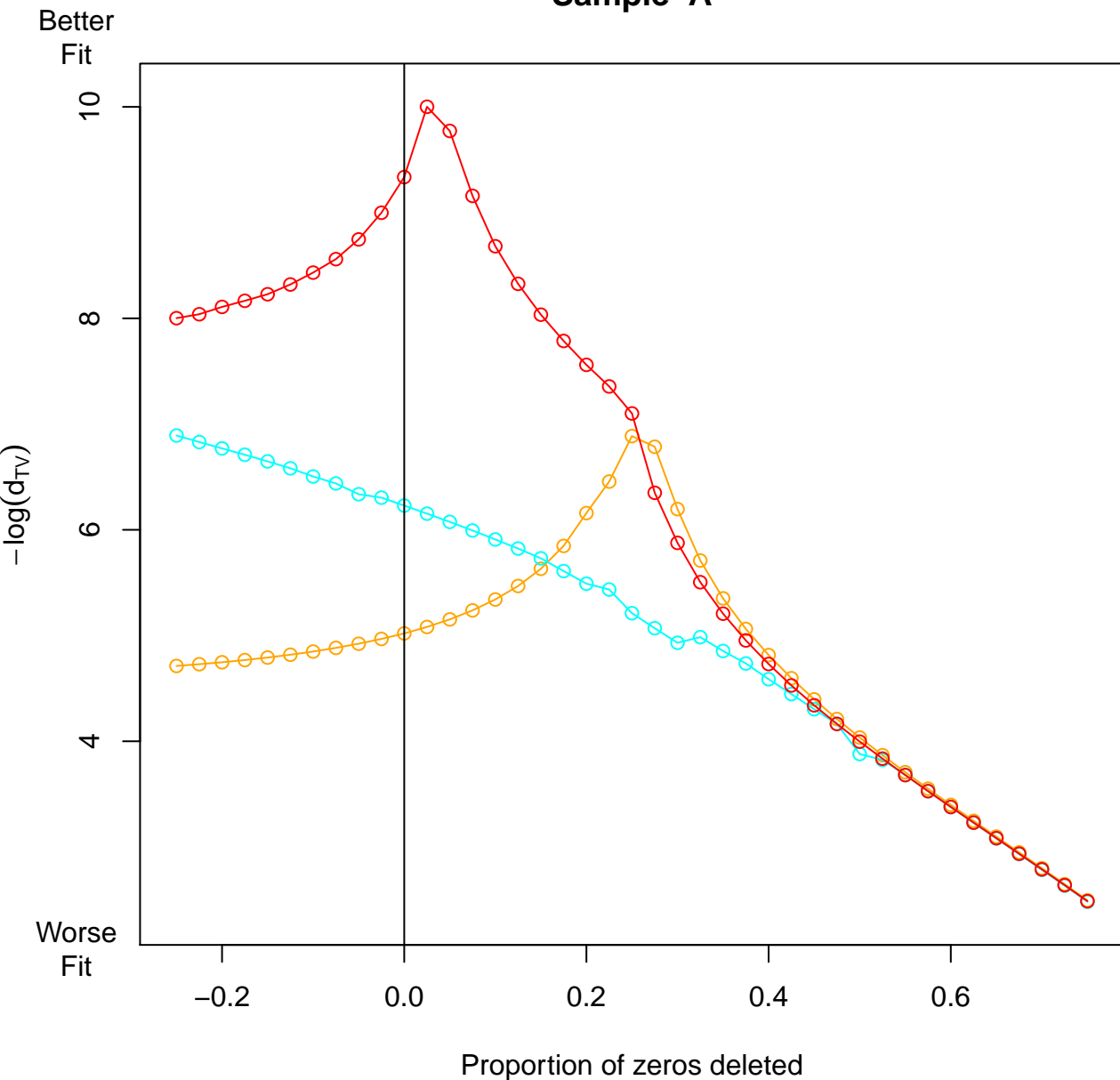

Supplement: Supplementary file 1 [file DataSheet1.ZIP › plots/s1TV.pdf]

Sample C

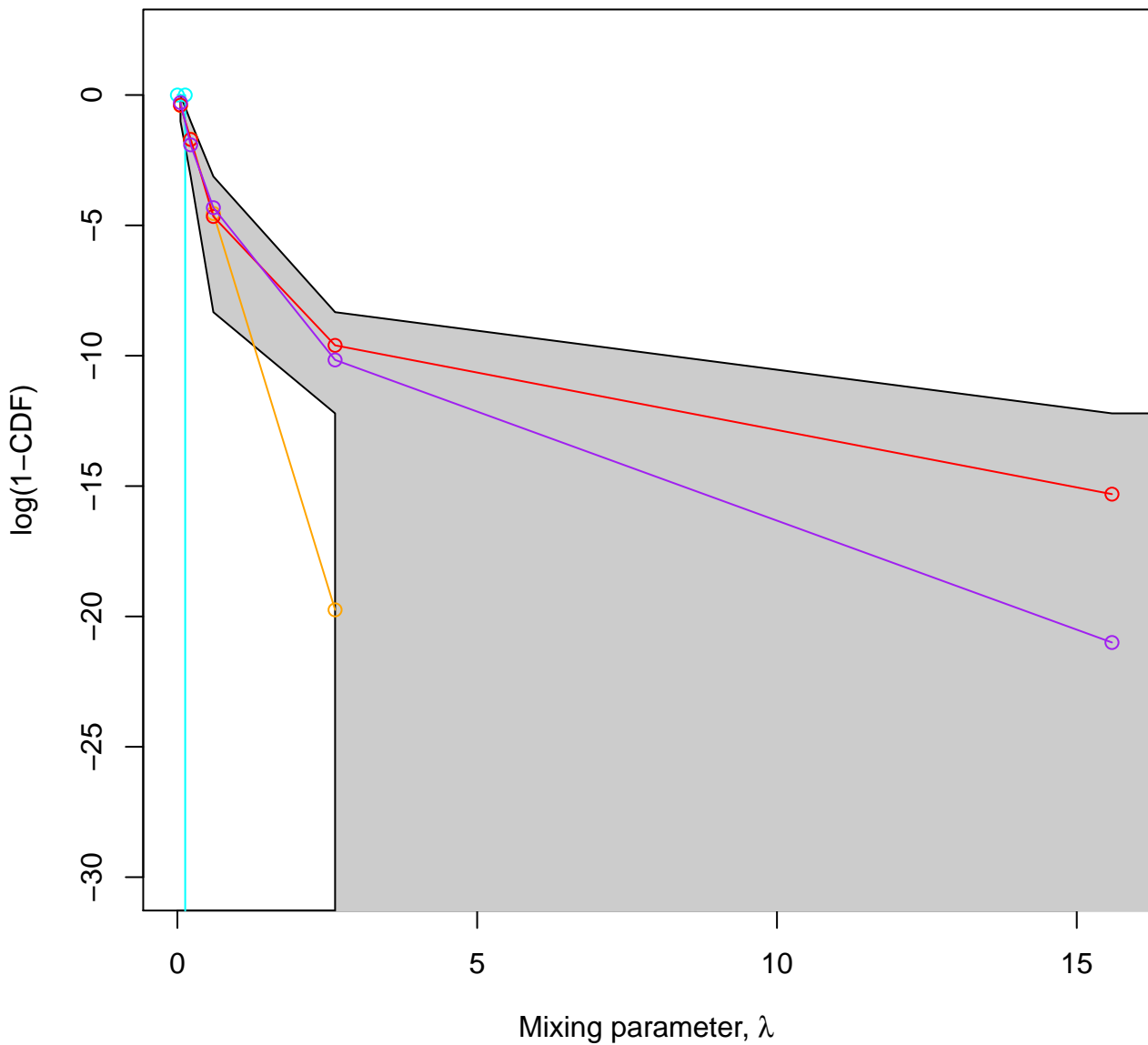

Supplement: Supplementary file 1 [file DataSheet1.ZIP › plots/s3Amix.pdf]

## Sample D-ChIP

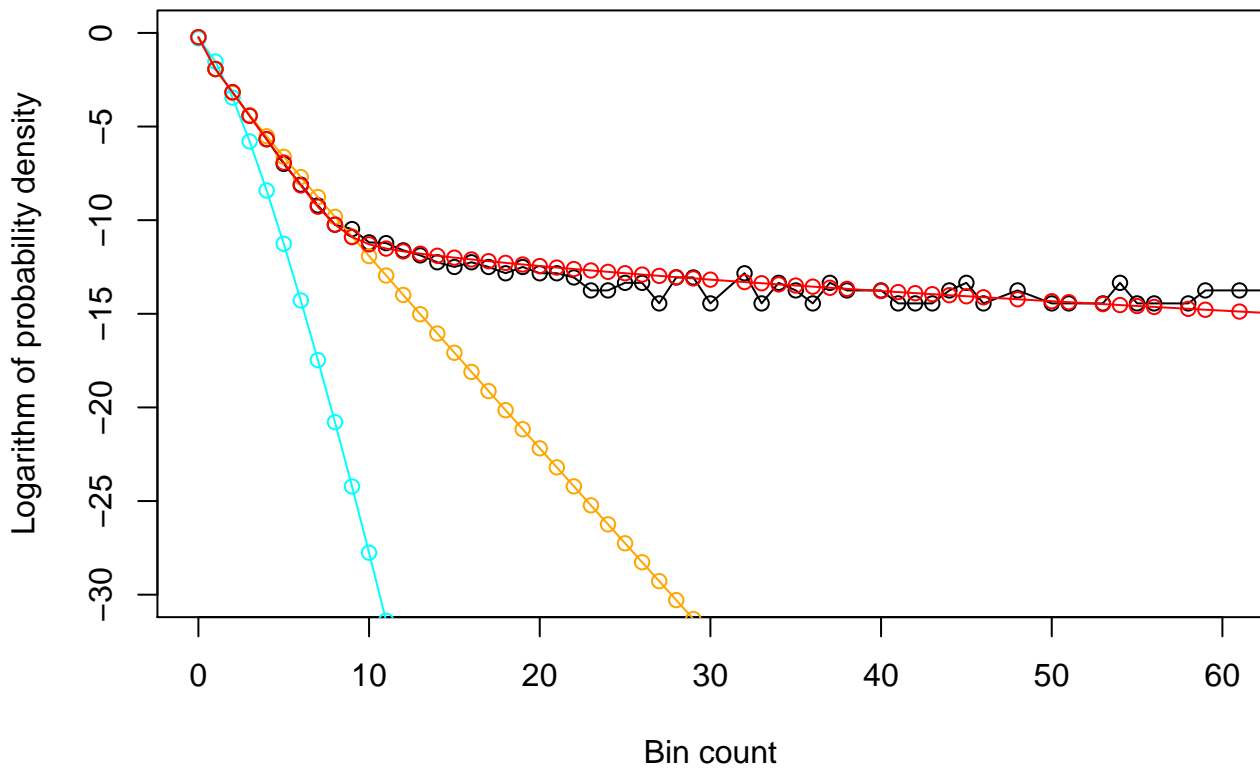

Supplement: Supplementary file 1 [file DataSheet1.ZIP › plots/s8Acount.pdf]

## Density Recovery – Sample D

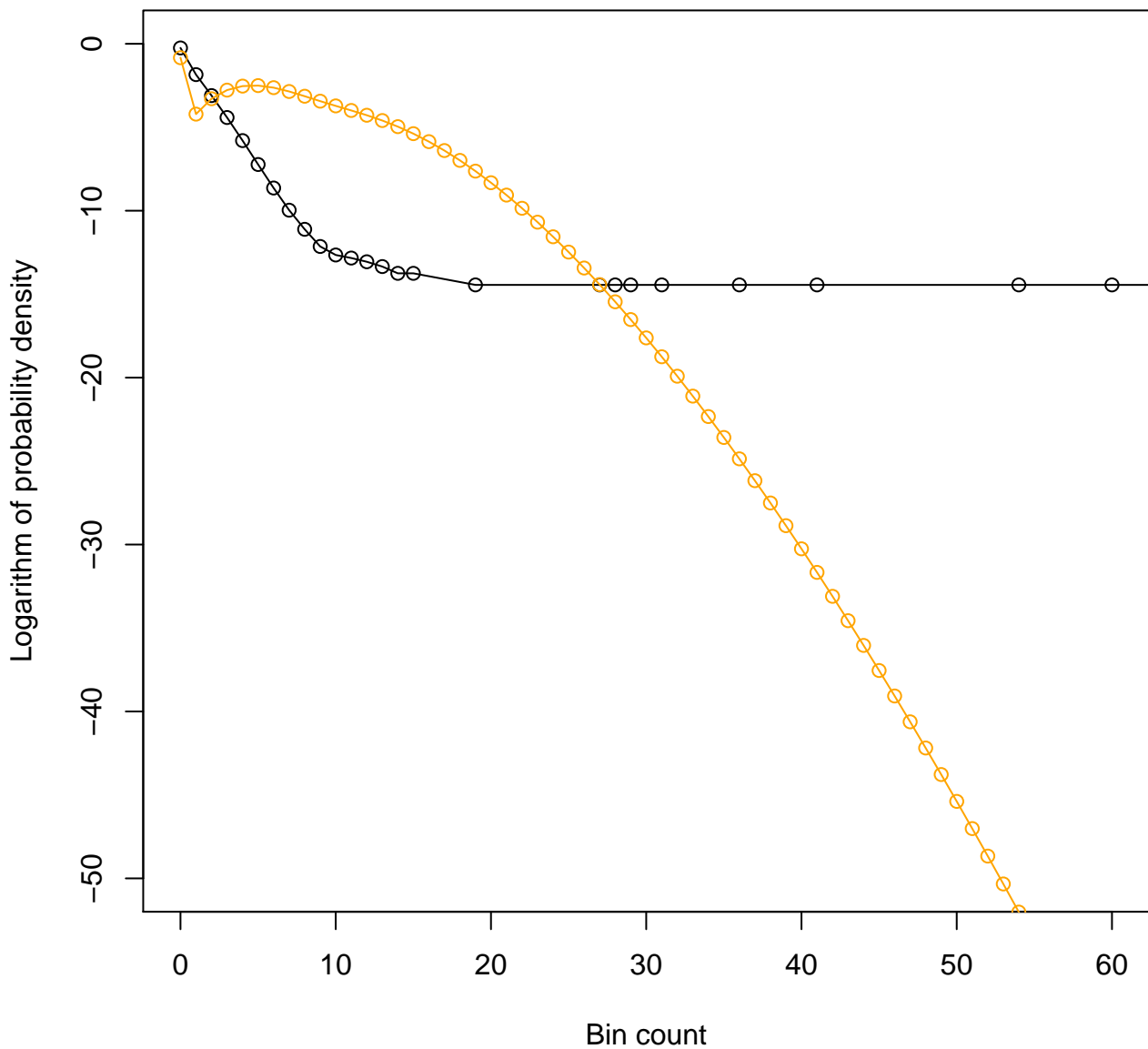

Supplement: Supplementary file 1 [file DataSheet1.ZIP › plots/s4B.pdf]

## Density Recovery – Sample C

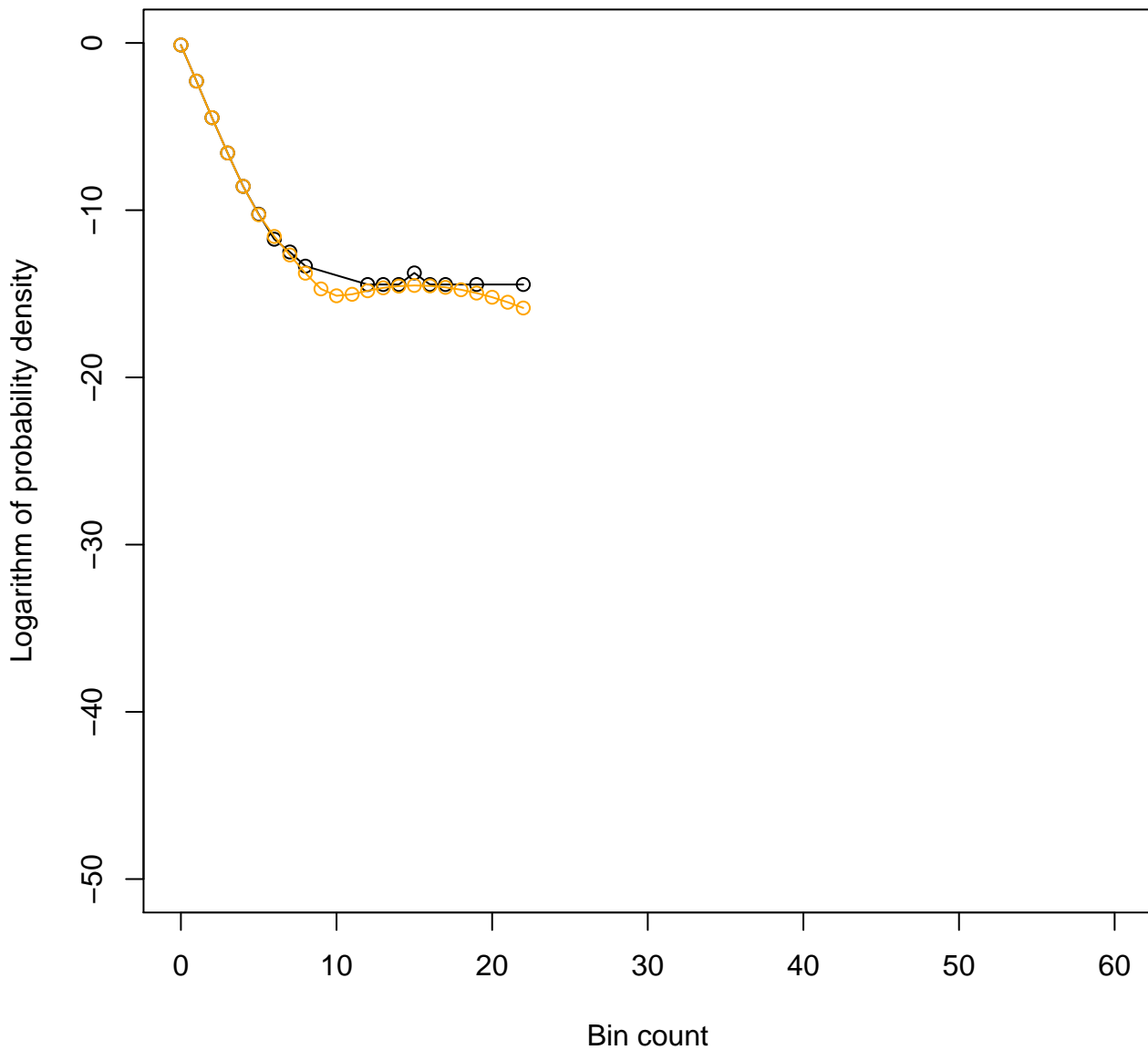

Supplement: Supplementary file 1 [file DataSheet1.ZIP › plots/s3B.pdf]

## Count Distribution – Sample A

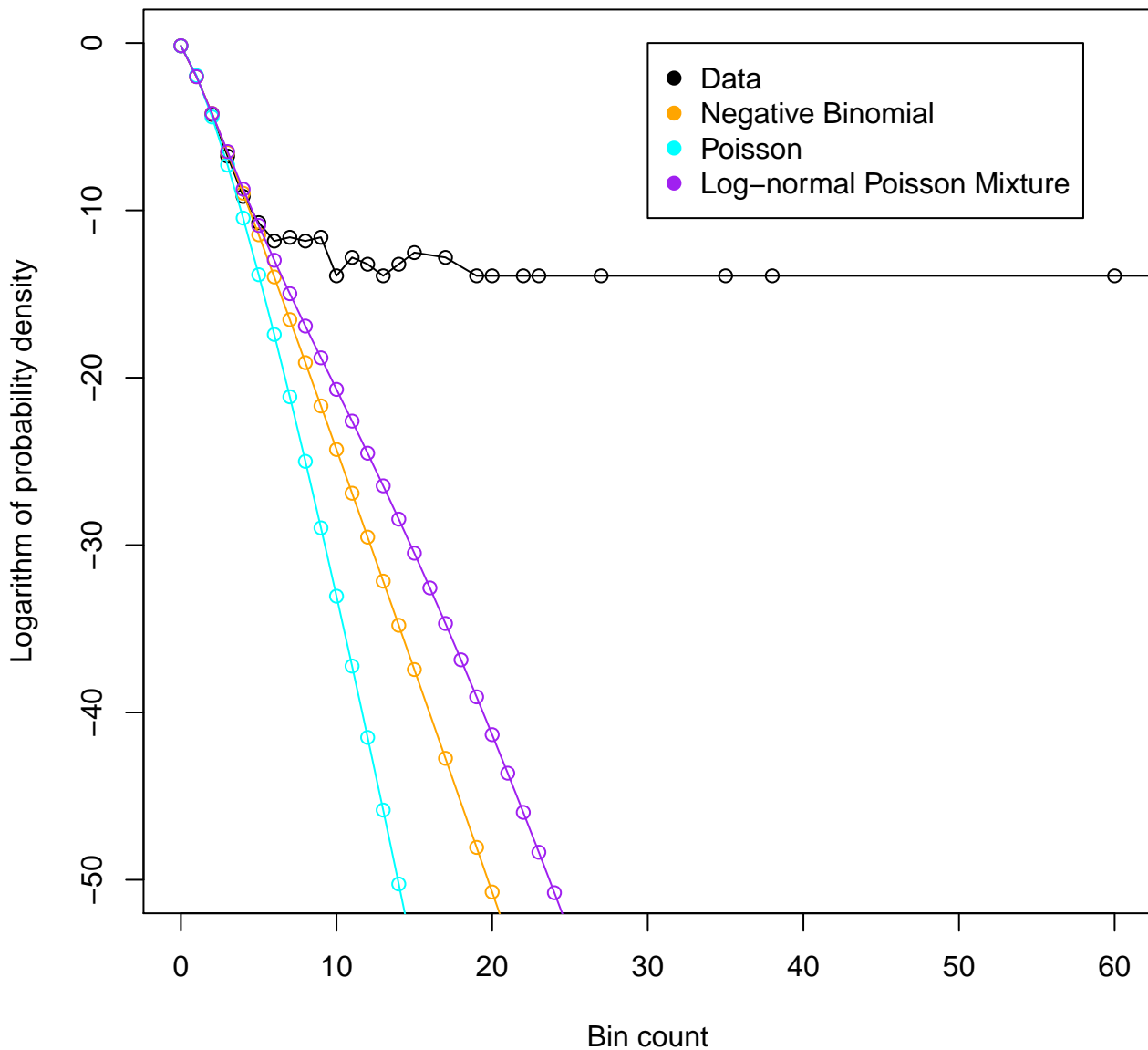

Supplement: Supplementary file 1 [file DataSheet1.ZIP › plots/Prelim.pdf]

## Sample C

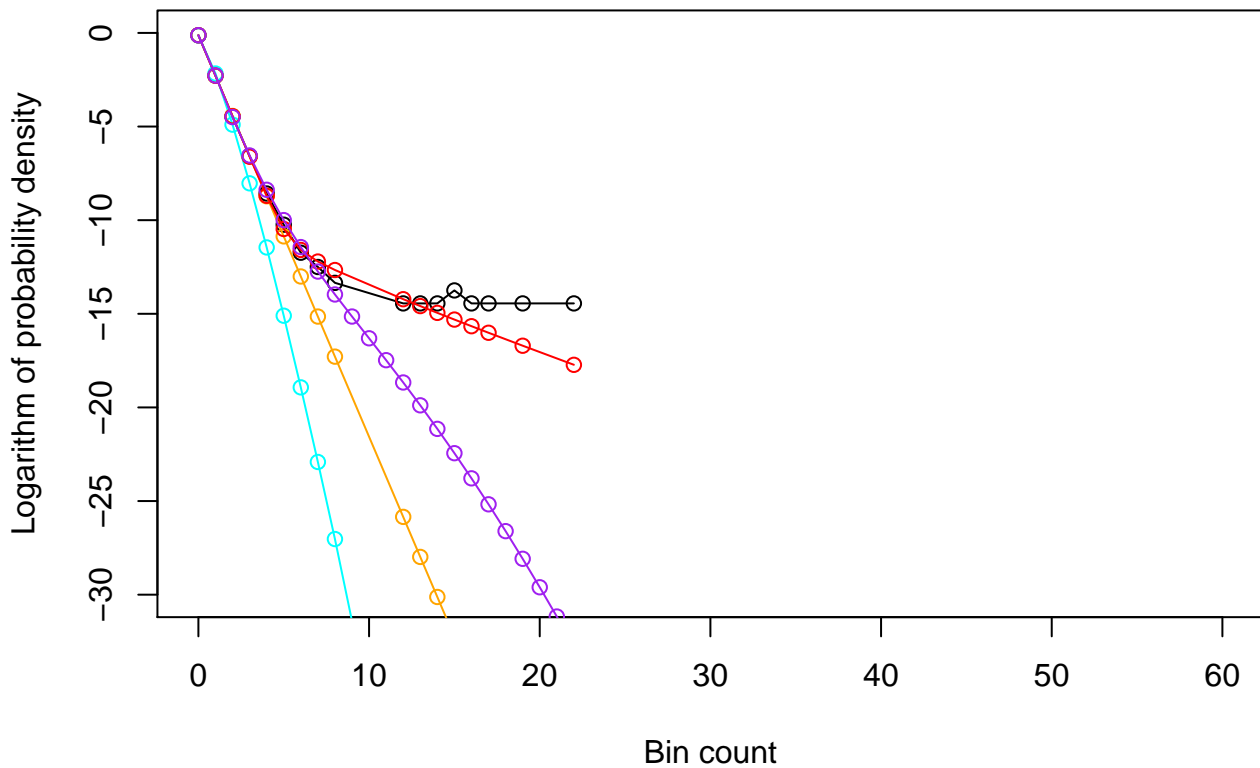

Supplement: Supplementary file 1 [file DataSheet1.ZIP › plots/s3Acount.pdf]

# Sample D-ChIP

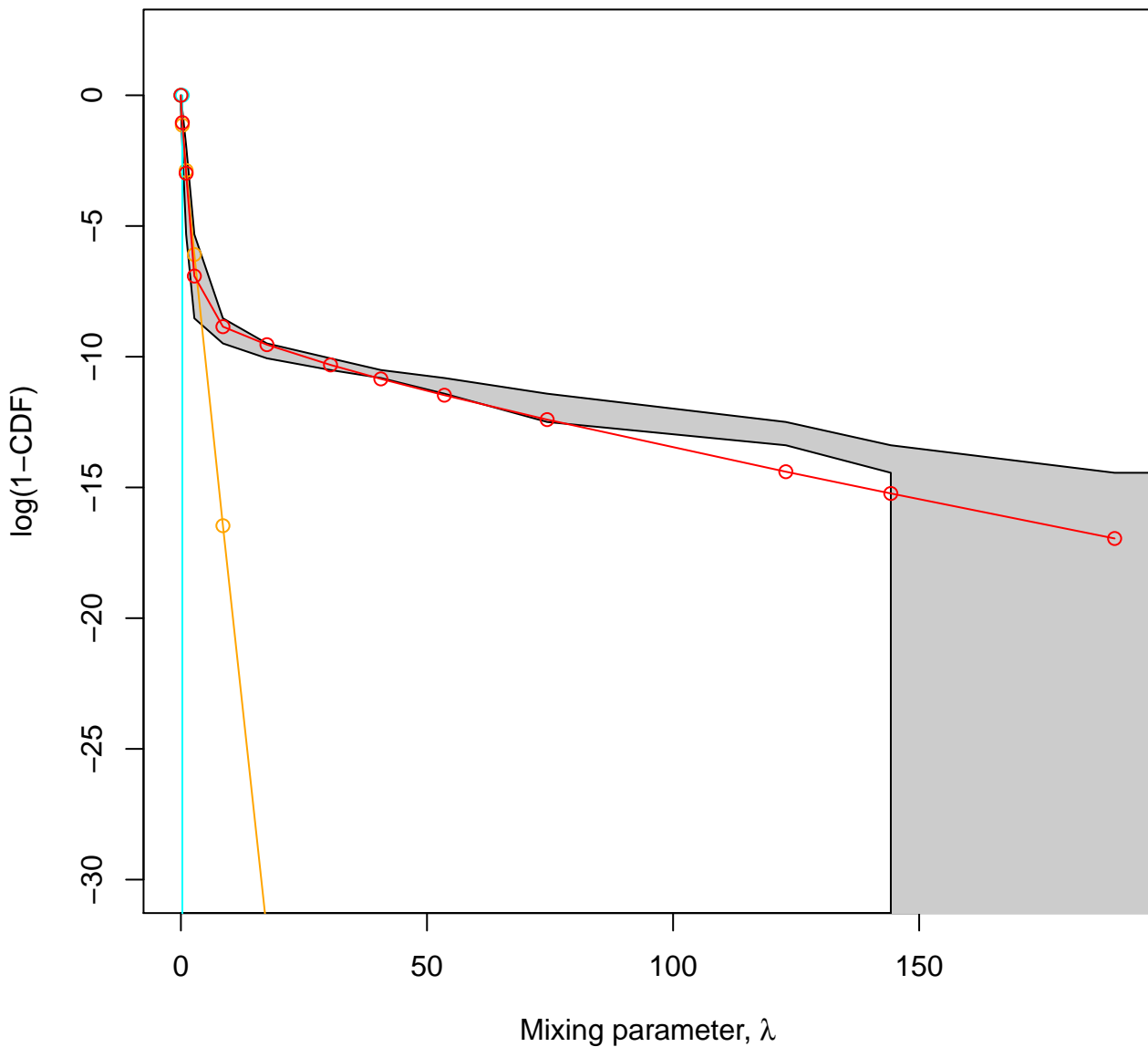

Supplement: Supplementary file 1 [file DataSheet1.ZIP › plots/s8Amix.pdf]

## Density Recovery – Sample B–ChIP

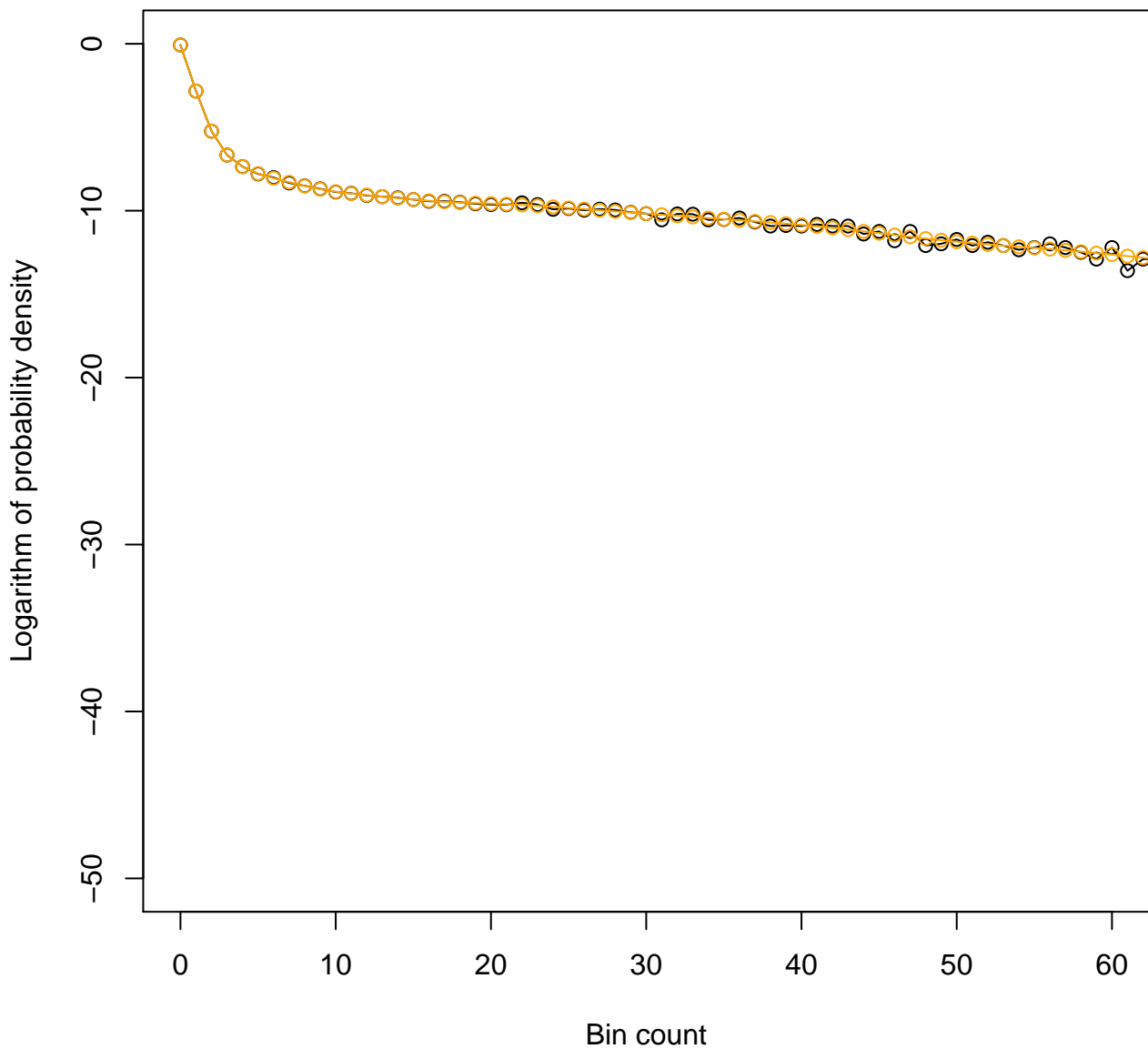

Supplement: Supplementary file 1 [file DataSheet1.ZIP › plots/s6B.pdf]

# Sample C-NoDup

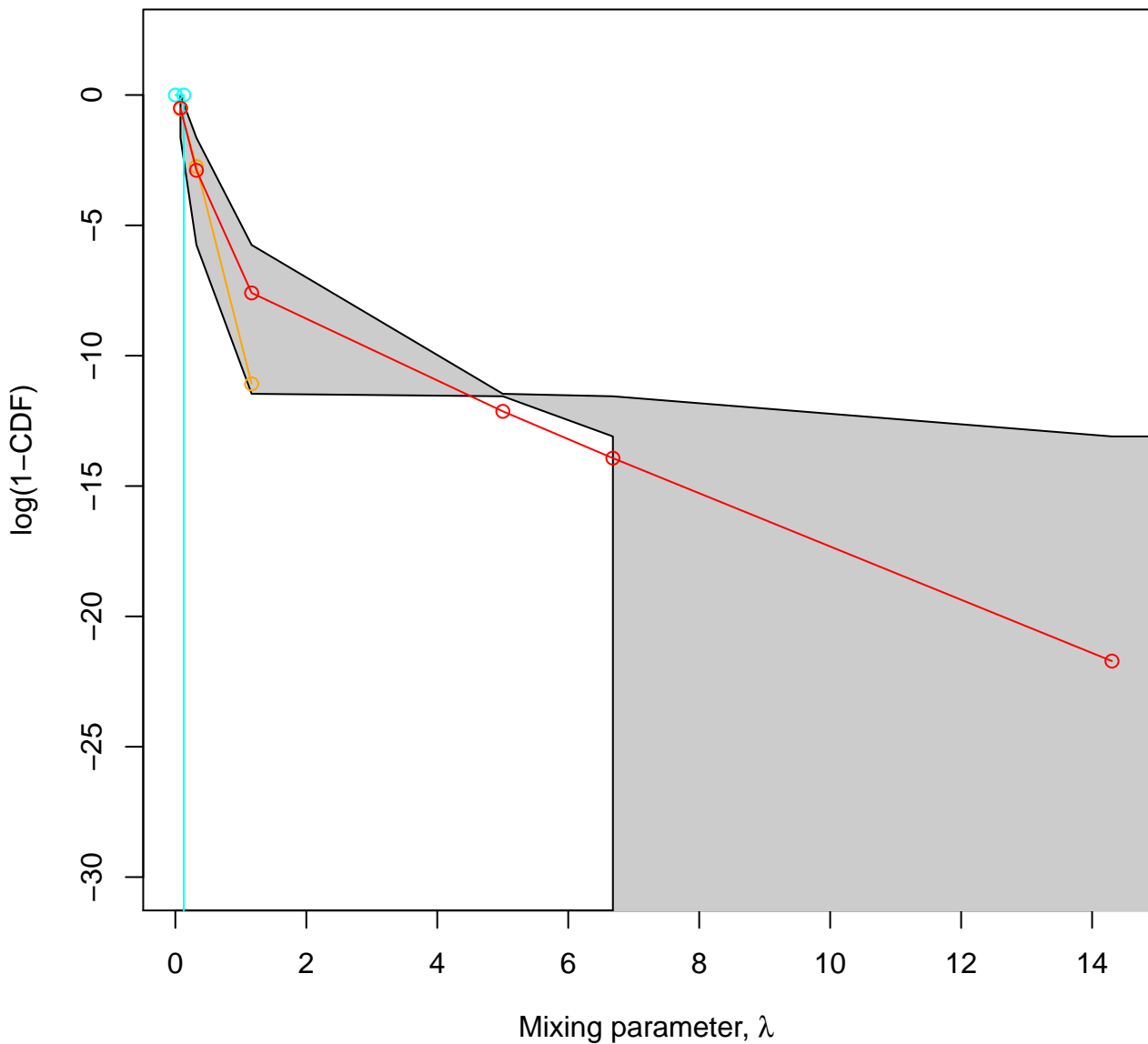

Supplement: Supplementary file 1 [file DataSheet1.ZIP › plots/s11Amix.pdf]

# Sample D-ChIP

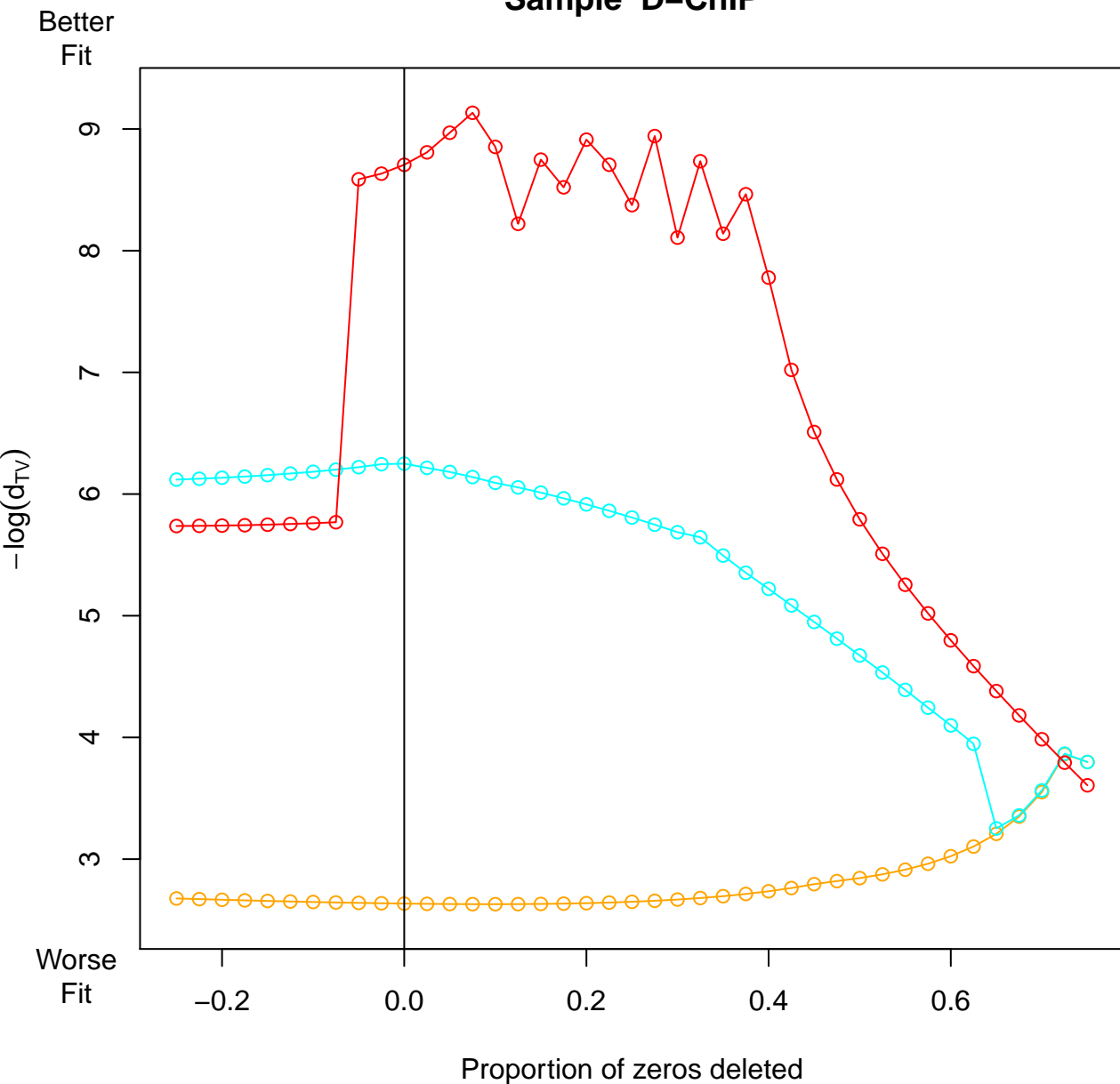

Supplement: Supplementary file 1 [file DataSheet1.ZIP › plots/s8TV.pdf]

**Sample B**

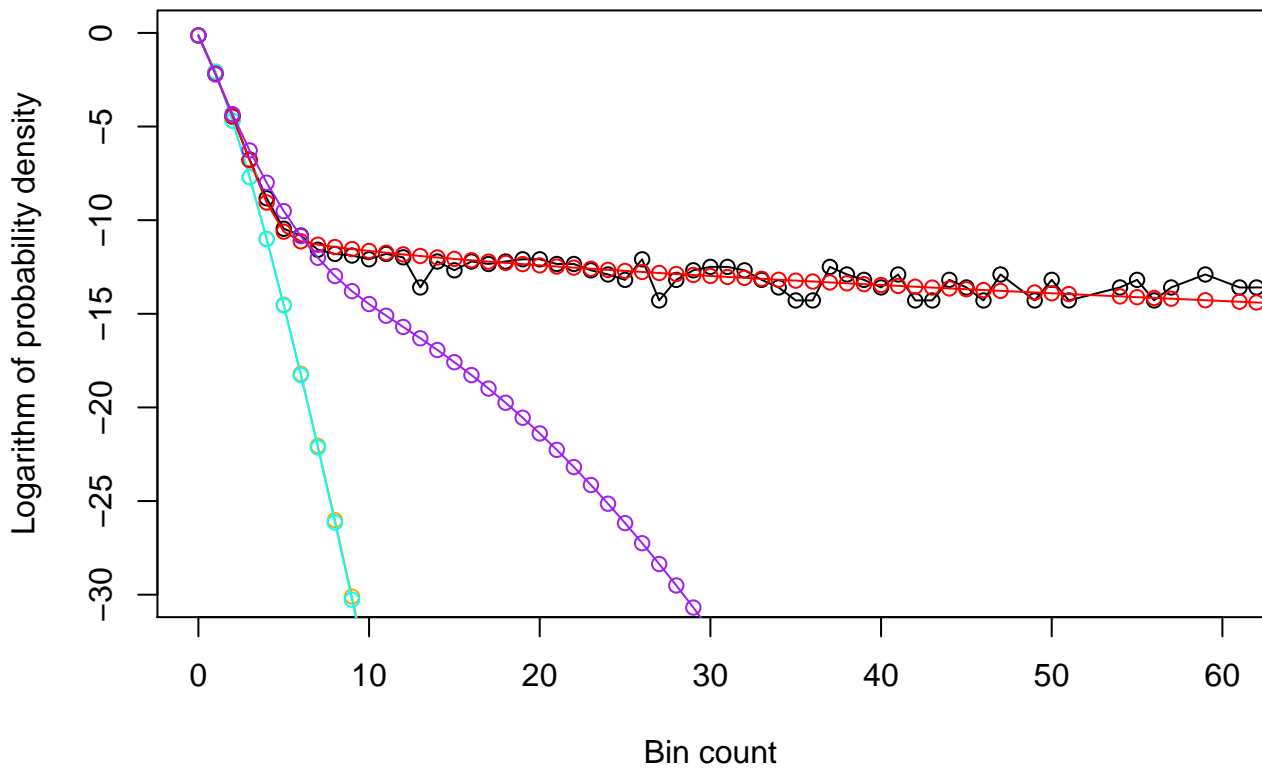

Supplement: Supplementary file 1 [file DataSheet1.ZIP › plots/s2Acount.pdf]

# Total variation for each distribution

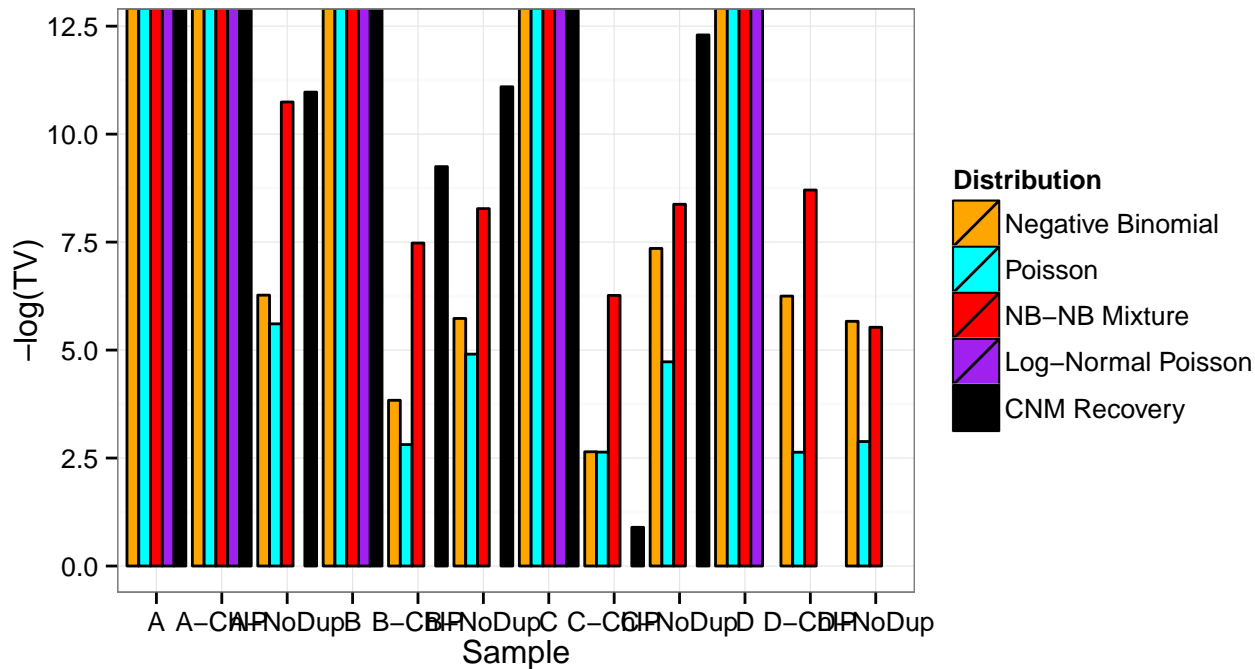

Supplement: Supplementary file 1 [file DataSheet1.ZIP › plots/TVfull.pdf]

**Sample D**

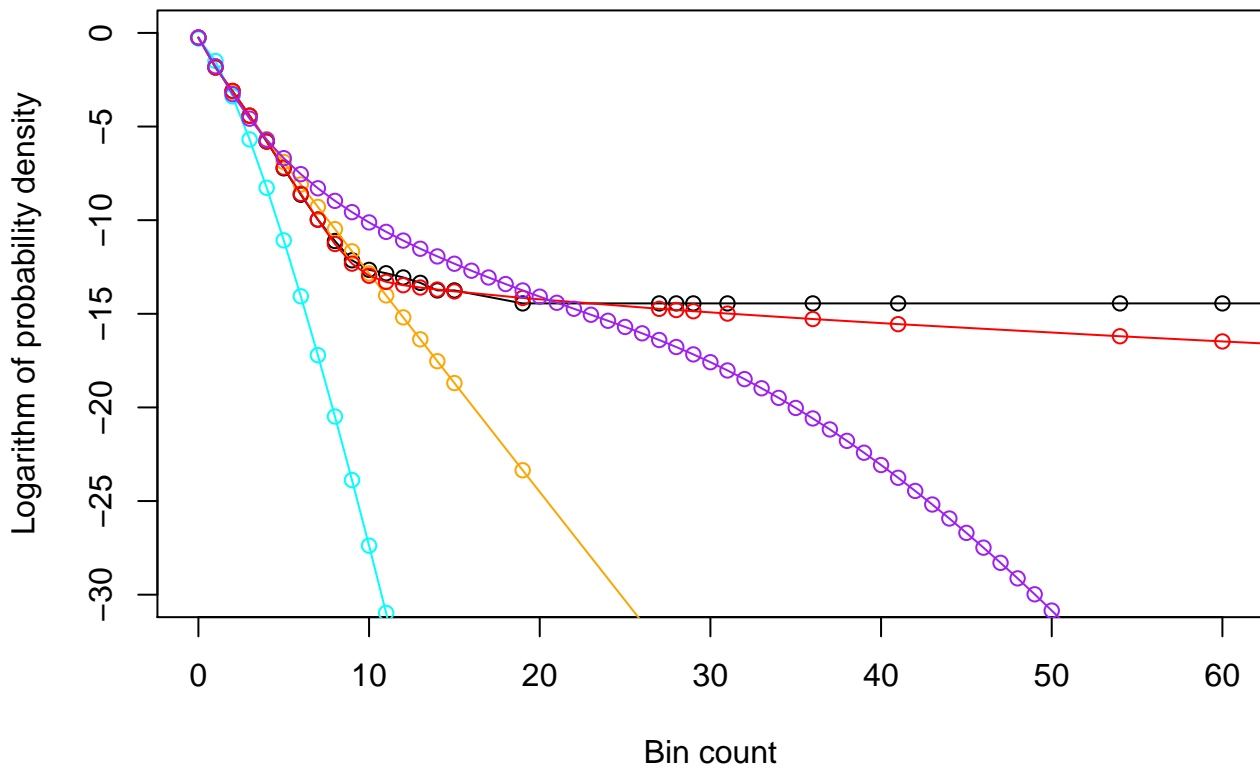

Supplement: Supplementary file 1 [file DataSheet1.ZIP › plots/s4Acount.pdf]

## Density Recovery – Sample C–ChIP

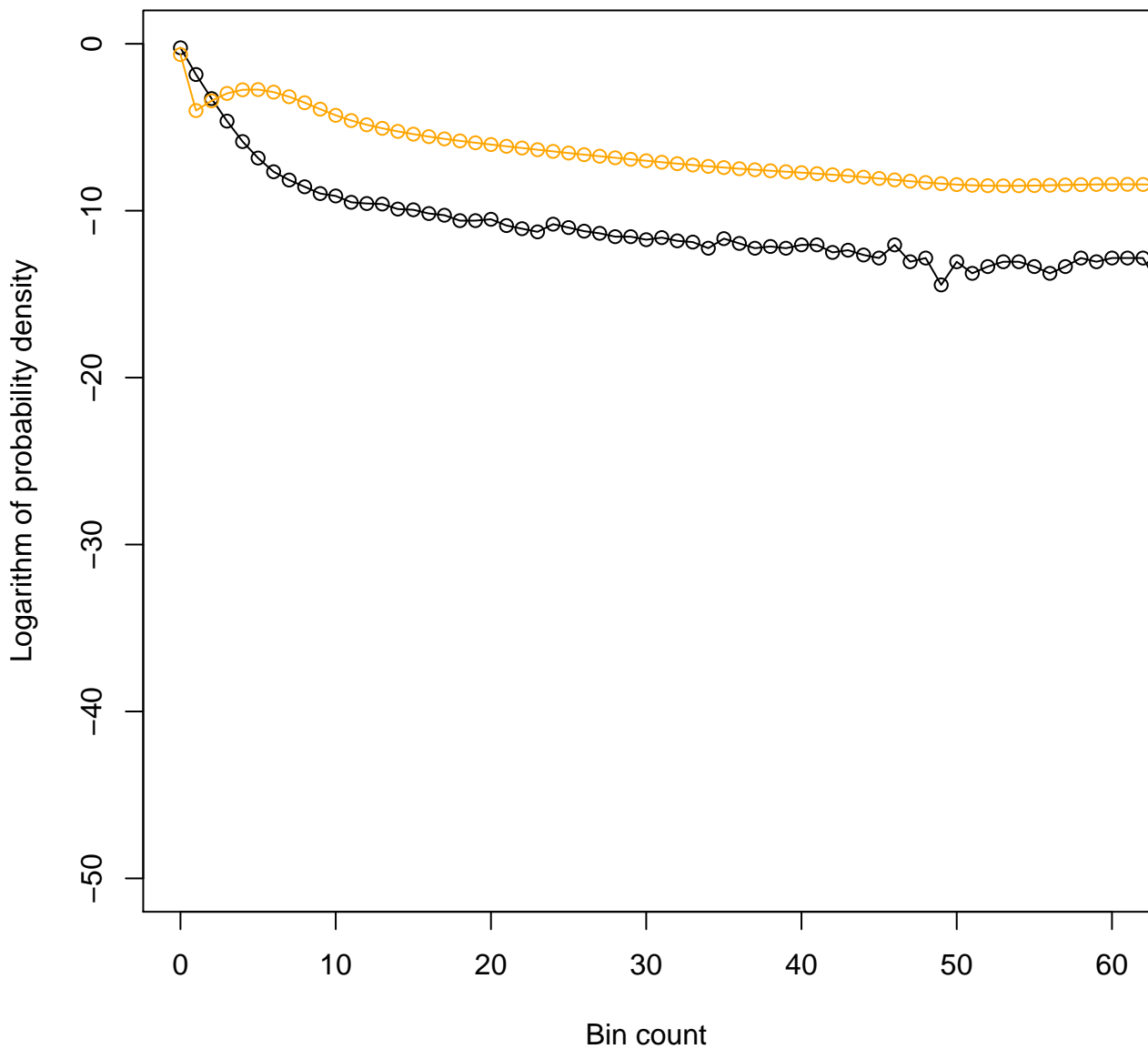

Supplement: Supplementary file 1 [file DataSheet1.ZIP › plots/s7B.pdf]

# Sample B-ChIP

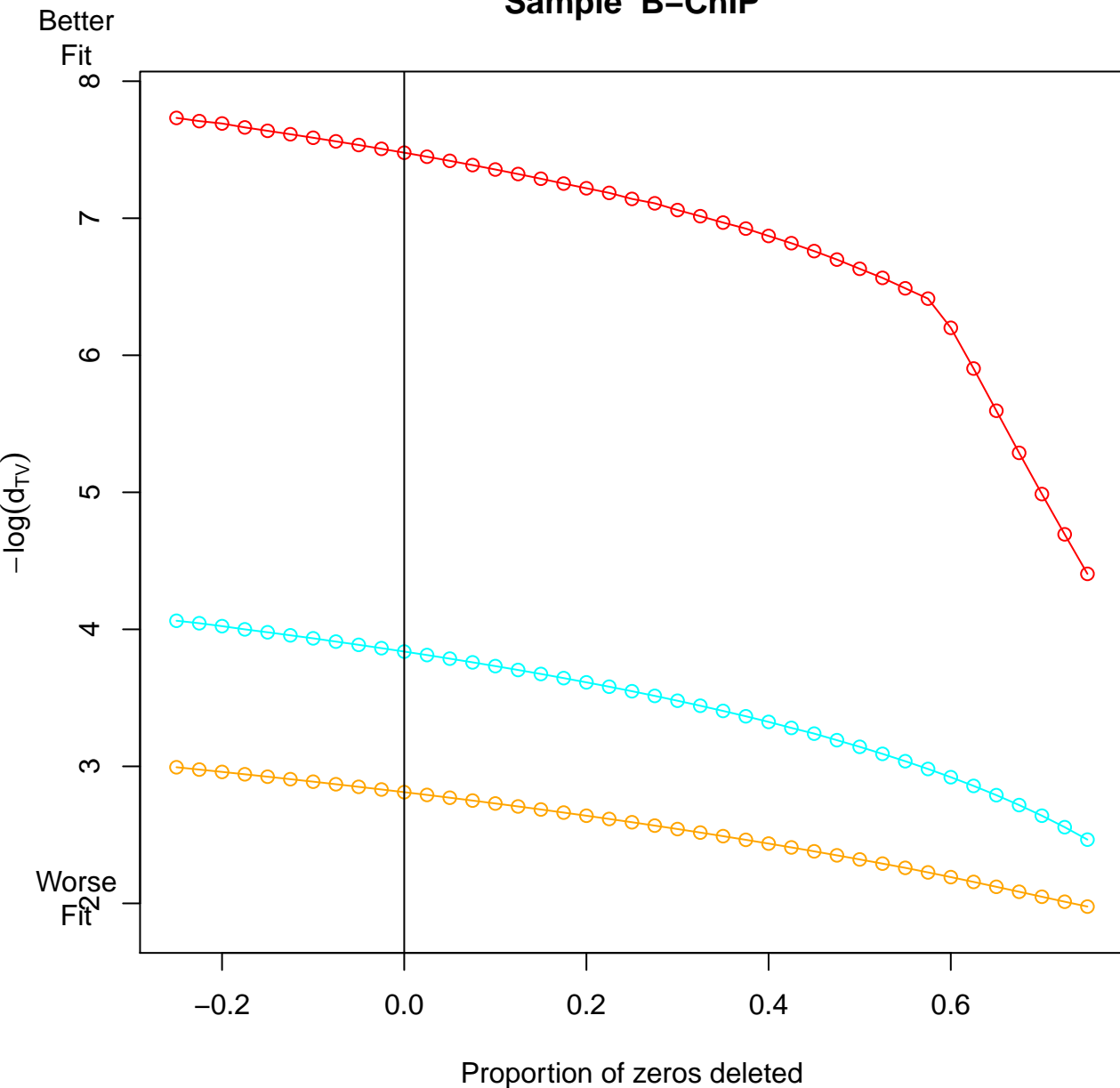

Supplement: Supplementary file 1 [file DataSheet1.ZIP › plots/s6TV.pdf]

**Sample D-NoDup**

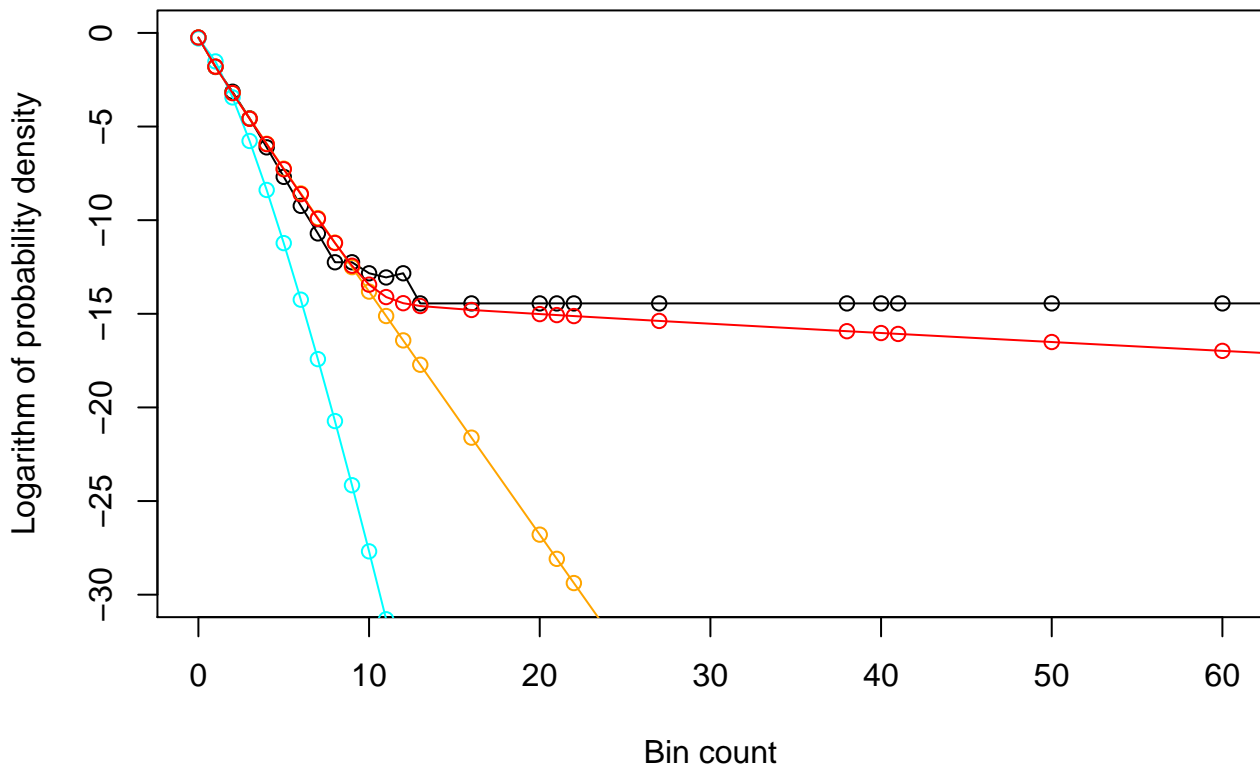

Supplement: Supplementary file 1 [file DataSheet1.ZIP › plots/s12Acount.pdf]

## Density Recovery – Sample B

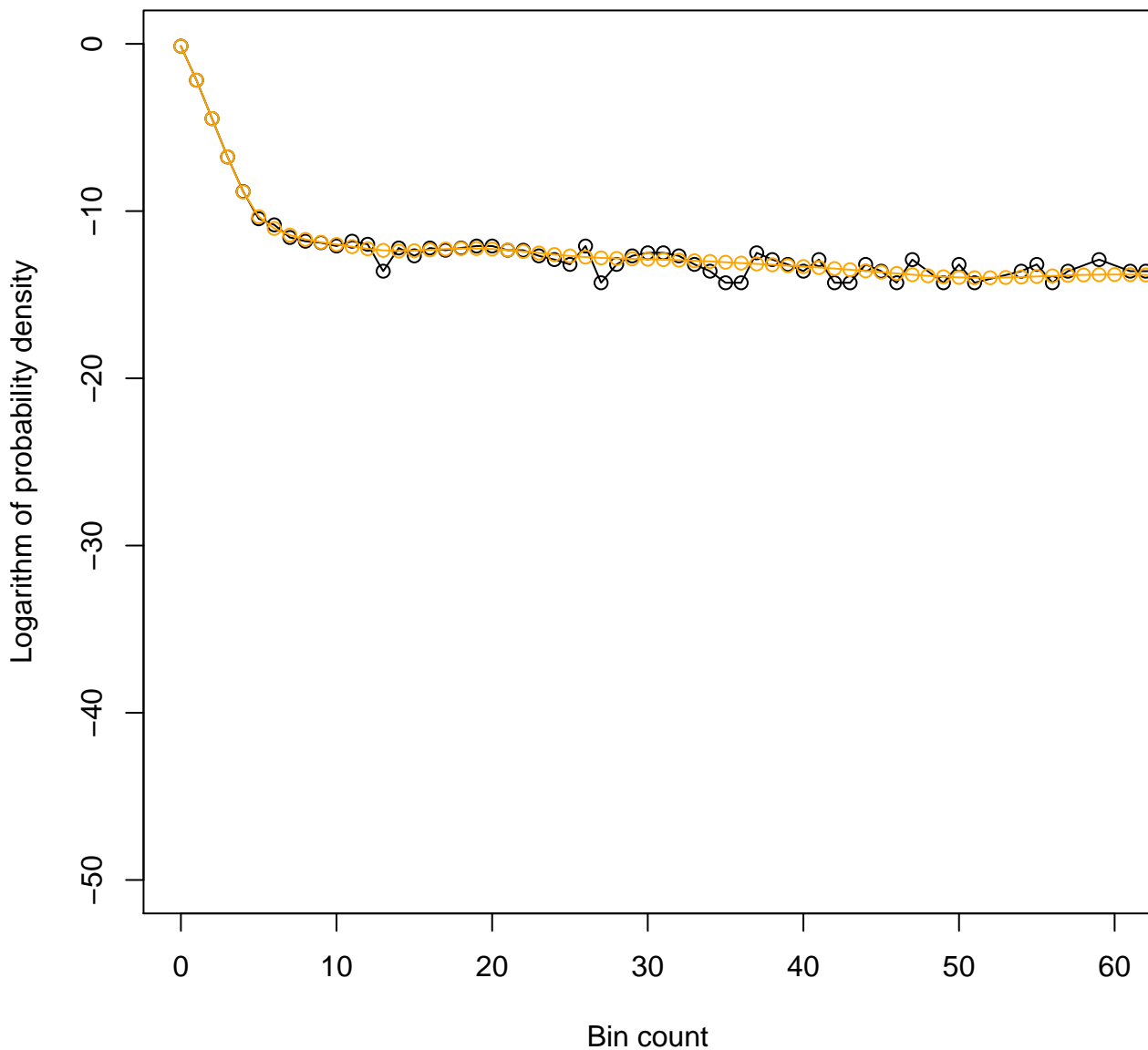

Supplement: Supplementary file 1 [file DataSheet1.ZIP › plots/s2B.pdf]

## Sample B-ChIP

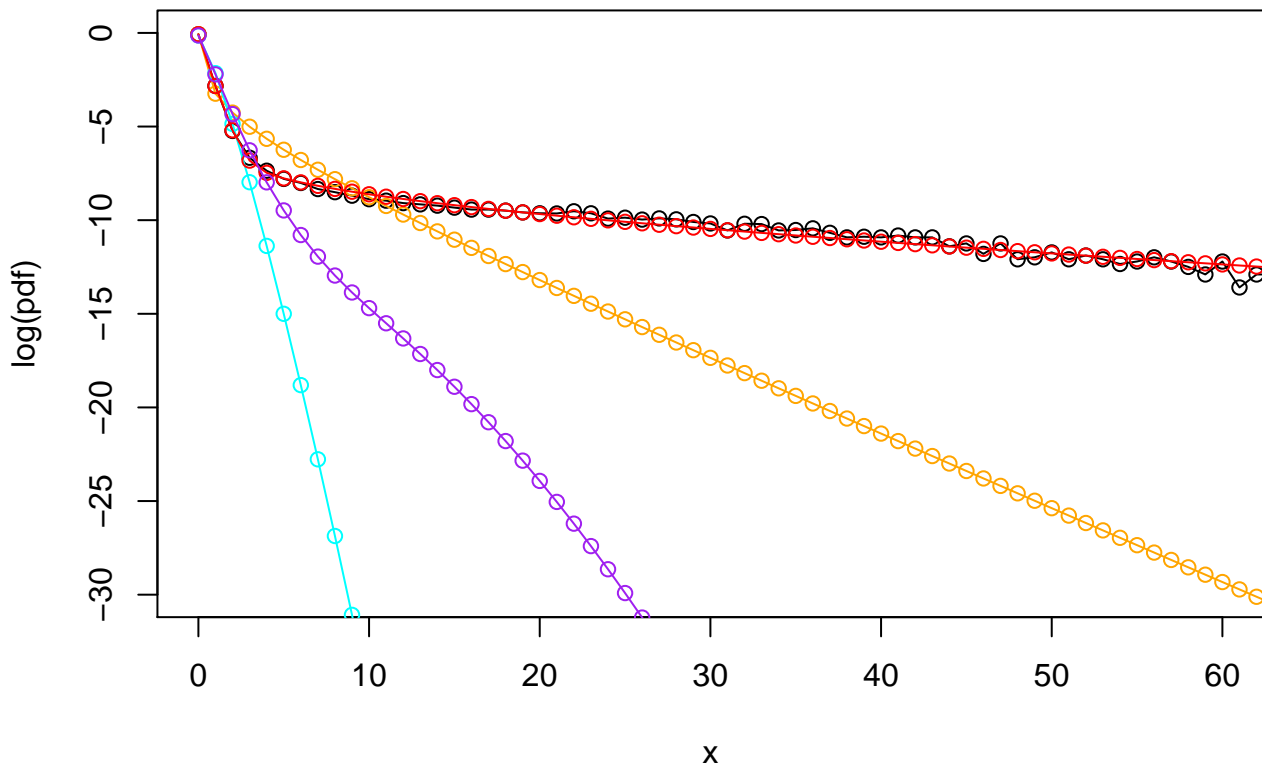

Supplement: Supplementary file 1 [file DataSheet1.ZIP › plots/s6Acount.pdf]

## Density Recovery – Sample A–NoDup

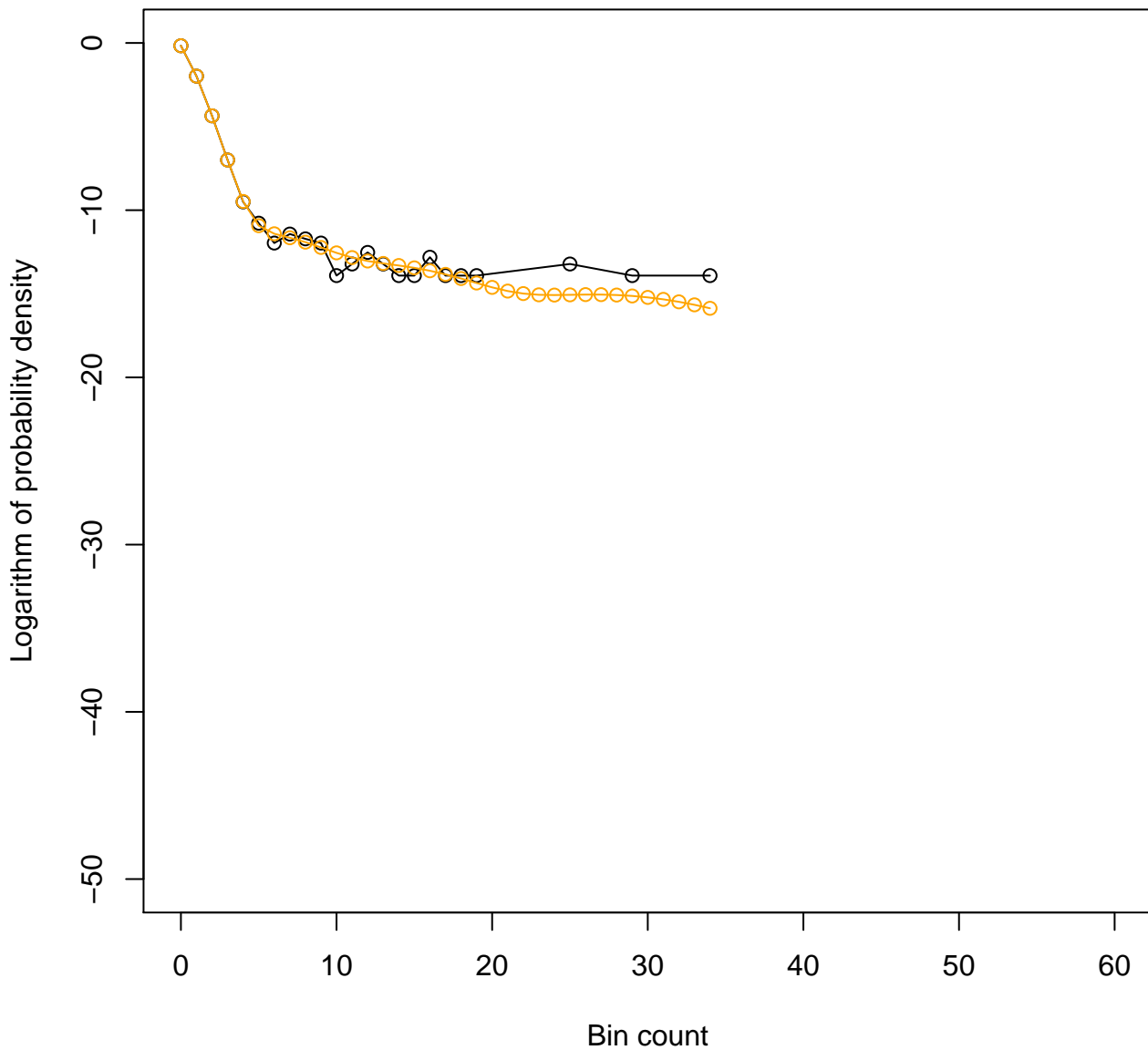

Supplement: Supplementary file 1 [file DataSheet1.ZIP › plots/s9B.pdf]

# Sample A-NoDup

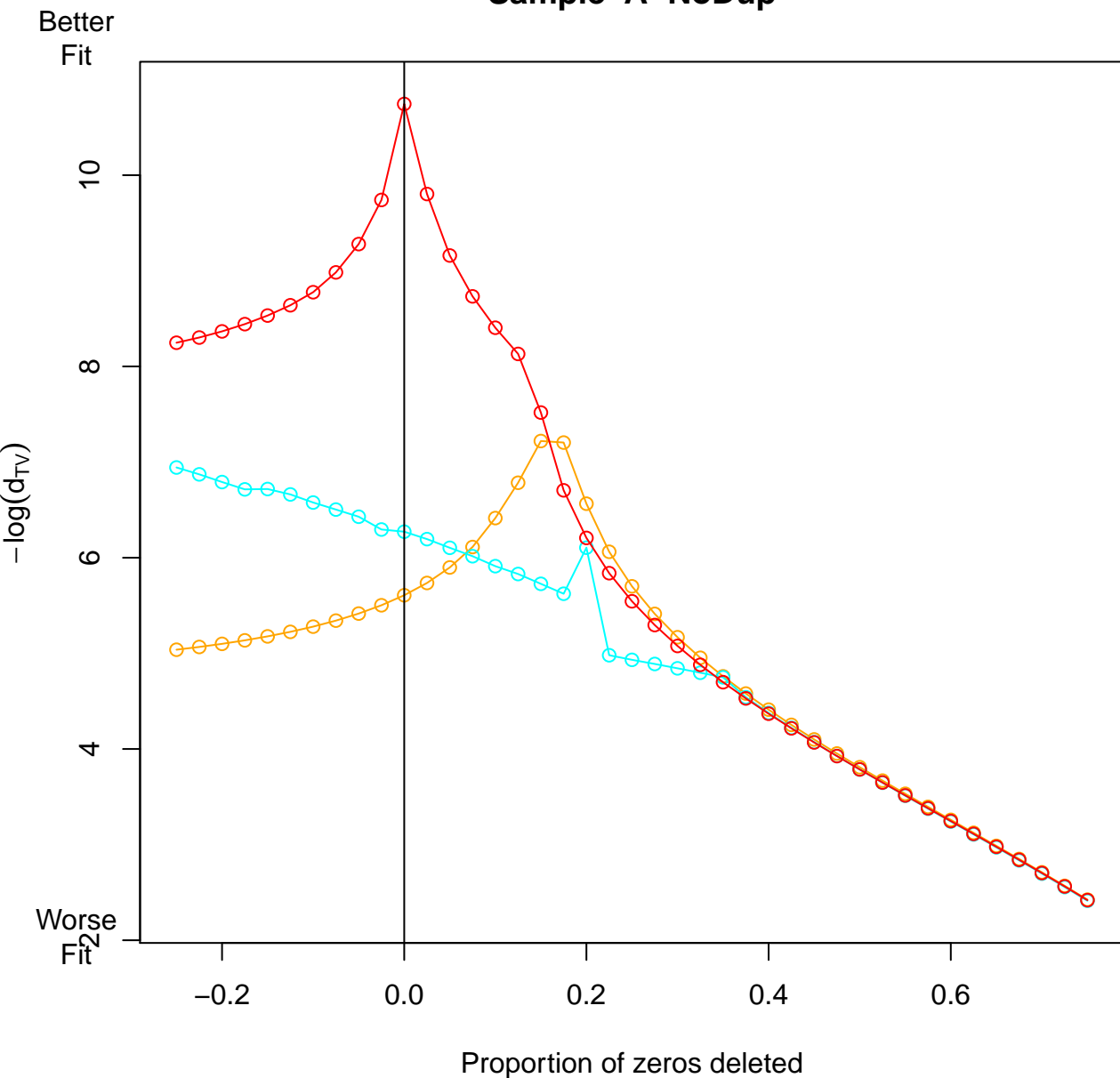

Supplement: Supplementary file 1 [file DataSheet1.ZIP › plots/s9TV.pdf]

## Sample C-NoDup

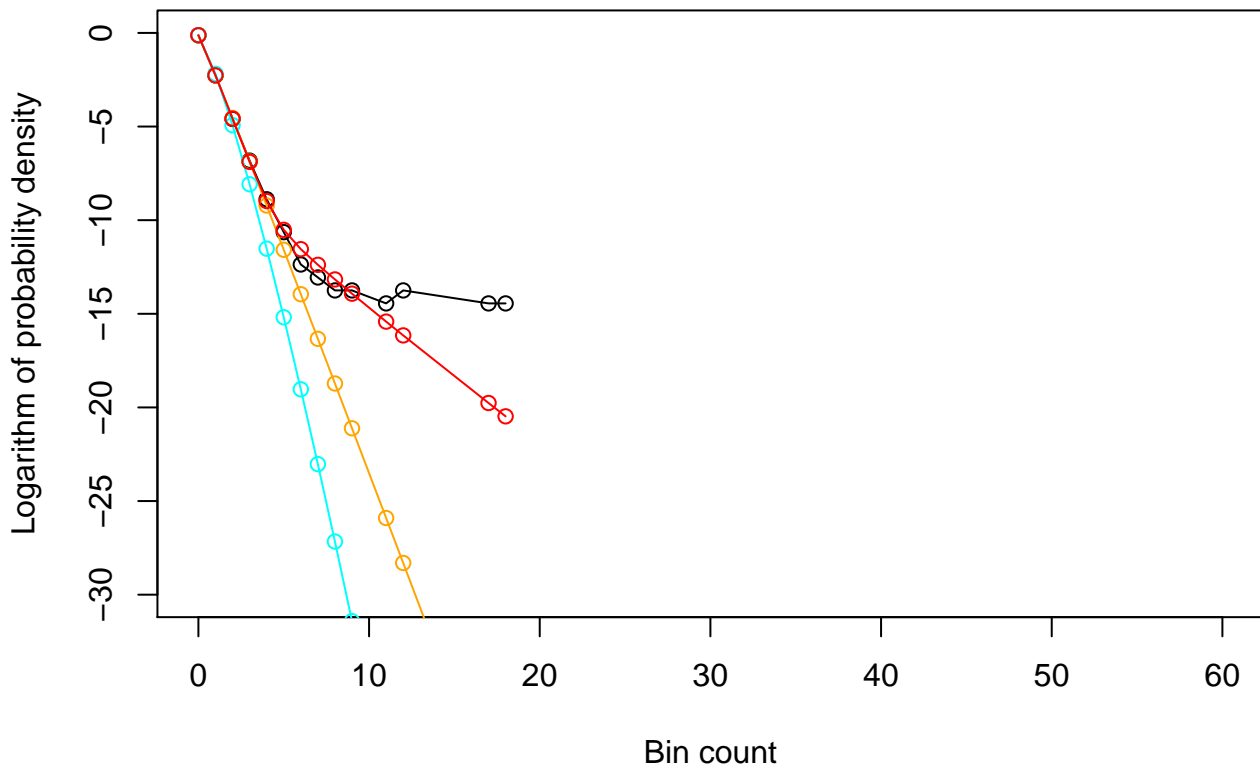

Supplement: Supplementary file 1 [file DataSheet1.ZIP › plots/s11Acount.pdf]

**Sample B**

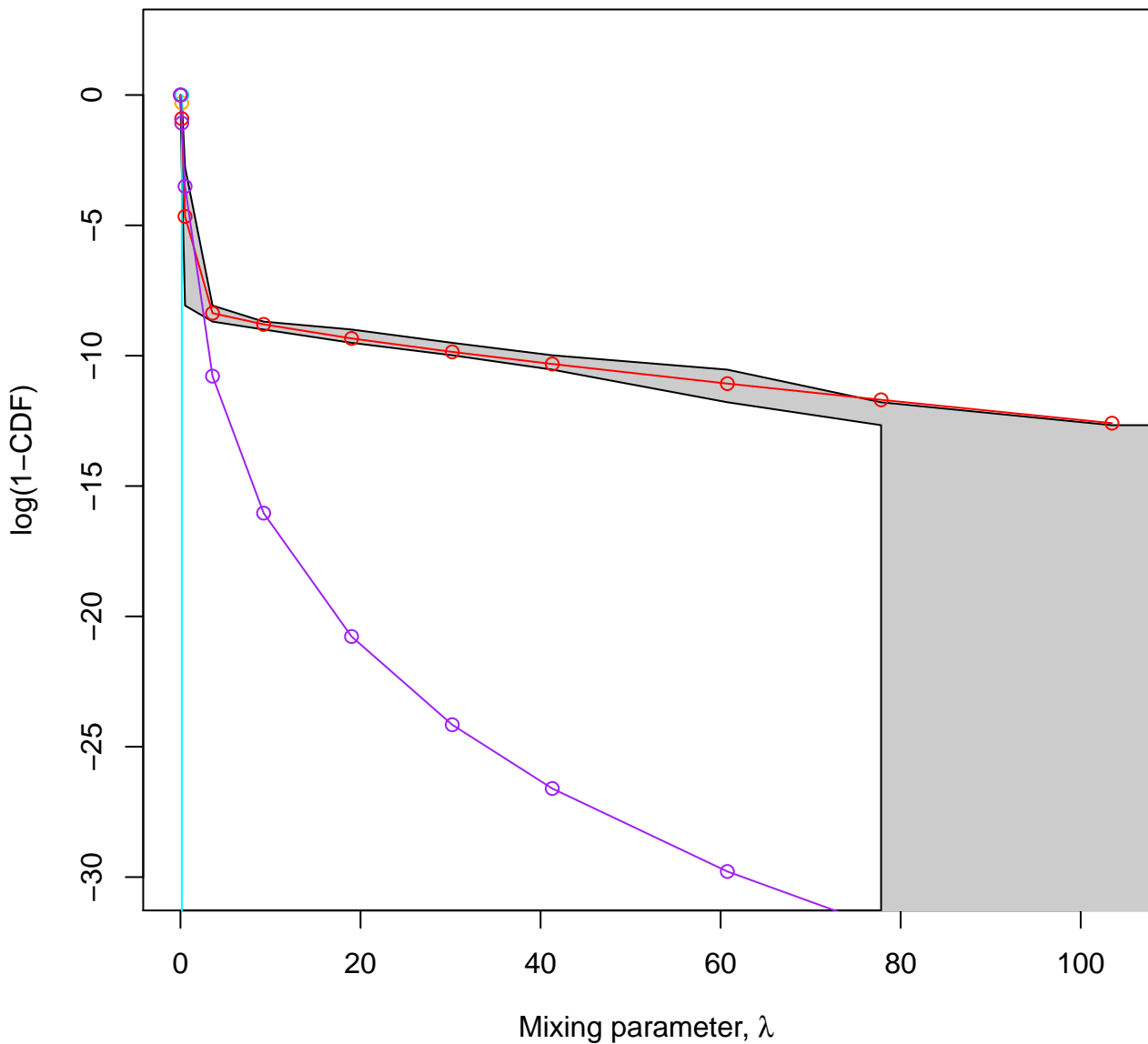

Supplement: Supplementary file 1 [file DataSheet1.ZIP › plots/s2Amix.pdf]

# Sample D-NoDup

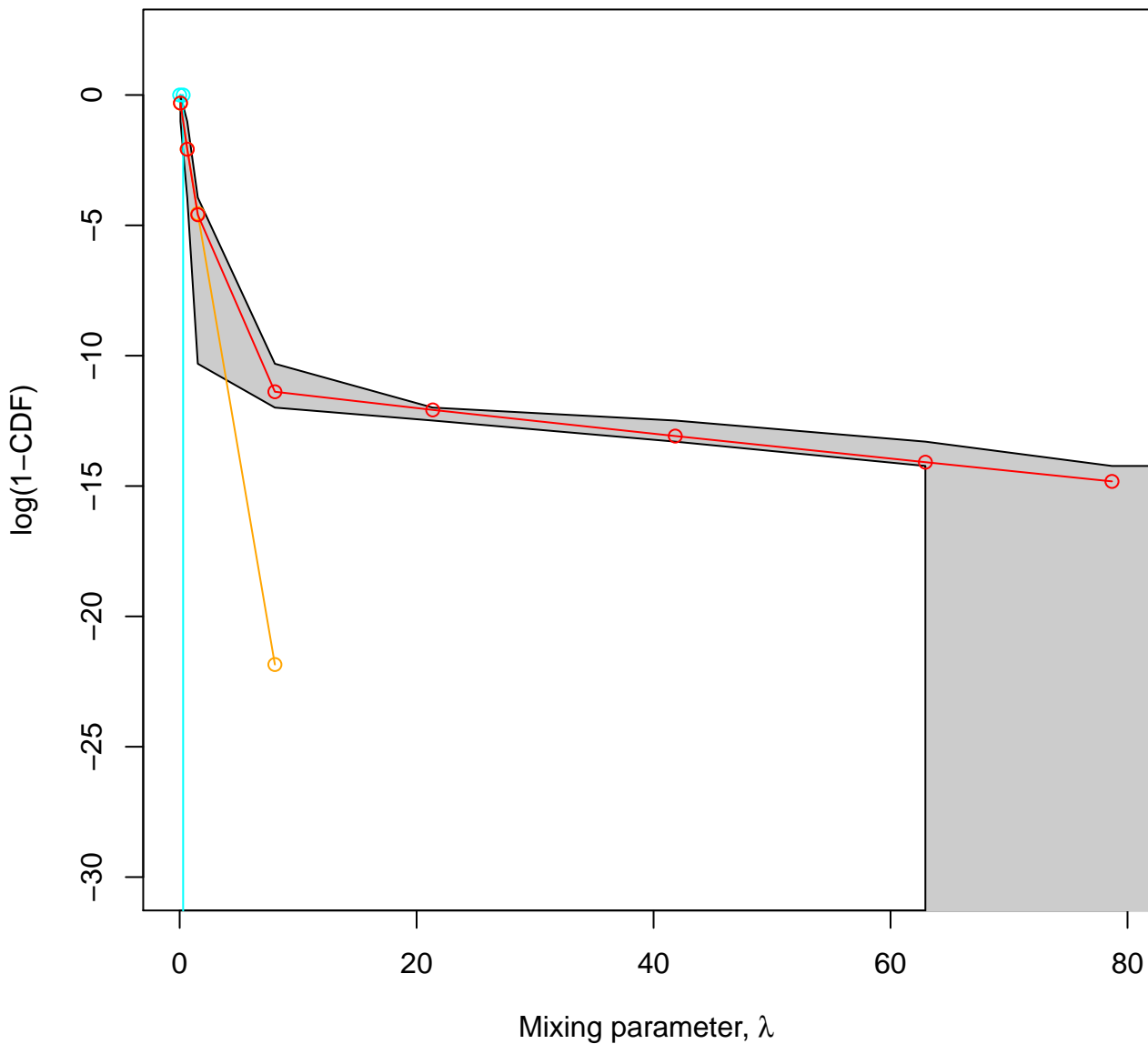

Supplement: Supplementary file 1 [file DataSheet1.ZIP › plots/s12Amix.pdf]

# Sample C

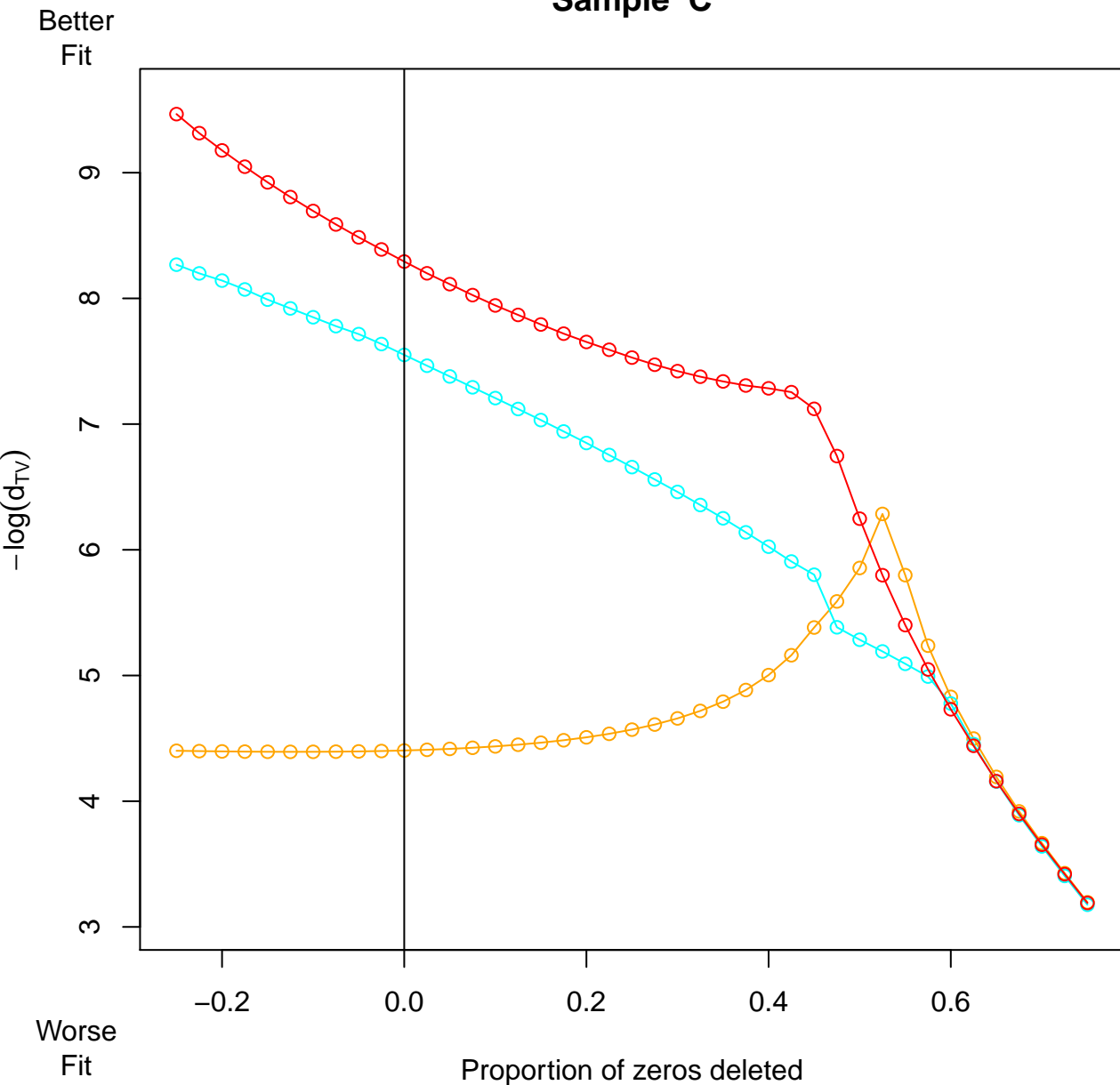

Supplement: Supplementary file 1 [file DataSheet1.ZIP › plots/s3TV.pdf]

## Density Recovery – Sample B–NoDup

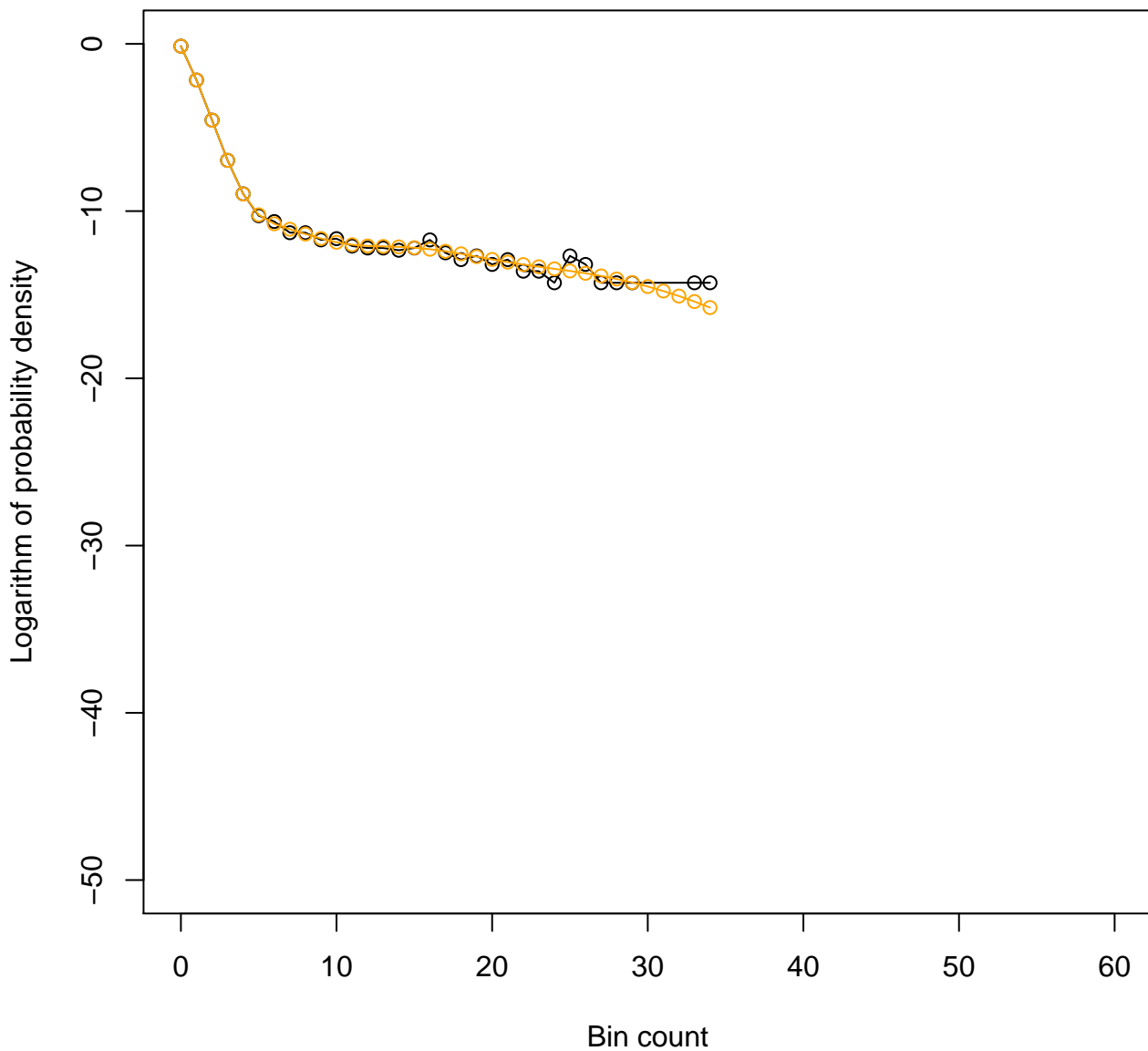

Supplement: Supplementary file 1 [file DataSheet1.ZIP › plots/s10B.pdf]

## Density Recovery – Sample D–NoDup

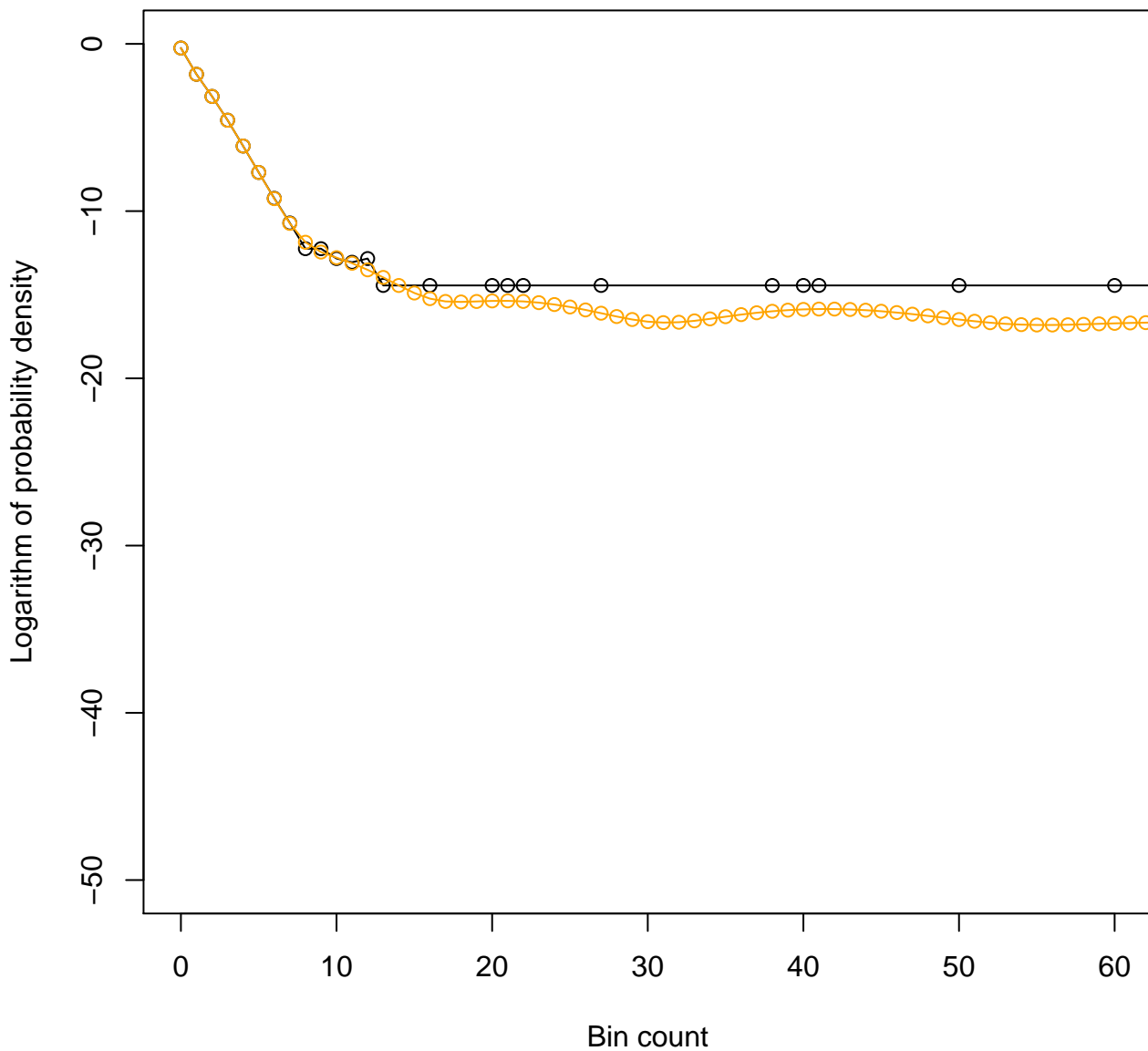

Supplement: Supplementary file 1 [file DataSheet1.ZIP › plots/s12B.pdf]

## Density Recovery – Sample C–NoDup

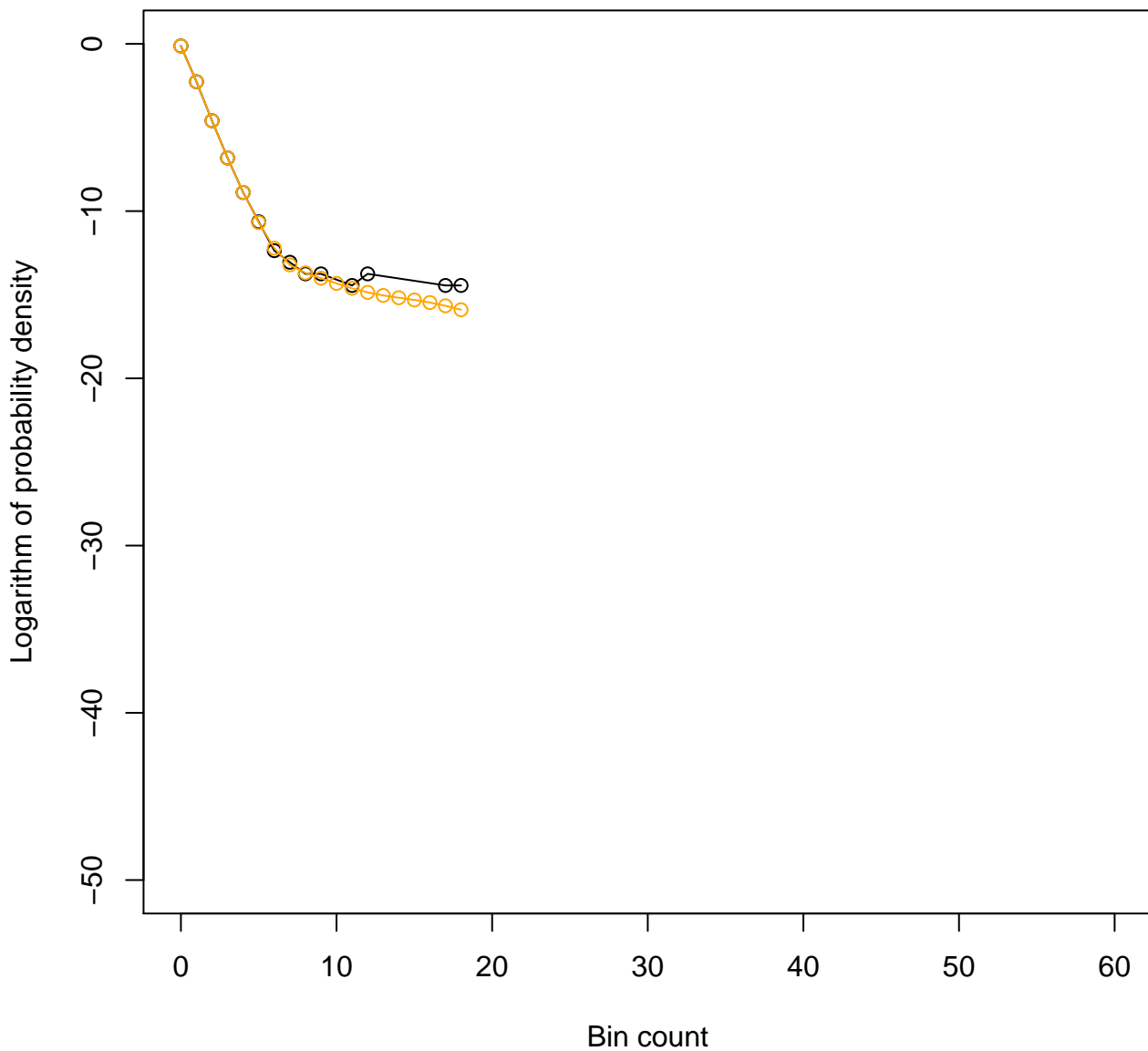

Supplement: Supplementary file 1 [file DataSheet1.ZIP › plots/s11B.pdf]

## Sample A-ChIP

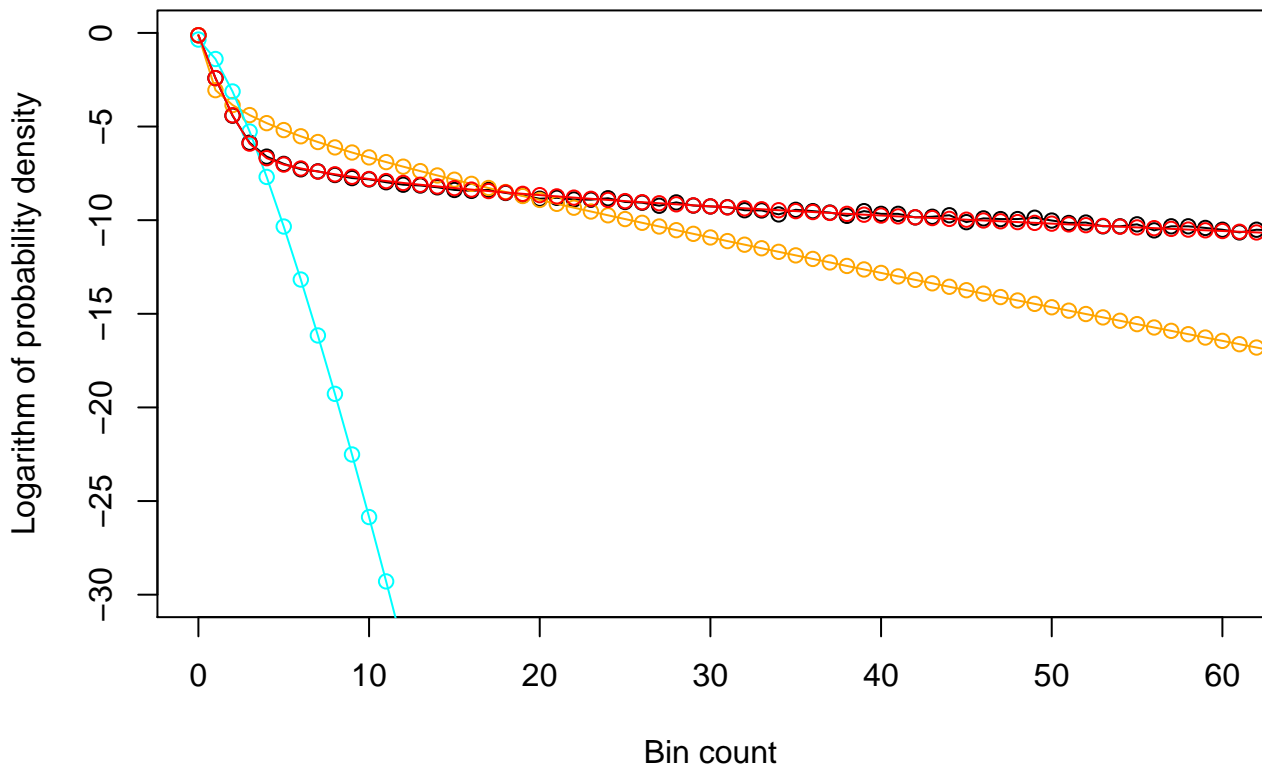

Supplement: Supplementary file 1 [file DataSheet1.ZIP › plots/s5Acount.pdf]

## Density Recovery – Sample D–ChIP

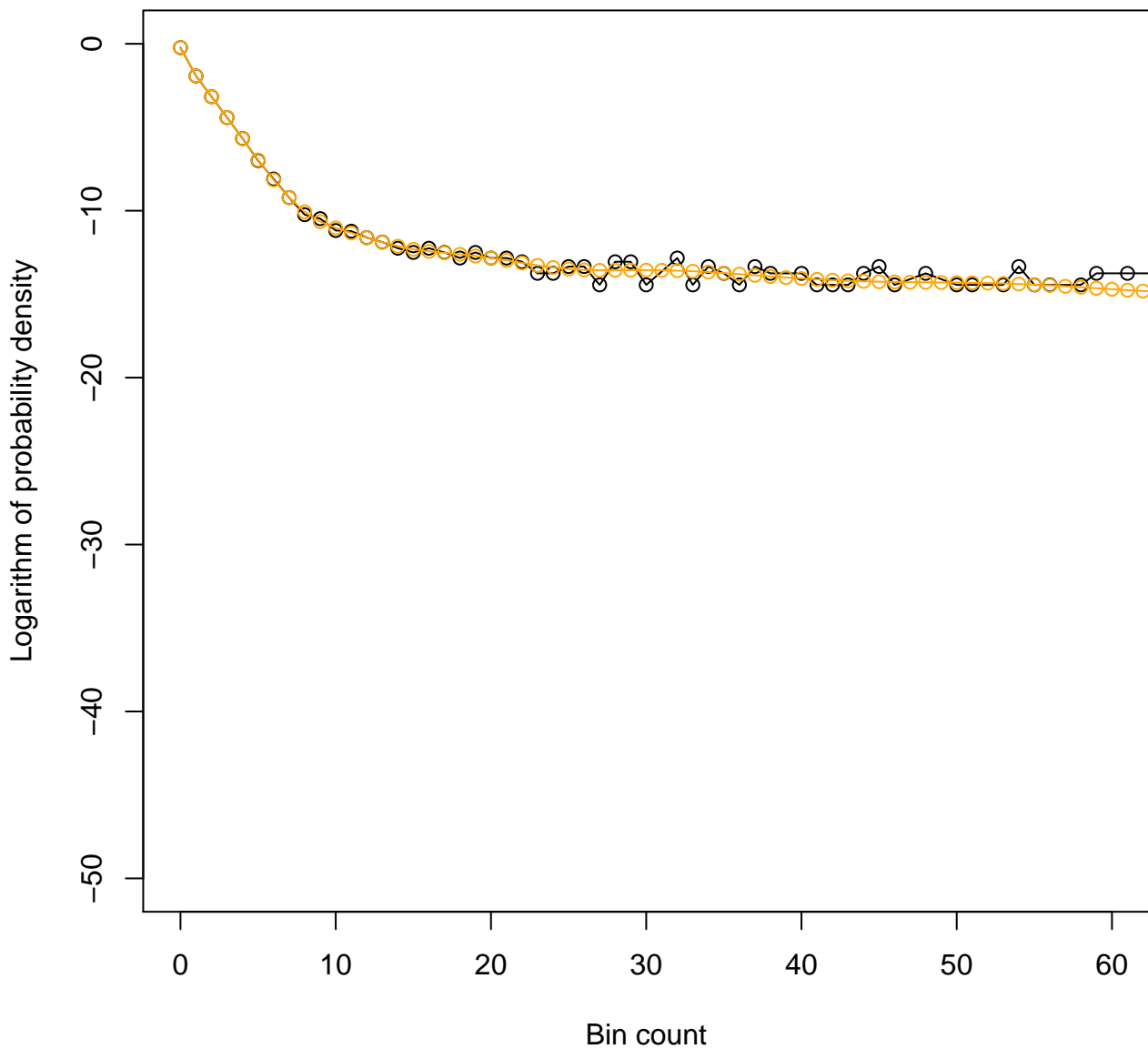

Supplement: Supplementary file 1 [file DataSheet1.ZIP › plots/s8B.pdf]

## Density Recovery – Sample A–ChIP

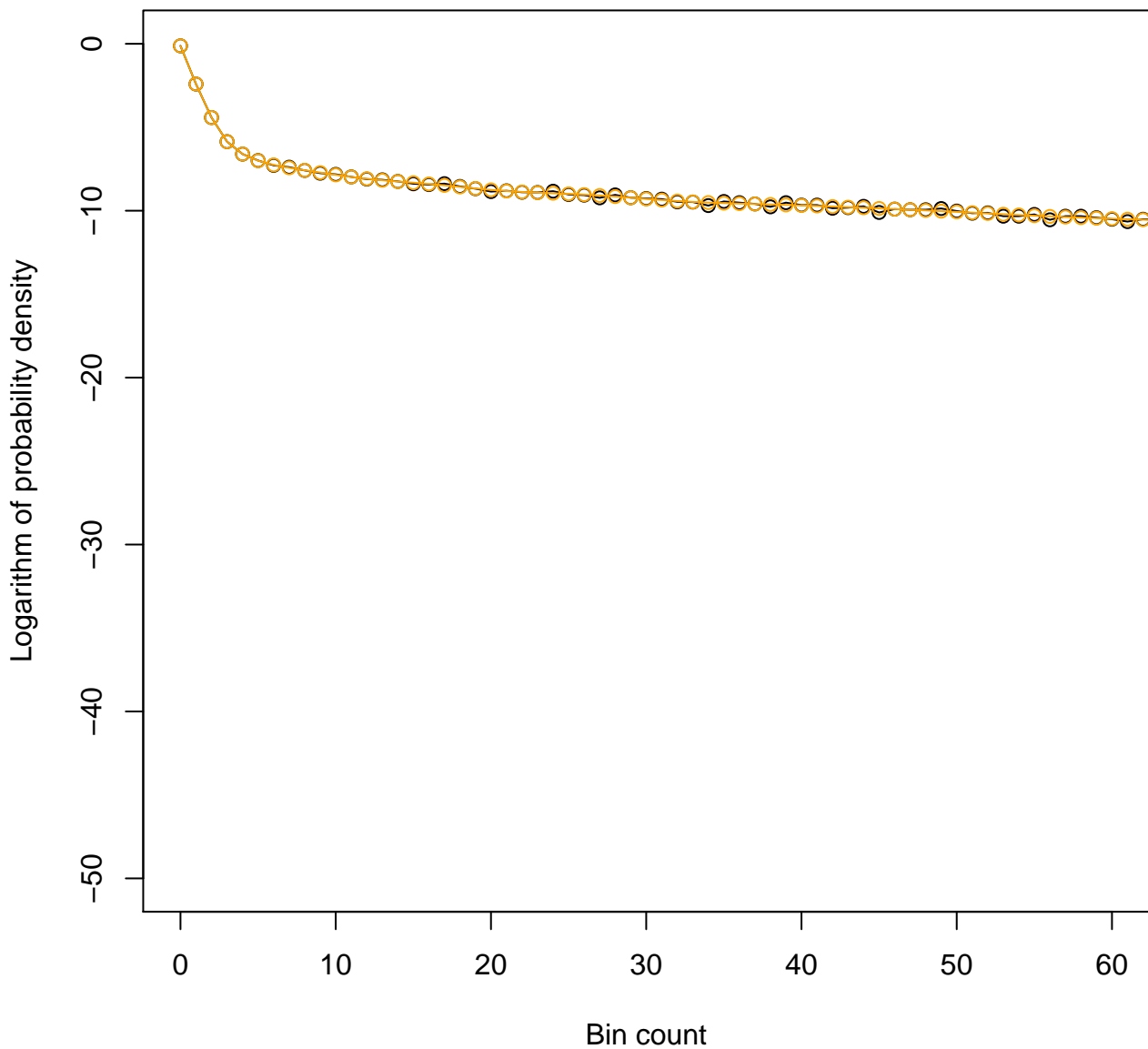

Supplement: Supplementary file 1 [file DataSheet1.ZIP › plots/s5B.pdf]

## Sample B-NoDup

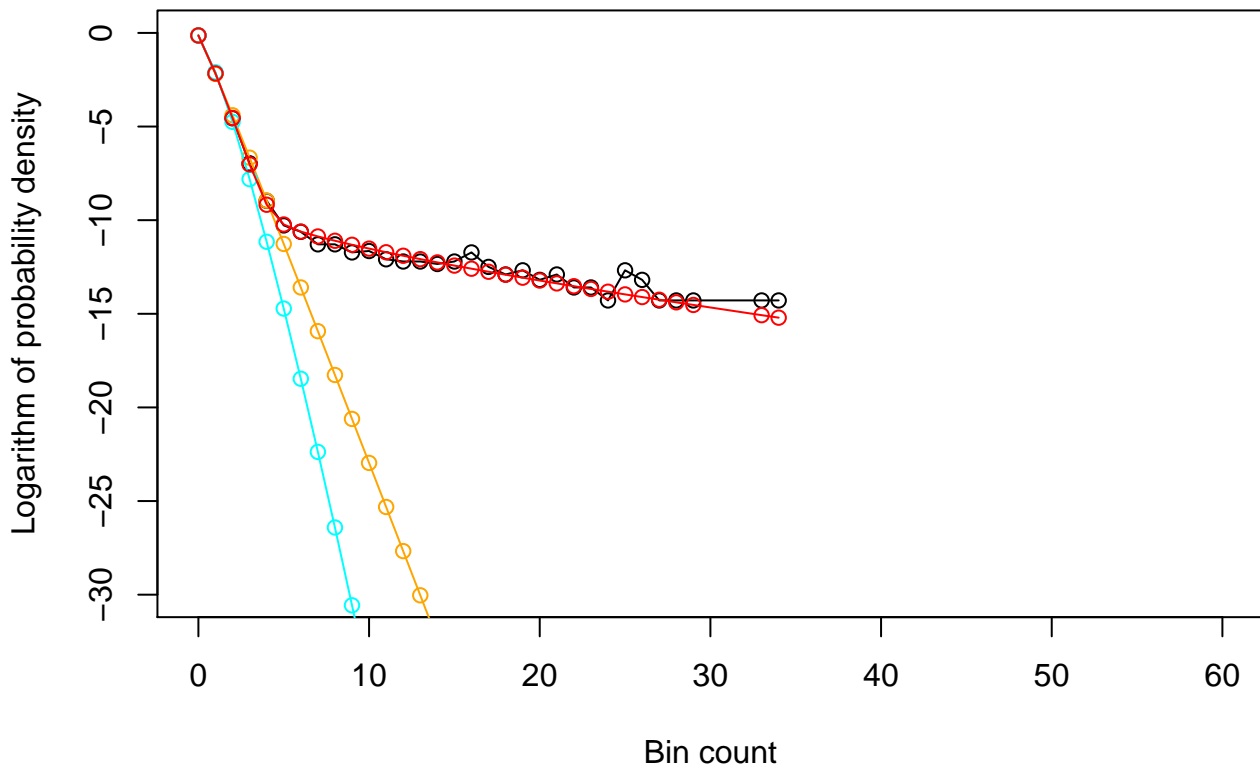

Supplement: Supplementary file 1 [file DataSheet1.ZIP › plots/s10Acount.pdf]

## Sample B

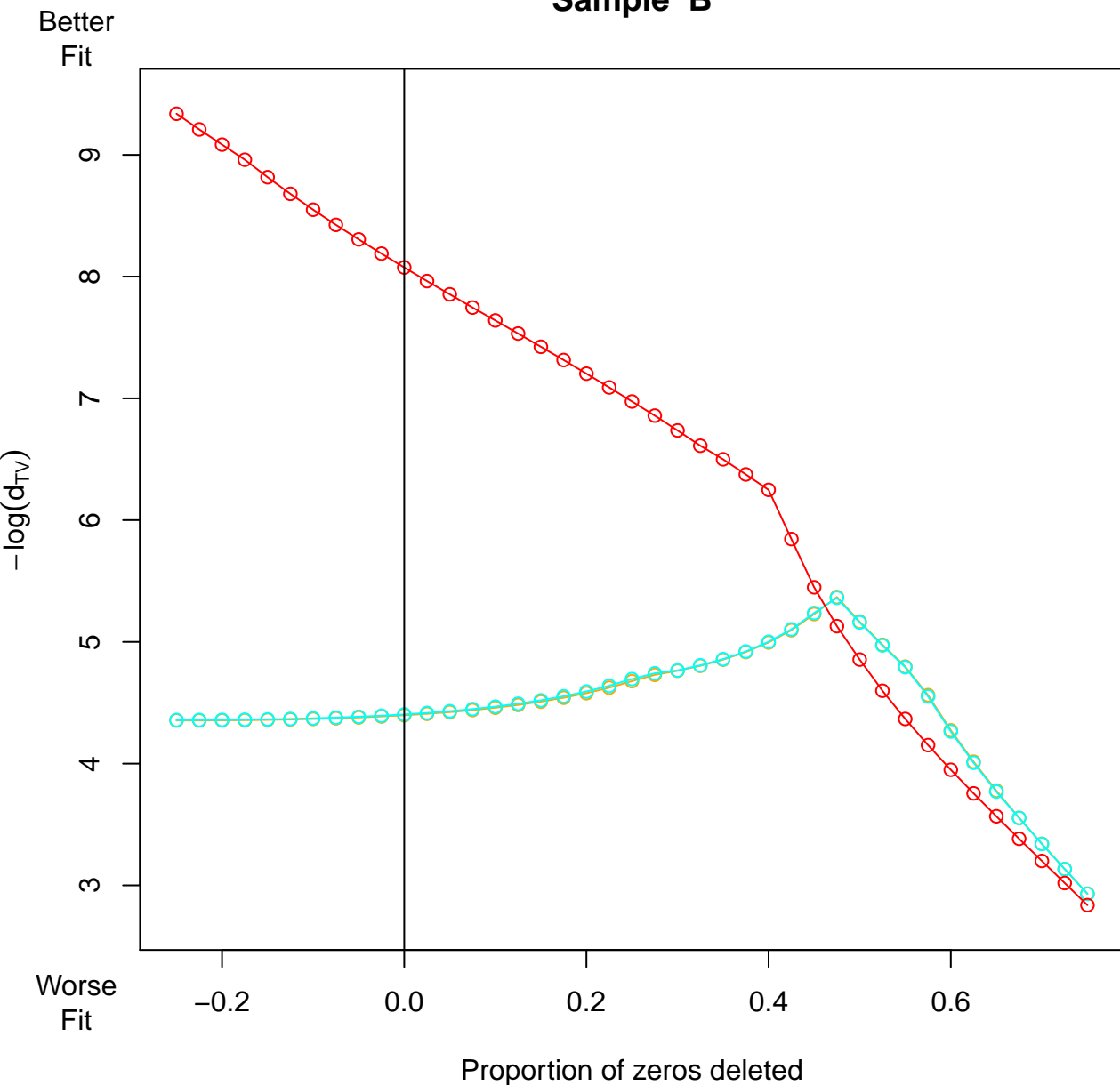

Supplement: Supplementary file 1 [file DataSheet1.ZIP › plots/s2TV.pdf]

## Density Recovery – Sample A

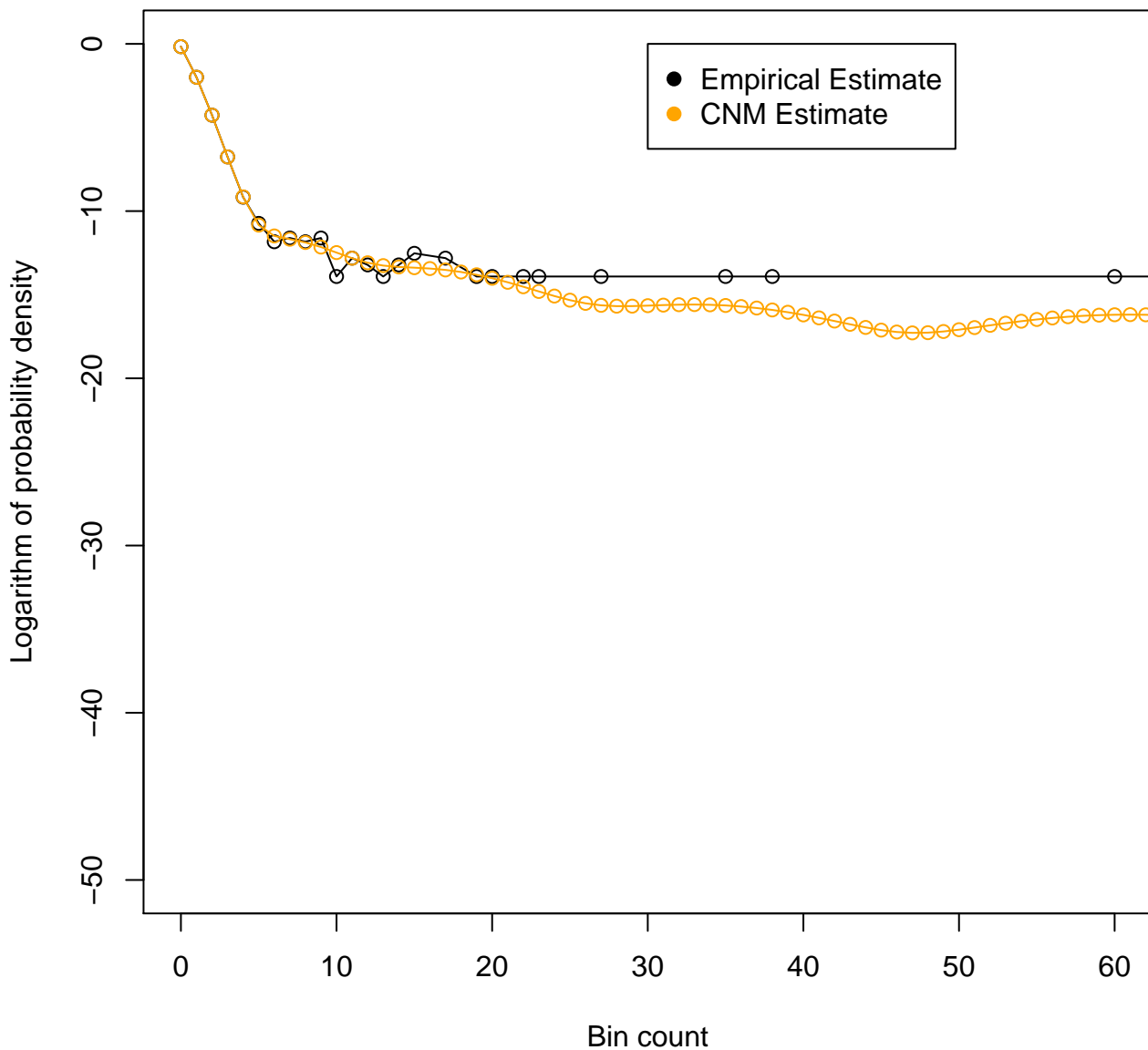

Supplement: Supplementary file 1 [file DataSheet1.ZIP › plots/s1B.pdf]

# Sample C-ChIP

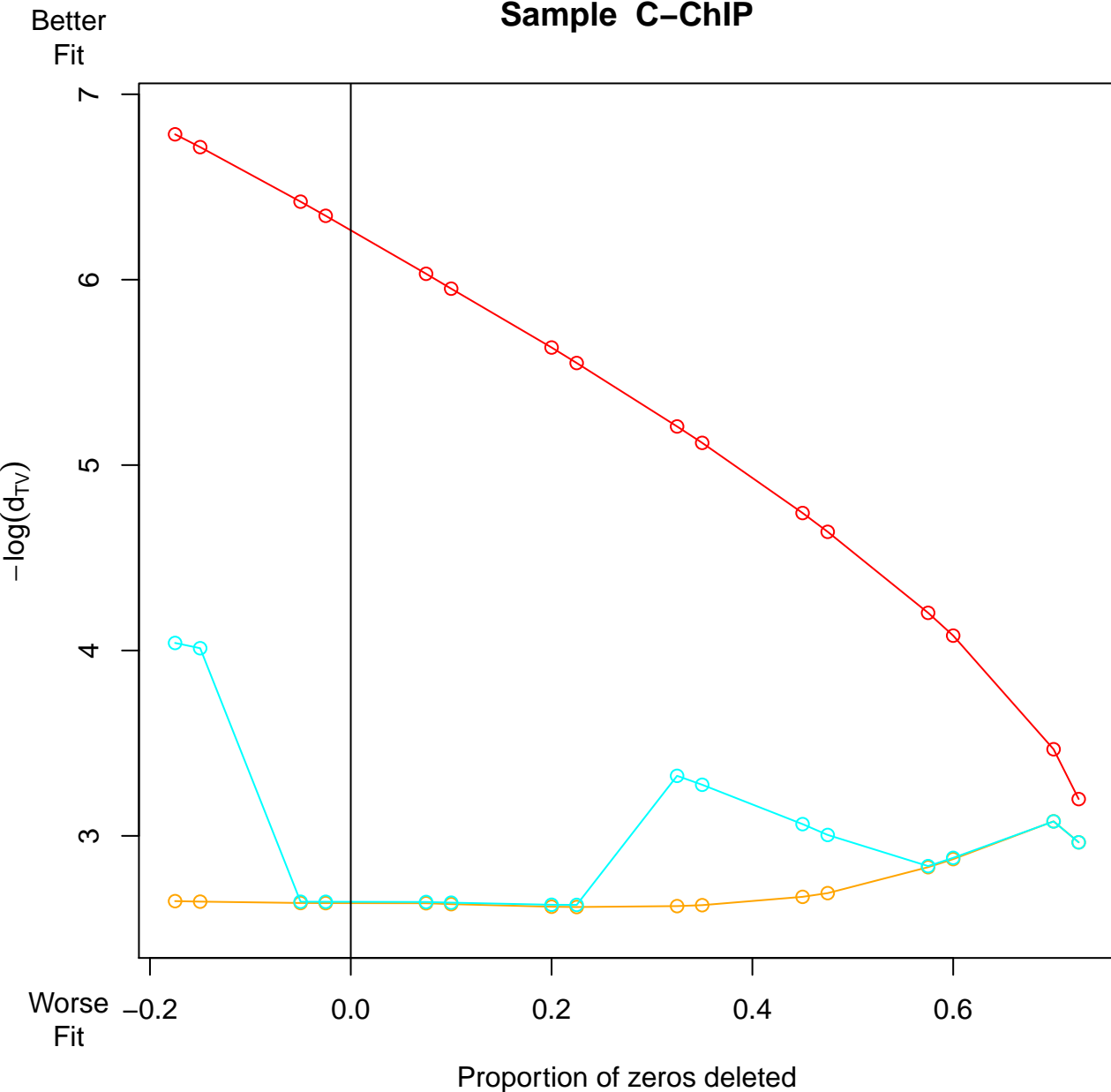

Supplement: Supplementary file 1 [file DataSheet1.ZIP › plots/s7TV.pdf]

# Sample C-ChIP

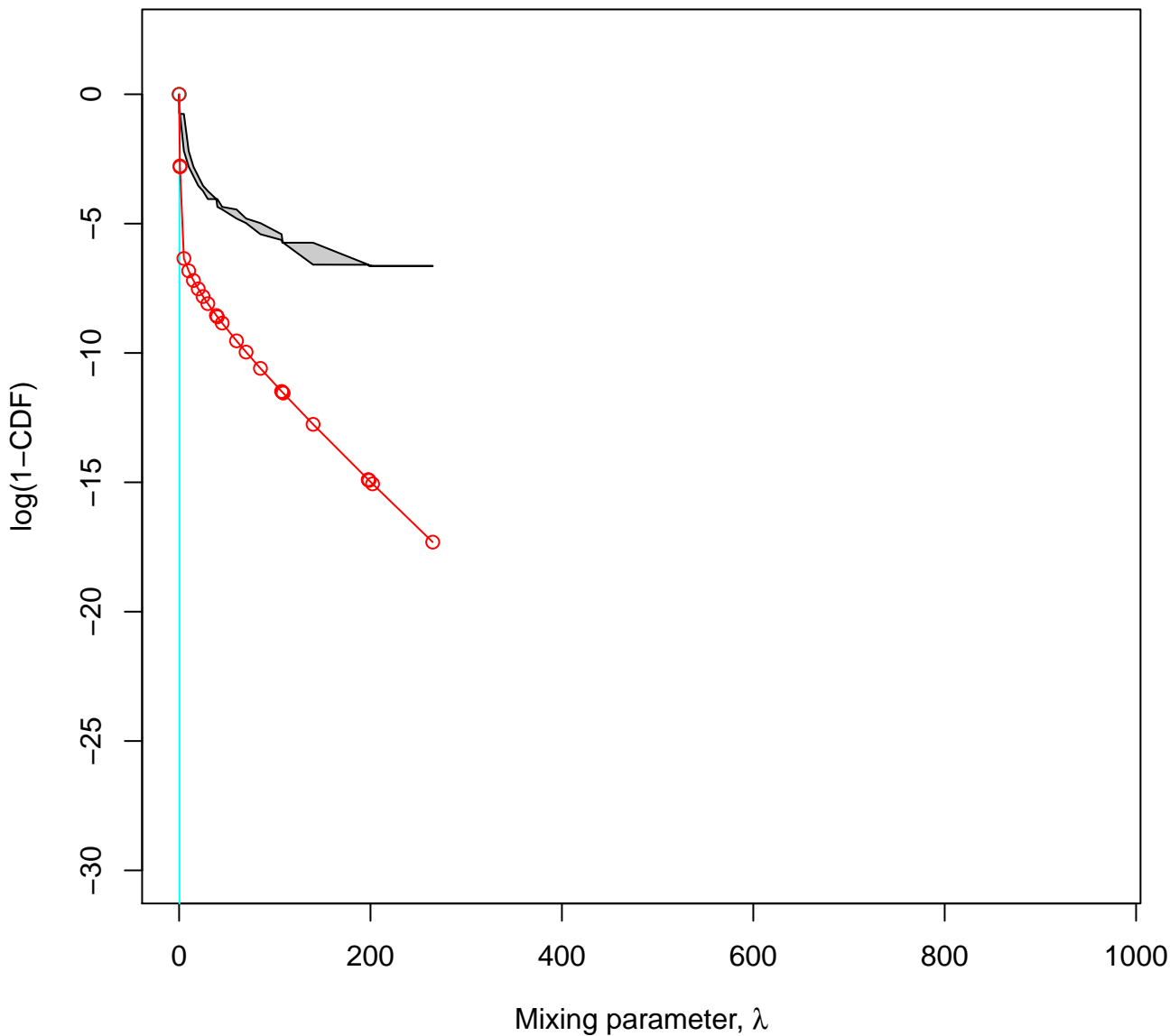

Supplement: Supplementary file 1 [file DataSheet1.ZIP › plots/s7Amix.pdf]

Sample D

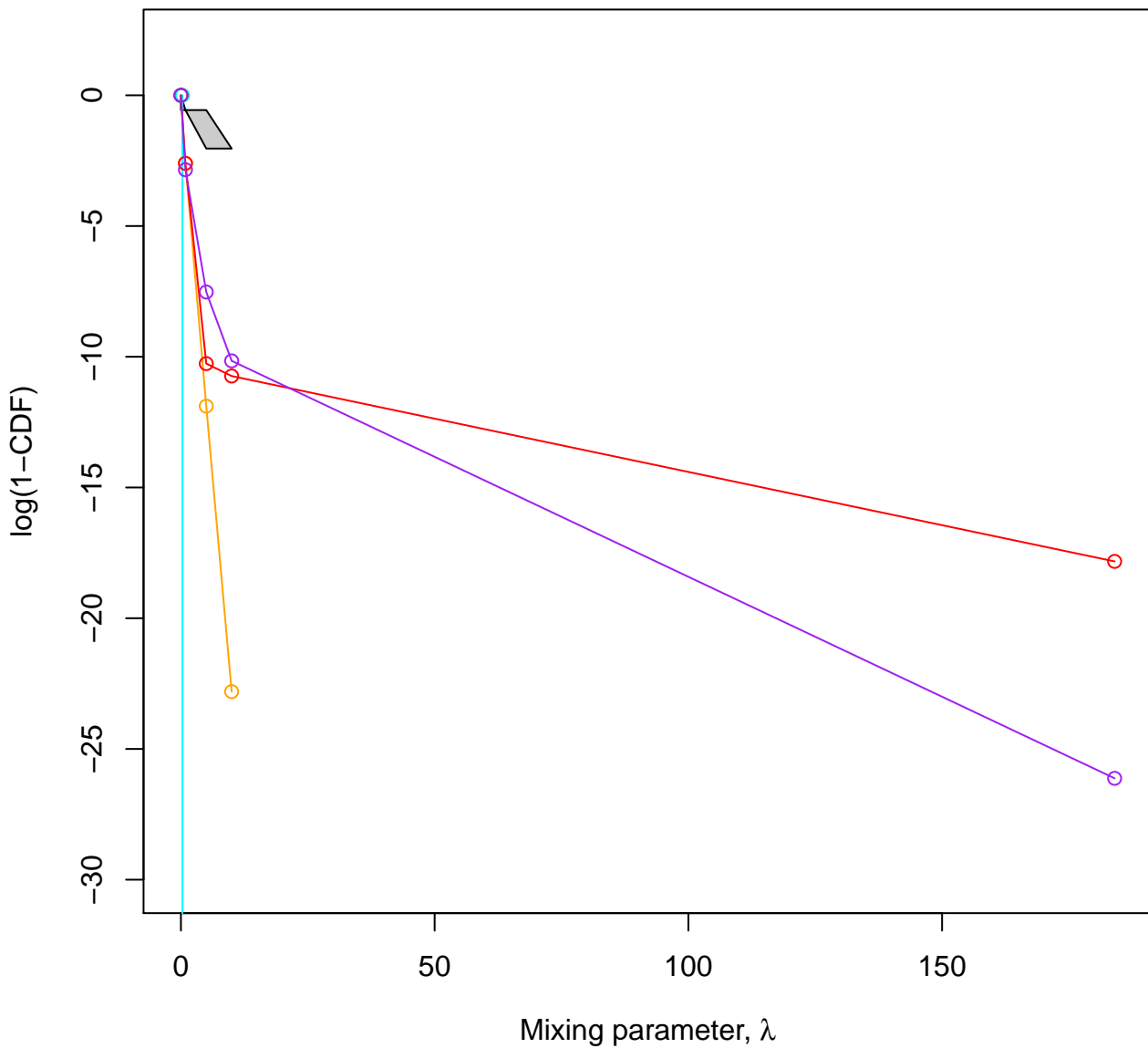

Supplement: Supplementary file 1 [file DataSheet1.ZIP › plots/s4Amix.pdf]

# Sample C-NoDup

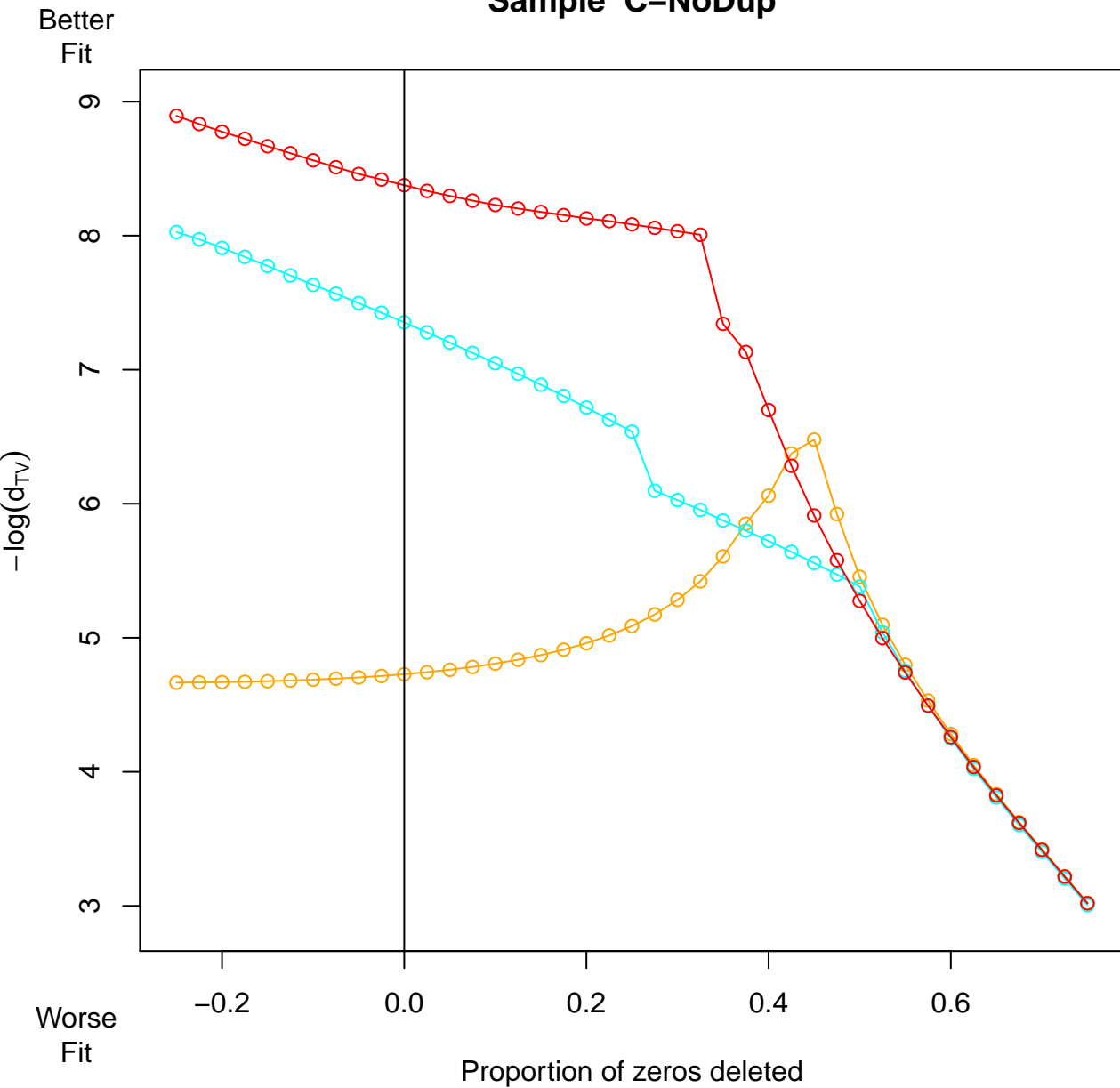

Supplement: Supplementary file 1 [file DataSheet1.ZIP › plots/s11TV.pdf]

# Sample B-NoDup

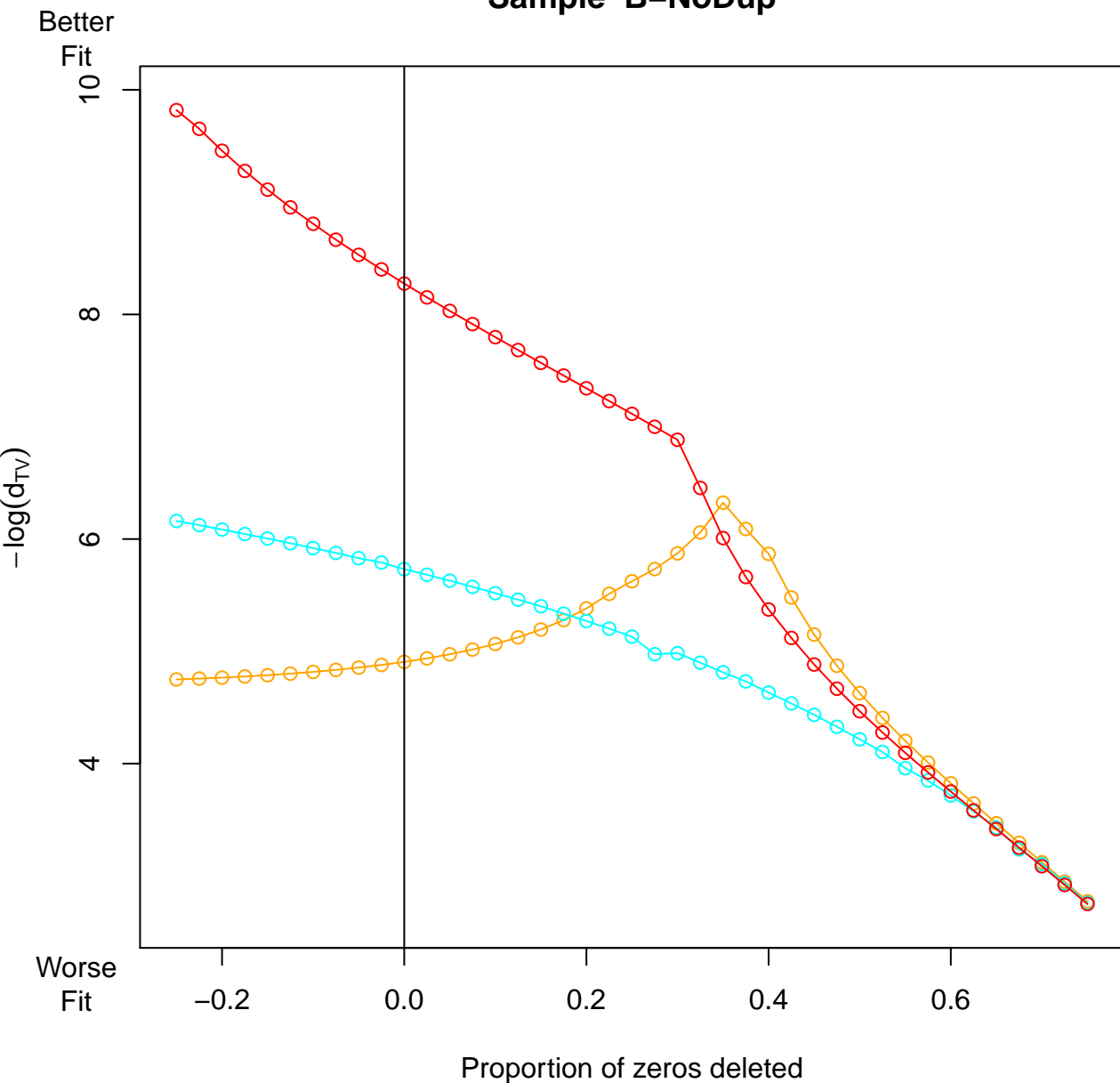

Supplement: Supplementary file 1 [file DataSheet1.ZIP › plots/s10TV.pdf]

**Total variation for each distribution**

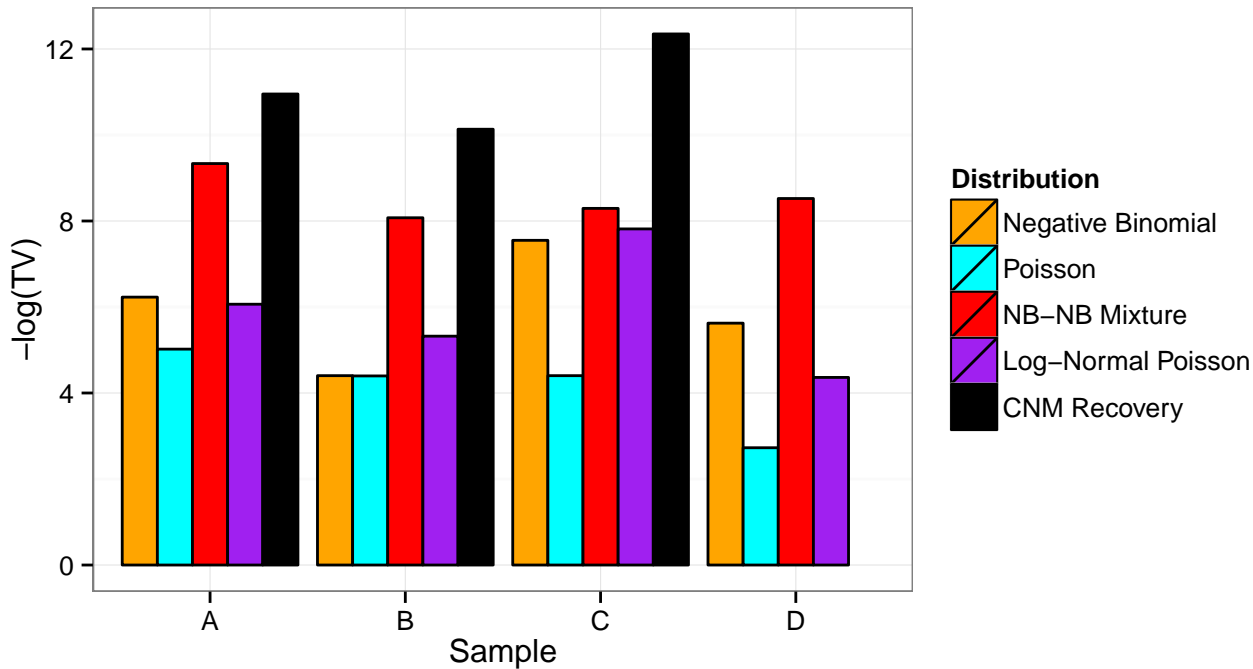

Supplement: Supplementary file 1 [file DataSheet1.ZIP › plots/TV.pdf]

# Sample A

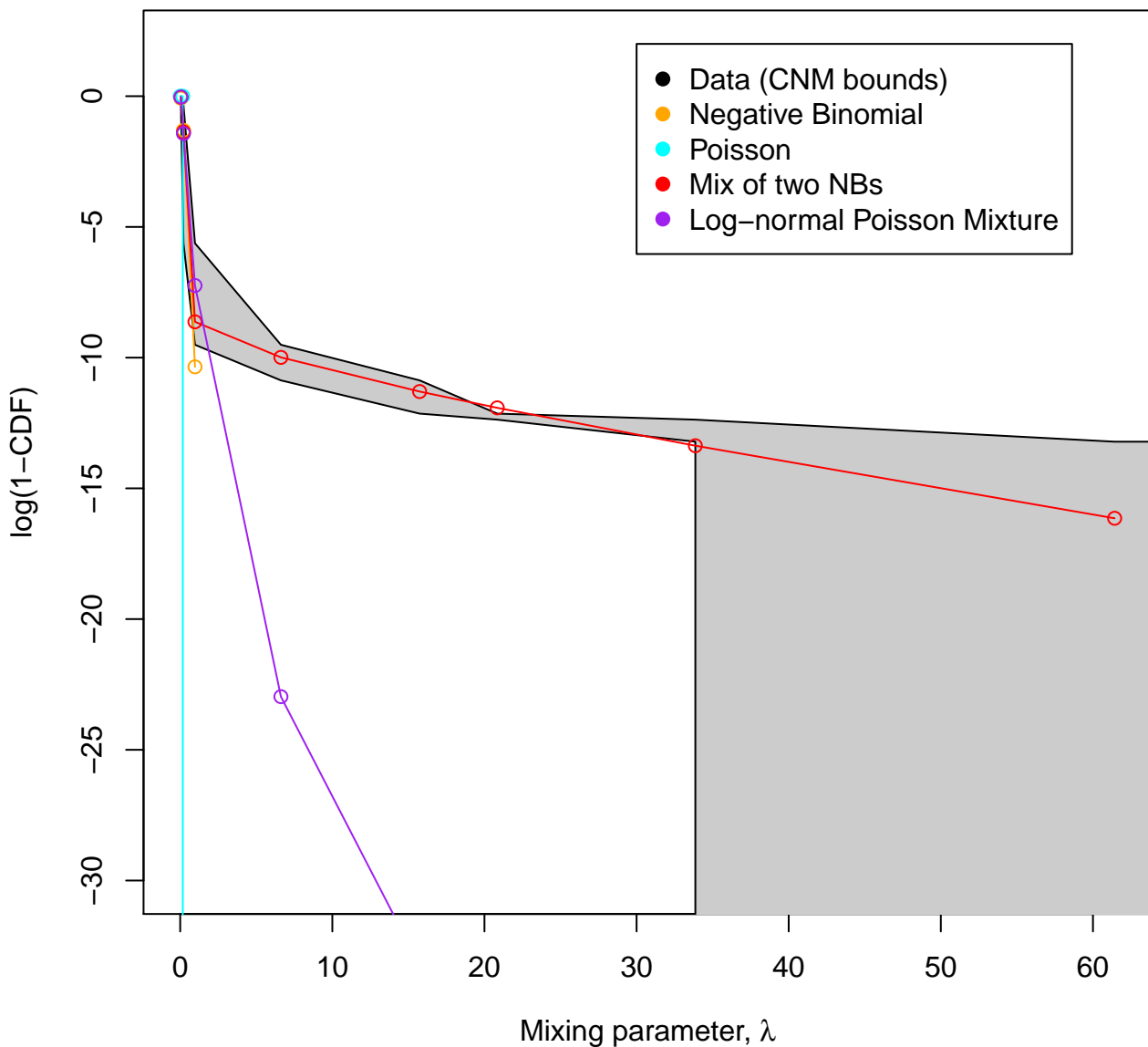

Supplement: Supplementary file 1 [file DataSheet1.ZIP › plots/s1Amix.pdf]

# Sample D

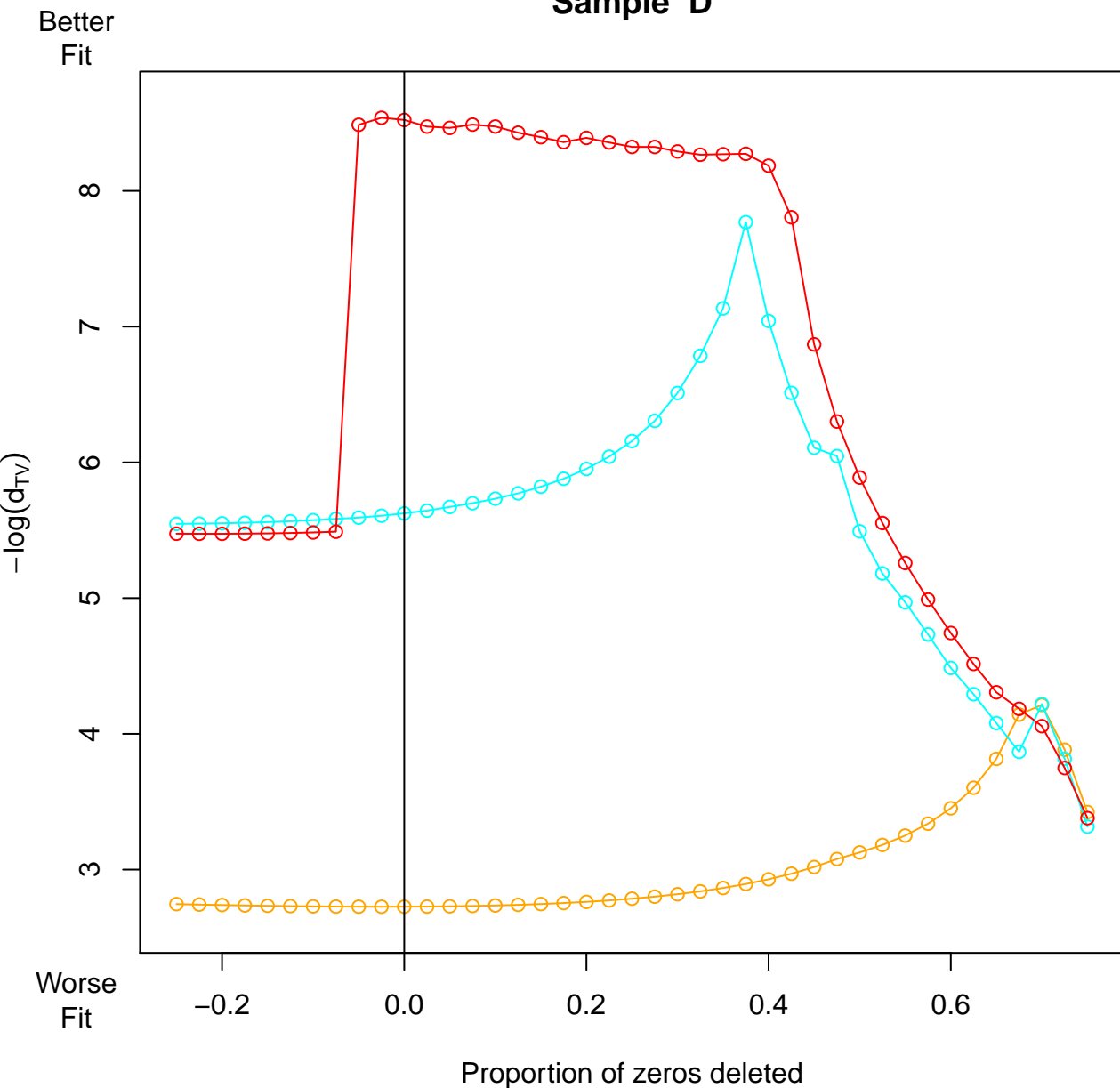

Supplement: Supplementary file 1 [file DataSheet1.ZIP › plots/s4TV.pdf]

# Sample D-NoDup

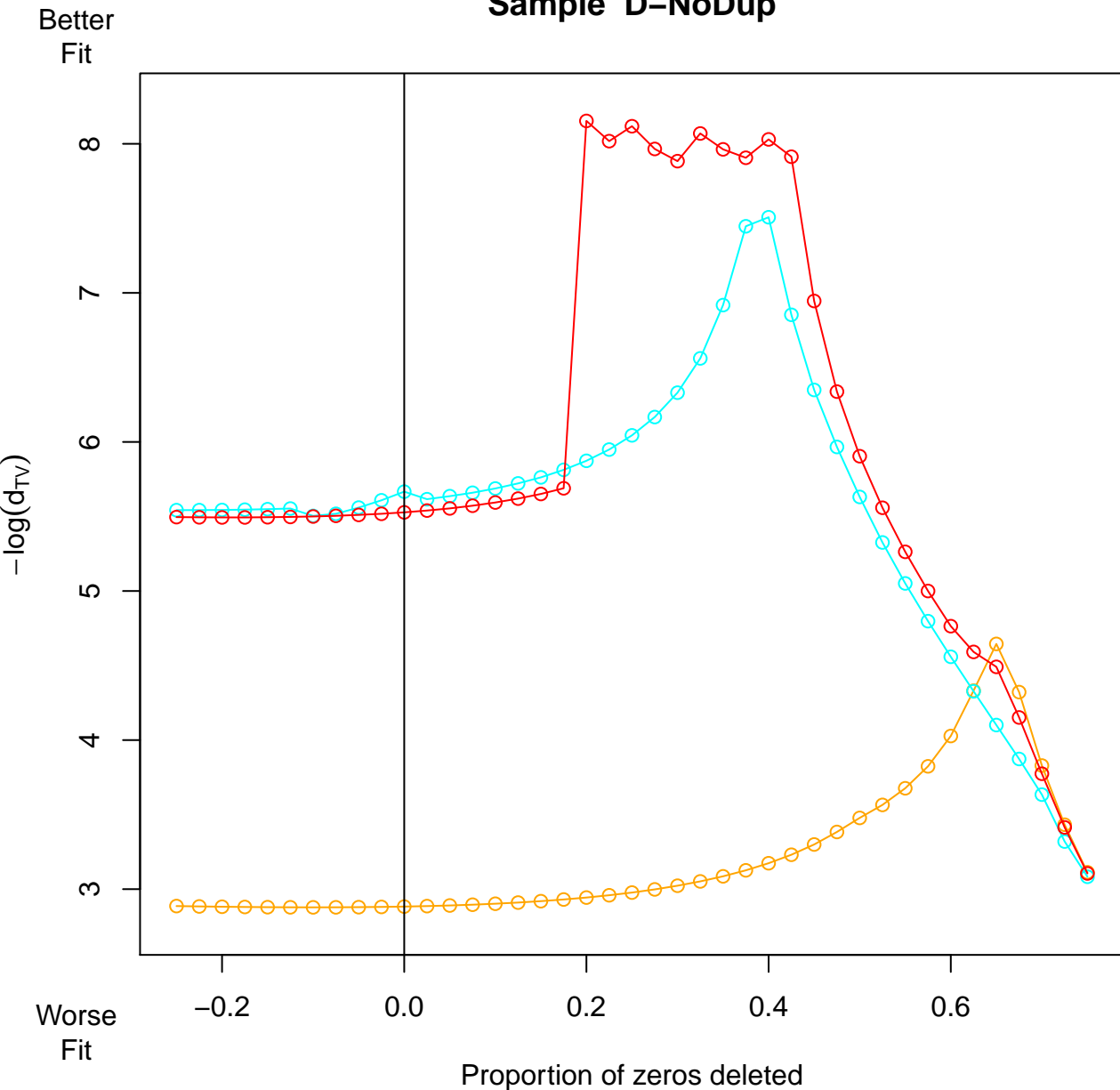

Supplement: Supplementary file 1 [file DataSheet1.ZIP › plots/s12TV.pdf]

## Sample A-NoDup

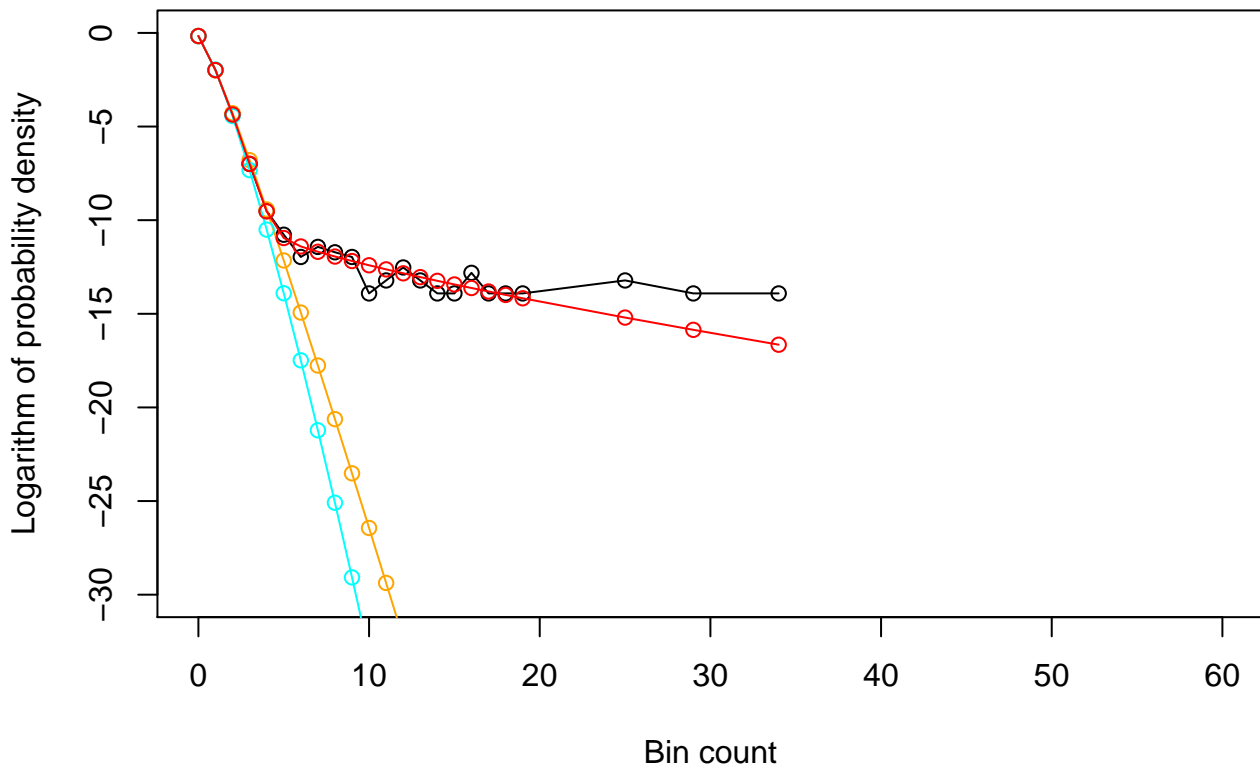

Supplement: Supplementary file 1 [file DataSheet1.ZIP › plots/s9Acount.pdf]
