# Supplementary material for: Quantifying the impact of inter-site heterogeneity on the distribution of ChIP-seq data
Source: Front Genet. 2014 Nov 14;5:399. doi: 10.3389/fgene.2014.00399 (PMC4231950; doi:10.3389/fgene.2014.00399)
Supplement: Supplementary file 2 [file DataSheet2.DOC]

The file SupplementalDataBedFiles.tar.gz is large and not required for analysis, unless regenerating the count tables from the original bed files. The file can be downloaded from:

http://www.compbio.group.cam.ac.uk/publications/supplementary-material/quantifying-the-impact-of-inter-site-heterogeneity-on-the-distribution-of-chip-seq-data
